# Supplementary material for: Cultural framing of giftedness in recent US fictional texts
Source: PLoS One. 2024 Aug 29;19(8):e0307222. doi: 10.1371/journal.pone.0307222 (PMC11361604; doi:10.1371/journal.pone.0307222)
Supplement: S1 File — Contains all instructions and code needed to replicate our study. (PDF) [file pone.0307222.s001.pdf]

S1 File.
Technical Supplement to *Cultural Framing of Giftedness in Recent US Fictional Texts* (Version 2)

Daniel Patrick Balestrini and Heidrun Stoeger

June 25, 2024

Contents

1 Introduction **2**

2 Data Availability Statement **2**

3 Preliminaries **2**

4 Packages **3**

5 Corpus Ingestion and Description **6**

6 Isolation of *gift and *only* Passages by Year** **19**

7 Corpus Consolidation **41**
7.1 Combining the Corpora 41
7.2 Removing Left-Over HTML Markup etc. 43

8 Corpus Preprocessing **56**

9 Keyword Identification **71**

10 Theme Identification **88**
10.1 Determining a Document-Exclusion Threshold 89
10.2 Preparing and Implementing the Theme-Identification Procedure 102

11 Validation **119**

12 Sentiment Analysis **144**

13 Additional Operations for Manuscript Preparation **162**

1 Introduction

The aim of this supplement is to describe in detail the implementation of the study described in our manuscript "Cultural framing of giftedness in recent US fictional texts" and thereby to facilitate replication of our study. This is a revised version of the Technical Supplement of 20 February 2024.

2 Data Availability Statement

The US fictional-texts data we used for this study are from the Corpus of Contemporary American English (COCA). References are provided for the COCA in the main manuscript. We used a version of the COCA licensed and downloaded in early 2020. We cannot share the corpus data due to license restrictions. However, users should be able to license the same COCA data via the site hosting the COCA [16]. As of 18 February 2024, the COCA data were still available for licensing and downloading at that site.

We created a repository with data sets used when conducting and reporting out study. It contains all of the data sets we used for calculating all values reported in our study with the exception of the text files and derivative data sets containing passages from the COCA. During the manuscript-review process, we reran all code with slight adjustments. Version 2 of the repository contains the updated data files.

Most of the analyses we describe in this document in detail can be verified using the data available in our repository. Once users have licensed and downloaded the COCA and extracted the relevant text files, as described in Section 5, it will be possible to replicate our entire study from the ground up by following the instructions in this document.

3 Preliminaries

We assume that people replicating our study will be familiar with using R.

For importing and exporting data in and out of the R environment created in this code, users should first set up a local R project. Those wishing to implement the code will also need the following directories within their local project folder, as they will be presupposed to exist in the following code:

- `R_Objects\COCA_Description`
- `R_Objects\Corpora`
- `R_Objects\Corpora\AllGiftedPassages`

- `R_Objects\Corpora\ManualReview`
- `R_Objects\Keywords`
- `R_Objects\PCA`
- `R_Objects\Sentiments`
- `R_Objects\ThemesValidation`

We also recommend making two preliminary customizations to the R environment, that is, turning off scientific notation and allowing large series to be printed to the console for inspection.

Listing 3.1: Preliminaries

```
# Turn off scientific notation and allow for more decimal places.

options(scipen = 999)

options(digits = 12)

# Print large series to the console.

\begin{myrcode}[label={lst:Printing Large Series}]{Printing Large Series}
options("max.print" = 1000000)

getOption("max.print")
```

4 Packages

The final execution of the code was conducted by the first author in June 2024 using R version 4.3.3 (2024-02-29 ucrt) and RStudio version 2024.4.2.764. This was performed on a system with an x86_64 architecture (64-bit), specifically a Windows 10 x64 platform (build 19045).

Before we installed the required packages, we installed the current version (4.3) of RTools on our Windows 10 PC. RTools is necessary on Windows systems for compiling some of the packages mentioned below. RTools is not an R package, but a collection of tools and compilers needed for working with R packages on Windows systems. It can be installed via the Rtools43 installer.

The following packages were required for various parts of the code:

- Scales for proper formatting of the y-axis values in a plot (version 1.3.0; [35])
- Stringr for tokenization needed for the initial description of the sample and for subsetting character vectors (version 1.5.1; [33])
- Readtext for importing text files (version 0.91; [6])
- Tidyverse for creating new variables etc. and for using the ggplot2() function (version 2.0.0; [36])
- Reticulate wrapper package for working with Python within R (version 1.35.0; [32])
- Spacyr wrapper package for working with the Python-based spaCy National Language Processing library within R (version 1.3.0; [5])
- Writexl for exporting results to Excel files with no dependencies (version 1.5.0; [23])
- Stopwords for removing stop words (version 2.3; [29])
- Psych for principal component analysis (version 2.4.1; [27])
- Nortest for testing normality with the Kolmogorov–Smirnov Test with the Lilliefors Correction (version 1.0-4; [17])
- Car for testing for equality of variances (version 3.1-2; [15])
- Devtools for installing the spacyr package (version 2.4.5; [34])
- Quanteda for text mining (version 3.3.1; [8])
- Quanteda.textstats for the textstat_keyness() function (version 0.96.4; [9])
- Openxlsx for exporting results to Excel files (version 4.2.5.2; [28])
- Wordnet for comparing keywords' synonyms (version 0.1-16; [13])
- RJava for accessing locally saved Wordnet [14, 26] data (version 1.0-11; [31])
- Pheatmap for producing a heatmap (version 1.0.12; [19])
- GridExtra for adding a margin around the heatmap plot (version 2.3; [2])
- Modeest for calculating a modal value (version 2.4.0; [25])
- Effsize for calculating Cohen's *d* and Cliff's delta (version 0.8.1; [30])

With the exception of the gridExtra, modeest, rJava, reticluate, scales, spacyr, and wordnet packages, all packages can be installed and loaded at once prior to running all of the code.

Listing 4.1: Installing and Loading Most Packages

```
install.packages("scales", dependencies = TRUE)

install.packages("stringr", dependencies = TRUE)

install.packages("readtext", dependencies = TRUE)

install.packages("tidyverse")

install.packages("writexl")

install.packages("stopwords")

install.packages("psych", dependencies = TRUE)

install.packages("nortest")

install.packages("car")

install.packages("devtools")

install.packages("quanteda")

install.packages("quanteda.textstats", dependencies = TRUE)

install.packages("openxlsx")

install.packages("reticulate")

install.packages("wordnet", dependencies = TRUE)

install.packages("rJava")

install.packages("pheatmap")

install.packages("gridExtra")

install.packages("modeest")

```

```
install.packages("effsize")

devtools::install_github("quanteda/spacyr")

library(stringr)

library(readtext)

library(tidyverse)

library(writexl)

library(stopwords)

library(psych)

library(nortest)

library(car)

library(quanteda, verbose = TRUE)

library(quanteda.textstats)

library(openxlsx)

library(pheatmap)

library(effsize)
```

The scales [35], gridExtra [2], and modeest [25] packages should only be loaded when needed and immediately detached due to conflicts with the psych package. They will be included at their point of use, below. Furthermore, we load the reticulate [32], spacyr [5], rJava [31], and wordnet [13] packages at their point of use due to the specifics of the order in which they are to be loaded.

5 Corpus Ingestion and Description

Once the COCA data have been licensed and downloaded, the user must find 29 text files containing the COCA fictional-text samples. There is one text file per year for the period 1990–2017, with the exception of 2012, for which the data are divided into two files. In the 2020 version of the COCA we used, the relevant files are located in the following folders:

- The first 22 annual samples and the first half of the sample for 2012 are located in the following directory: `\COCA\Texts\Fiction\text_fiction_awq`.
- The second half of 2012 is located in `\COCA\Texts\Update Jul 2012 to Dec 2015\text_2012-2015_ksr`.
- The final 5 annual samples, for the years 2013–2017, are located in `\COCA\Texts\Update Jan 2016 to Dec 2017\coca2017_text_qpj`.

We excerpted the 29 fiction files, which always started with the prefix `fic_`, and combined the two text files for 2012 into one file with the file-naming convention used in the other 27 files, that is, `fic_2012.txt`. Finally, we saved the resulting 28 files in our working directory for this study. Once these steps have been accomplished, users will have all of the data necessary for reproducing from the ground up all of the code used for this study.

Import the COCA fiction subcorpus files for the period 1990–2017 into the R environment. First, get the names of the files and store these in a character vector. Second, load each file with its original name and rename it with an ending indicative of a character vector (_v).

Listing 5.1: Ingesting the COCA Fiction Subcorpus

```
COCA_files <- list.files(pattern = "fic_\\d{4}\\.txt$")

for (file in COCA_files) {

  name <- sub("\\.txt$", "_v", file)

  assign(name, readLines(con = file))

}

class(fic_1990_v)
```

Count how many lines each annual text sample has and describe the counts. Each line in each annual character vector reflects a sample from a different fictional text in the COCA fiction subcorpus from a given year.

Listing 5.2: Counting Annual Text Samples

```
# Create a vector with the 28 object names.

object_names <- paste0("fic_", 1990:2017, "_v")

# Create a vector for storing the lengths of each object.

COCA_fic_line_counts <- numeric(length(object_names))

# Loop through each object name, get the object, assess its length, and
    store its length.

for (i in 1:length(object_names)) {

  current_obj <- get(object_names[i])

  COCA_fic_line_counts[i] <- length(current_obj)

}

# Calculate combined line counts of all 28 character vectors.

docs_in_COCA_fiction_corpus <- sum(COCA_fic_line_counts)

print(docs_in_COCA_fiction_corpus)

# Calculate the average number of sampled texts per year.

Average_texts_year <- mean(COCA_fic_line_counts)

print(Average_texts_year)

# Calculate the standard deviation (SD) for the sampled texts per year.

SD_texts_year <- sd(COCA_fic_line_counts)

print(SD_texts_year)
```

Describe how many tokens each annual file and each line in each annual file has.

Listing 5.3: Describing Annual Samples

```
# Create a vector for storing the number of tokens for each annual
    object.

COCA_fic_token_counts <- numeric(length(object_names))

# Loop through each object by way of a vector of object names, tokenize
    its content, and store its token count. Use the str_split() function
    with the 'boundary("word")' argument from the stringr package to
    tokenize each character vector by word and then use the unlist()
    function to put the thus tokenized character vectors (not lists) back
     into a character vector, in which each token is set in quotes.

for (i in 1:length(object_names)) {

  current_obj <- get(object_names[i])

  tokens <- unlist(str_split(current_obj, boundary("word")))

  COCA_fic_token_counts[i] <- length(tokens)

}

# Calculate combined token counts of all 28 character vectors.

tokens_in_COCA_fiction_corpus <- sum(COCA_fic_token_counts)

print(tokens_in_COCA_fiction_corpus)

# Calculate the average number of tokens per year.

Average_tokens_year <- mean(COCA_fic_token_counts)

print(Average_tokens_year)

# Calculate the standard deviation (SD) for the tokens per year.

SD_tokens_year <- sd(COCA_fic_token_counts)

```

```
print(SD_tokens_year)

# Calculate the average length and standard deviation (SD) of each text sample (i.e., each line in each character vector).

# Create an empty numeric vector to store word counts for each line.

line_word_counts <- numeric()

# Loop through the tokenized character vectors and count the tokens per line. This entails the following steps: Loop through each object name, tokenize its content, and store its token count. Use the str_split() function with the 'boundary("word")' argument from the stringr package to tokenize each character vector by word, and then use the unlist() function to put the thus tokenized character vectors (not lists) back into a character vector, in which each token is set in quotes. Append word counts to the overall vector. After running the function, line_word_counts will contain the word count for each line across all files.

for (obj_name in object_names) {

  current_obj <- get(obj_name)

  current_word_counts <- sapply(current_obj, function(line) {

    tokens <- unlist(str_split(line, boundary("word")))

    length(tokens)

  })

  line_word_counts <- c(line_word_counts, current_word_counts)

}

average_word_count <- mean(line_word_counts)

print(average_word_count)

sd_word_count <- sd(line_word_counts)

```

```
print(sd_word_count)
```

Gather together descriptive statistics for the sample.

Listing 5.4: Gathering Together Sample Descriptive Statistics

```
descriptive_stats <- tibble(
  Metric = c("Total documents 1990-2017",
    "Average documents per year",
    "SD for documents per year",
    "Total tokens 1990-2017",
    "Average tokens per year",
    "SD for tokens per year",
    "Average tokens per text sample",
    "SD for tokens per text sample"
  ),
  Value = c(
    formatC(docs_in_COCA_fiction_corpus, format = "f", big.mark = ",", digits = 0),
    formatC(Average_texts_year, format = "f", big.mark = ",", digits = 2),
    formatC(SD_texts_year, format = "f", big.mark = ",", digits = 2),
    formatC(tokens_in_COCA_fiction_corpus, format = "f", big.mark = ",",
```

```
          digits = 0),
      formatC(Average_tokens_year, format = "f", big.mark = ",", digits = 2),
      formatC(SD_tokens_year, format = "f", big.mark = ",", digits = 2),
      format(average_word_count, format = "f", big.mark = ",", digits = 2),
      format(sd_word_count, format = "f", big.mark = ",", digits = 2)
    )
)
print("Descriptive Statistics by Annual Sample")
print(descriptive_stats)
```

Review unusually short and unusually long texts.

Listing 5.5: Reviewing Unusually Short and Long Texts

```
# Create a data frame to store the text ID, its corresponding token count, and the actual text.
TextTokenCounts <- data.frame(
  Year = integer(),
  TextID = integer(),
  TokenCount = integer(),
  TextContent = character()
```

```r
)

for (year in 1990:2017) {

  obj_name <- paste0("fic_", year, "_v")

  current_obj <- get(obj_name)

  for (text_id in 1:length(current_obj)) {

    line <- current_obj[text_id]

    tokens <- unlist(str_split(line, boundary("word")))

    token_count <- length(tokens)

    new_row <- data.frame(

      Year = year,

      TextID = text_id,

      TokenCount = token_count,

      TextContent = line

    )

    TextTokenCounts <- rbind(TextTokenCounts, new_row)

  }

}

# Review the 10 shortest texts.

shortest_token_counts <- TextTokenCounts %>%

  arrange(TokenCount) %>%

  slice_head(n = 10)

```

```
# Review the three longest texts.

longest_token_counts <- TextTokenCounts %>%

  arrange(desc(TokenCount)) %>%

  slice_head(n = 3)

# Display the results

print("Top␣10␣Shortest␣Token␣Counts:")

print(shortest_token_counts)

print("Top␣3␣Longest␣Token␣Counts:")

print(longest_token_counts)
```

Plot the lengths (token counts) of all texts in the COCA fiction subcorpus with ggplot2 package.

Before implementing the plot, load the scales package [35], which is needed for proper formatting of the y-axis values in the plot. As the scales package conflicts with the psych package, we only load it here, and detach it once we are done using it.

Listing 5.6: Loading the Scales Package

```
library(scales)
```

Now create a box plot with 'jittered' points [11] to visualize the distribution of token counts among the texts in the COCA fiction subcorpus.

Listing 5.7: Plotting Token Counts

```
boxplot_jitter_figure <- ggplot(TextTokenCounts, aes(x = factor(0), y =
    TokenCount)) +

  # Call the jitter function with semitransparent colored circles to
      represent token counts.

  geom_jitter(width = 0.5, alpha = 0.4, size = 1.0, color = "#E34234") +

  # Call the actual box plot on top of the 'jittered' circles. Suppress
      the automatic display of outliers on the central vertical line,
      because these are displayed by the 'jittered' dots. Also, call the
      color 'black' for the box plot; otherwise, it appears in gray in
      the PDF rendering.

  geom_boxplot(width = 1, outlier.shape = NA, fatten = 2, size = 1, fill
       = NA, color = "black") +

  # Use a log-10 scale for the y-axis.

  scale_y_log10(labels = comma, breaks = trans_breaks("log10", function(
      x) 10^x)) +

  labs(x = "", y = "Token␣Count␣(Log10)") +

  theme_minimal() +

  # Set the y-axis text to 'black'.

  theme(axis.text.y = element_text(color = "black"),

    # Remove the x-axis title.

    axis.title.x = element_blank(),

    # Remove the x-axis text.

    axis.text.x = element_blank(),

```

```
    # Remove the x-axis ticks.

    axis.ticks.x = element_blank())

# Print the boxplot.

print(boxplot_jitter_figure)
```

Export the plot in various formats.

Listing 5.8: Exporting the Plot

```
# Export as PDF.

ggsave(filename = "Fig1.pdf",

  plot = boxplot_jitter_figure,

  device = "pdf",

  path = "R_Objects/COCA_Description",

  width = 13.2, height = 9.28, units = "cm")

# Export as TIFF.

ggsave(filename = "Fig1.tiff",

  plot = boxplot_jitter_figure,

  device = "tiff",

  path = "R_Objects/COCA_Description",

  width = 13.2, height = 9.28, units = "cm", dpi = 300)

# Export as PNG.
```

```
ggsave(filename = "Fig1.png",

  plot = boxplot_jitter_figure,

  device = "png",

  path = "R_Objects/COCA_Description",

  width = 13.2, height = 9.28, units = "cm",

  dpi = 300)
```

Clean up the global environment.

Listing 5.9: Cleaning up the Global Environment After Corpus Ingestion and Description

```
# Detach the scales package by detaching the ggplot2 package, which is
    also no longer needed.

detach("package:ggplot2", unload = TRUE)

# Save the objects needed for later steps and/or for auditing our work.

# Start by saving the 28 character vectors with the annual COCA
    fictional texts. To this end, first create a vector of object names.

object_names <- paste0("fic_", 1990:2017, "_v")

# Now loop over the object names and save each object.

for (name in object_names) {

  object_to_save <- get(name)

  file_name <- paste0("R_Objects/Corpora/", name, ".rds")

```

```
  saveRDS(object_to_save, file_name)

}

# Now save all other relevant objects.

saveRDS(COCA_fic_line_counts, "R_Objects/COCA_Description/COCA_fic_line_counts.rds")

saveRDS(COCA_fic_token_counts, "R_Objects/COCA_Description/COCA_fic_token_counts.rds")

saveRDS(line_word_counts, "R_Objects/COCA_Description/line_word_counts.rds")

saveRDS(TextTokenCounts, "R_Objects/COCA_Description/TextTokenCounts.rds")

saveRDS(boxplot_jitter_figure, "R_Objects/COCA_Description/boxplot_jitter_figure.rds")

# Retrieve the names of all objects in the global environment and store them in a character vector.

all_objects <- ls()

# Create a character vector of object names in the global environment.

formatted_object_array <- paste('c("', paste(all_objects, collapse = '", "'), '")', sep = "")

# Export a formatted list to a .txt file for manual review.

write(formatted_object_array, "R_objects.txt")

# Manually review the .txt file and delete objects to be kept in the R global environment.

# Import the first line of the modified .txt file back into R.

string_of_object_names <- readLines("R_objects.txt", n = 1)

```

```
# Convert the string in the .txt file back into a character vector of
   object names.

formatted_object_array <- eval(parse(text = string_of_object_names))

# Remove the objects selected for removal from the global environment.

rm(list = c(formatted_object_array))

rm(list = c("all_objects", "formatted_object_array", "string_of_object_
   names"))

# Invoke the garbage collection function gc() to release memory occupied
    by no-longer-used objects and to compact the storage of remaining
   objects in the global environment to improve memory usage.

gc()
```

6 Isolation of *gift** and *only* Passages by Year

Isolate all passages containing *gift** as well as a comparison sample of all passages containing *only* by year.

First, load the 28 annual fictional-text samples into the R global environment.

Listing 6.1: Loading the Annual Fictional-Text Samples

```
# Create the vector of object names.

object_names <- paste0("fic_", 1990:2017, "_v")

# Loop over the object names and read each object into the R global
   environment.

for (name in object_names) {

  file_name <- paste0("R_Objects/Corpora/", name, ".rds")
```

```
  assign(name, readRDS(file_name))

}
```

Second, convert each annual data frame into a separate Quanteda corpus object.

Listing 6.2: Converting Annual Data Frames into Quanteda Corpus Objects

```
# Loop over each data frame name and reformat each character vector as a
    Quanteda corpus object.

for (name in object_names) {

  # Construct the corpus.

  corpus_object <- quanteda::corpus(get(name))

  # Construct a new object name for the corpus, removing "_v" from the
     name.

  corpus_name <- sub("_v$", "_corpus", name)

  # Assign the corpus object to a new variable with the constructed name
     .

  assign(corpus_name, corpus_object)

}

str(fic_2017_corpus)
```

Third, customize the document names and add one document-level variable (docvar) for the year.

Listing 6.3: Customizing Document Names

```
# Create an object with the names of the 28 Quanteda corpus objects.

quanteda_corpus_names <- ls(pattern = "fic_\\d{4}_corpus")

# Loop over each corpus name.

for (name in quanteda_corpus_names) {

  # Get the corpus object.

  corpus_object <- get(name)

  # Extract the year from the name.

  year <- sub("fic_([0-9]{4})_corpus", "\\1", name)

  # Reassign the document names to reflect source, sample type, and year.

  docnames(corpus_object) <- paste("COCA_fic_", year, "_", 1:ndoc(corpus_object), sep = "")

  # Add year as a document-level variable.

  docvars(corpus_object, "year") <- as.integer(year)

  # Assign the modified corpus back to the original variable.

  assign(name, corpus_object)

}

str(fic_1990_corpus)

summary(fic_1990_corpus)

class(fic_1990_corpus)

```

```
names(fic_1990_corpus)

View(fic_1990_corpus)

head(docvars(fic_1990_corpus))

length(fic_1990_corpus)
```

Fourth, tokenize the corpus object.

Listing 6.4: Tokenizing the Corpus Object

```
# Create a list of corpus names. Use the object with the names of the 28 Quanteda corpus objects created in the previous step (quanteda_corpus_names). Loop over each Quanteda corpus object and tokenize each one.

for (name in quanteda_corpus_names) {

  # Get the corpus object.

  corpus_object <- get(name)

  # Tokenize the corpus.

  tokens_object <- quanteda::tokens(corpus_object, verbose = TRUE)

  # Construct a new object name for the tokens.

  tokens_name <- sub("_corpus$", "_tokens", name)

  # Assign the tokens object to a new variable with the constructed name.

  assign(tokens_name, tokens_object)

}
```

```

class(fic_2017_tokens)

str(fic_2017_tokens)
```

Fifth, excerpt 51-word and 201-word passages centered on all instances of *gift** and *only*. We also call these "text windows."

Before starting, remove the character vectors and the Quanteda corpus objects to free up memory in the R global environment.

Listing 6.5: Excerpting the *gift** Passages

```
rm(list = c(object_names, quanteda_corpus_names))
```

Start by excerpting the *gift** passages.

Listing 6.6: Excerpting the *gift** Passages

```
# Create an empty data frame to store the results and include a Keepers
  variable with the factor levels 'yes' and 'no' for a subsequent step.

gift_passages_all <- data.frame(

  Year = integer(),

  Pre25 = character(),

  Node_word1 = character(),

  Post25 = character(),

```

```
  Pre100 = character(),
  Node_word2 = character(),
  Post100 = character(),
  Keepers = factor(character(0), levels = c('yes', 'no')),
  stringsAsFactors = FALSE
)
# Loop through each tokens object, including an 'NA' placeholder for the Keepers variable for now, and use the kwic() function to search for instances of 'gift*' and excerpt 51-word and 201-word windows of text around all such strings.
token_names <- ls(pattern = "fic_\\d{4}_tokens")
for (name in token_names) {
  tokens_object <- get(name)
  year <- sub("fic_([0-9]{4})_tokens", "\\1", name)
  # Apply the kwic() function with window sizes of 25 and 100.
  kwic_25 <- kwic(tokens_object, window = 25, pattern = "gift*")
  kwic_100 <- kwic(tokens_object, window = 100, pattern = "gift*")
  # Loop through the results and extract the information.
  for (i in 1:nrow(kwic_25)) {
  Pre25 <- paste(kwic_25$pre[i], collapse = "␣")
  Node_word1 <- paste(kwic_25$keyword[i], collapse = "␣")
  Post25 <- paste(kwic_25$post[i], collapse = "␣")
  Pre100 <- paste(kwic_100$pre[i], collapse = "␣")
```

```
  Node_word2 <- paste(kwic_100$keyword[i], collapse = "␣")
  Post100 <- paste(kwic_100$post[i], collapse = "␣")
  Keepers <- NA
  # Add the results to the final data frame.
  gift_passages_all <- rbind(gift_passages_all, data.frame(
    Year = as.integer(year),
    Pre25 = Pre25,
    Node_word1 = Node_word1,
    Post25 = Post25,
    Pre100 = Pre100,
    Node_word2 = Node_word2,
    Post100 = Post100,
    Keepers = Keepers,
    stringsAsFactors = FALSE
    ))
  }
}
str(gift_passages_all)
dim(gift_passages_all)
View(gift_passages_all)
names(gift_passages_all)
```

```
length(gift_passages_all)

gift_passages_all$Pre100[1]

# Add a variable that counts all the cases from each year for use when
    we remove cases unrelated to giftedness (described below).

gift_passages_all <- gift_passages_all %>%

  group_by(Year) %>%

  mutate(Annual_Count = row_number()) %>%

  ungroup()

# Reorder the columns to place Annual_Count after Year.

gift_passages_all <- gift_passages_all %>%

  select(Year, Annual_Count, everything())

names(gift_passages_all)

# Reorder the columns to move Keepers between the short and long text
    windows.

gift_passages_all <- gift_passages_all %>%

  select(Year, Annual_Count, Pre25, Node_word1, Post25, Keepers,
     everything())

names(gift_passages_all)
```

Export the `gift_passages_all` data frame to Excel for easier reviewing and coding.

Listing 6.7: Exporting the gift* Passages to Excel

```
write_xlsx(gift_passages_all, "R_Objects/Corpora/AllGiftedPassages/gift_
    passages_all.xlsx", col_names = TRUE, format_headers = TRUE, use_
    zip64 = FALSE)
```

In Excel, manually code the Keepers variable to identify whether each text window (i. e., a contiguous text passage) reflects (a) *gift** being used in the sense of ‘gift’ as a noun or verb related to giftedness or (b) not. According to the online edition of *Merriam-Webster’s Dictionary* entry on ‘gift,’ [22] this means the following:

1. ‘gift’ noun 1: a notable capacity, talent, or endowment (yes)
2. ‘gift’ noun 2: something voluntarily transferred by one person to another without compensation (no)
3. ‘gift’ noun 3: the act, right, or power of giving (no)
4. ‘gift’ verb 1: to endow with some power, quality, or attribute (yes)
5. ‘gift’ verb 2: to make a gift of (no)

Review all the passages in Excel to determine which ones are ‘keepers’ as per the above definition. To make the manual review of all 9,151 rows easier, add the code in Listing 6.8 to the Microsoft Visual Basic editor within Excel.

Listing 6.8: Customizing the Excel File

```
Private Sub Workbook_SheetSelectionChange(ByVal Sh As Object, ByVal
    Target As Range)

Cells.Interior.ColorIndex = xlNone

ActiveCell.EntireRow.Interior.ColorIndex = 8

End Sub
```

The Microsoft Visual Basic code in Listing 6.8 highlights in a special color the row on which a cell is opened. This was very helpful for reading through and coding the 9,151 text passages.

In most cases, a contextual determination of the meaning of *gift** is possible by reading the 51-word text windows. If the categorization is not clear after reading the short text window, also read the 201-word text window (further to the right in the Excel file). If the meaning of *gift** remains unclear, code with ‘no.’

Export the Keepers column from Excel to a .txt file (with no header) called Keepers.txt with utf-8 encoding. We kept line 9,152 as an empty line; this may differ for different operating systems. Our Keepers file is included as a supporting document so those replicating our work can compare our categorization against their own and also verify our exact findings.

Load the resulting column back into R.

Listing 6.9: Loading Keepers Column into R

```
keepers_vector <- readLines("R_Objects/Corpora/AllGiftedPassages/Keepers
    .txt")
```

Check to make sure the newly imported `keepers_vector` and the existing `gift_passages_all$Keepers` vector have the same length, and use the unique() function to make sure that no typos were made when coding the 9,151 text windows. This happens almost invariably during the manual coding process and absolutely must be corrected for the next subsetting step to work properly. One could also set up a dropdown menu in the Excel file to ensure that one enters only “yes” or “no.” However, we found that the steps involved in selecting an option via a dropdown menu in Excel slowed down data entry considerably.

Listing 6.10: Checking Keepers Vectors

```
length(keepers_vector)

length(gift_passages_all$Keepers)

unique(keepers_vector)

# If coding contains no typos, then the only unique values are 'no' and
    'yes'. Otherwise, fix, recheck, and then move on. Then overwrite the
```

```
    Keepers vector in gift_passages_all using the new keepers_vector.

gift_passages_all$Keepers <- keepers_vector

str(gift_passages_all)

dim(gift_passages_all)

head(gift_passages_all)

View(gift_passages_all)
```

Corroborate the replicability of the manual coding procedure. Do so by (a) creating a systematic sample of 200 cases from the `gift_passages_all` object (i. e., 200 cases spread out evenly across the entire object), (b) exporting the resulting object to a separate Excel file, and (c) instructing an external rater with knowledge of US fictional texts to independently replicate the rating process carried out by the first author. We now describe this process in detail.

First, extract a systematic sample of 200 cases from the `gift_passages_all` object and export this to an Excel file for use in the external rating process.

Listing 6.11: Preparing a Sample for External Rating

```
# Count the total cases in 'gift_passages_all' and set the sample size
    for the new sample to 200.

total_cases <- nrow(gift_passages_all)

sample_size <- 200

# Generate a sequence of indices evenly distributed across the total
    cases.

IRR_Keepers <- seq(1, total_cases, length.out = sample_size)

# Round the indices to the nearest integer to get valid case indices.

```

```
IRR_Keepers <- round(IRR_Keepers)

# Display the indices of the cases to keep.

print(IRR_Keepers)

# Subset the original data frame to get the sampled cases.

gift_passages_all_IRR <- gift_passages_all[IRR_Keepers, ]

# Export the 'gift_passages_all_IRR' data frame to Excel for the independent external coding process.

write_xlsx(gift_passages_all_IRR, "R_Objects/Corpora/AllGiftedPassages/gift_passages_all_IRR.xlsx", col_names = TRUE, format_headers = TRUE, use_zip64 = FALSE)
```

Second, prepare the resulting Excel file for ease of use by the external rater. Add the Microsoft Visual Basic code shown in Listing 6.8 to the Excel file and save the Excel file as an `.xlsm` file. Prepare instructions for the external rater that precisely reflect the categorization process used by the first author as described on p. 27 of this document. The instruction file we sent to our external rater is included in the data repository (`Inter-Rater Request Letter_Eschborn.pdf_redacted`). The letter is redacted because we agreed with the rater to not share his email address in the version of the letter we save in our article repository.

Third, elicit independent ratings (i. e., categorizations) of the 200-passage sample from a person with knowledge of US English as well as knowledge of US fictional texts. Our independent rater had a doctorate in American Studies with a focus on US literature. The Excel file containing the external rater's categorizations is included in the data repository (see `gift_passages_all_IRR_Eschborn_completed.xlsm`).

After the external rating has been completed, export the Keepers column from the external rater's Excel file to a .txt file (with no header) called `KeepersExternal.txt` with utf-8 encoding. Please note: Our external rater inadvertently added one extra character space after their 'yes' response in column F on line 71 in the returned Excel file (with a header), which is found at line 70 in the text file `KeepersExternal.txt` (with no header). This extra space after 'yes' must be deleted.

Fourth, load the resulting external ratings as saved in `KeepersExternal.txt` back into R, create a matching subset of the already loaded `keepers_vector` object (i. e., for the internal ratings made by the first author), and compare the inter-rater agreement between the sample of cases categorized by the first author and the cases categorized by the independent external rater.

Listing 6.12: Comparing Agreement Between Internal and External Ratings

```
keepers_vector_external <- readLines("R_Objects/Corpora/
    AllGiftedPassages/KeepersExternal.txt")

# Subset the complete set of internal ratings, stored in the 'keepers_
    vector', to only keep the cases that were also externally rated.

keepers_vector_internal_sampled <- keepers_vector[IRR_Keepers]

# Check that both vectors have the same length.

length(keepers_vector_internal_sampled)

length(keepers_vector_external)

# Calculate the overall inter-rater agreement.

agreements <- sum(keepers_vector_internal_sampled == keepers_vector_
    external)

disagreements <- sum(keepers_vector_internal_sampled != keepers_vector_
    external)

overall_agreement <- agreements / (agreements + disagreements)

print(paste("Overall␣agreement:", overall_agreement))

# Calculate Cohen's kappa, to express chance-corrected agreement.

# Create a data frame for the two raters' categorization decisions.

Ratings_df <- data.frame(rater1 = keepers_vector_internal_sampled,
    rater2 = keepers_vector_external)

# Calculate Cohen's kappa.

cohen_kappa <- cohen.kappa(Ratings_df)

print("Cohen's␣kappa:")
```

```

print(cohen_kappa)
```

Now excerpt the *only* passages.

Listing 6.13: Excerpting the *only** Passages

```
# Create an empty data frame to store the results.

only_passages_all <- data.frame(

  Year = integer(),

  Pre25 = character(),

  Node_word1 = character(),

  Post25 = character(),

  Pre100 = character(),

  Node_word2 = character(),

  Post100 = character(),

  stringsAsFactors = FALSE

)

# Loop through each tokens object and use the kwic() function to search
    for instances of 'only' and excerpt 51-word and 201-word windows of
    text around all such strings.

token_names <- ls(pattern = "fic_\\d{4}_tokens")

for (name in token_names) {

```

```
tokens_object <- get(name)
year <- sub("fic_([0-9]{4})_tokens", "\\1", name)
# Apply the kwic() function with window sizes of 25 and 100.
kwic_25 <- kwic(tokens_object, window = 25, pattern = "only")
kwic_100 <- kwic(tokens_object, window = 100, pattern = "only")
# Loop through the results and extract the information.
for (i in 1:nrow(kwic_25)) {
  Pre25 <- paste(kwic_25$pre[i], collapse = "␣")
  Node_word1 <- paste(kwic_25$keyword[i], collapse = "␣")
  Post25 <- paste(kwic_25$post[i], collapse = "␣")
  Pre100 <- paste(kwic_100$pre[i], collapse = "␣")
  Node_word2 <- paste(kwic_100$keyword[i], collapse = "␣")
  Post100 <- paste(kwic_100$post[i], collapse = "␣")
  # Add the results to the final data frame.
  only_passages_all <- rbind(only_passages_all, data.frame(
    Year = as.integer(year),
    Pre25 = Pre25,
    Node_word1 = Node_word1,
    Post25 = Post25,
    Pre100 = Pre100,
    Node_word2 = Node_word2,
```

```
      Post100 = Post100,
      stringsAsFactors = FALSE
    ))
  }
}
# Add a variable that counts all the cases from each year (to match gift_passages_all).
only_passages_all <- only_passages_all %>%
  group_by(Year) %>%
  mutate(Annual_Count = row_number()) %>%
  ungroup()
# Reorder the columns to place 'Annual_Count' after 'Year'.
only_passages_all <- only_passages_all %>%
  select(Year, Annual_Count, everything())
str(onyl_passages_all)
dim(only_passages_all)
names(only_passages_all)
length(only_passages_all)
only_passages_all$Pre100[1]
```

Sixth, describe the resulting *gift** and *only* text windows.

Calculate the average word-token count for the 51-word passages in both data frames together (using the stringr [33] and dpylr [36] packages).

Listing 6.14: Calculating Average Word Token Count (51)

```
# Compute the row-wise sums for the 'gift_passages_all' data frame.
intermediate_gift_passages51 <- gift_passages_all %>%
  rowwise() %>%
  mutate(
    Pre25_clean = str_replace(str_replace(Pre25, '^"', ''), '"$', ''),
    Node_word1_clean = str_replace(str_replace(Node_word1, '^"', ''), '"$', ''),
    Post25_clean = str_replace(str_replace(Post25, '^"', ''), '"$', '')
  ) %>%
  mutate(
    total_words = length(unlist(str_split(Pre25_clean, "\\s+"))) +
      length(unlist(str_split(Node_word1_clean, "\\s+"))) +
      length(unlist(str_split(Post25_clean, "\\s+")))
  )
str_count(gift_passages_all$Node_word1)
head(gift_passages_all$Node_word1)
# Compute the row-wise sums for the 'only_passages_all' data frame.
intermediate_only_passages51 <- only_passages_all %>%
  rowwise() %>%
  mutate(
```

```
    Pre25_clean = str_replace(str_replace(Pre25, '^"', ''), '"$', ''),
    Node_word1_clean = str_replace(str_replace(Node_word1, '^"', ''), '"$', ''),
    Post25_clean = str_replace(str_replace(Post25, '^"', ''), '"$', '')
  ) %>%
  mutate(
    total_words = length(unlist(str_split(Pre25_clean, "\\s+"))) +
      length(unlist(str_split(Node_word1_clean, "\\s+"))) +
      length(unlist(str_split(Post25_clean, "\\s+")))
  )
# Combine the total_words columns from both intermediary data frames into one object.
combined_word_counts51 <- c(intermediate_gift_passages51$total_words, intermediate_only_passages51$total_words)
# Calculate the mean and standard deviation of the combined word counts.
mean_word_length_first_corpus51 <- mean(combined_word_counts51)
sd_word_length_first_corpus51 <- sd(combined_word_counts51)
# Print the results.
print(paste("Mean word count 51: ", mean_word_length_first_corpus51))
print(paste("Standard Deviation of word count 51: ", sd_word_length_first_corpus51))
gift_passages_all$Node_word2[1]
str_count(gift_passages_all$Node_word2[1])
```

Calculate the average word-token count for the 201-word passages in both data frames together (using the stringr [33] and dpylr [36] packages).

Listing 6.15: Calculating Average Word Token Count (201)

```
# Compute the row-wise sums for the 'gift_passages_all' data frame.
intermediate_gift_passages201 <- gift_passages_all %>%
  rowwise() %>%
  mutate(
    Pre100_clean = str_replace(str_replace(Pre100, '^"', ''), '"$', ''),
    Node_word2_clean = str_replace(str_replace(Node_word2, '^"', ''), '"$', ''),
    Post100_clean = str_replace(str_replace(Post100, '^"', ''), '"$', '')
  ) %>%
  mutate(
    total_words = length(unlist(str_split(Pre100_clean, "\\s+"))) +
      length(unlist(str_split(Node_word2_clean, "\\s+"))) +
      length(unlist(str_split(Post100_clean, "\\s+")))
  )
str_count(gift_passages_all$Node_word2)
head(gift_passages_all$Node_word2)
# Compute the row-wise sums for the 'only_passages_all' data frame.
intermediate_only_passages201 <- only_passages_all %>%
```

```

  rowwise() %>%

  mutate(

    Pre100_clean = str_replace(str_replace(Pre100, '^"', ''), '"$', ''),

    Node_word2_clean = str_replace(str_replace(Node_word2, '^"', ''), '"$', ''),

    Post100_clean = str_replace(str_replace(Post100, '^"', ''), '"$', '')

  ) %>%

  mutate(

    total_words = length(unlist(str_split(Pre100_clean, "\\s+"))) +

      length(unlist(str_split(Node_word2_clean, "\\s+"))) +

      length(unlist(str_split(Post100_clean, "\\s+")))

  )

# Combine the total_words columns from both intermediary data frames into one object.

combined_word_counts201 <- c(intermediate_gift_passages201$total_words, intermediate_only_passages201$total_words)

# Calculate the mean and standard deviation of the combined word counts.

mean_word_length_first_corpus201 <- mean(combined_word_counts201)

sd_word_length_first_corpus201 <- sd(combined_word_counts201)

# Print the results.

print(paste("Mean␣word␣count␣201:␣", mean_word_length_first_corpus201))

print(paste("Standard␣Deviation␣of␣word␣count␣201:␣", sd_word_length_
```

```
    first_corpus201))

gift_passages_all$Node_word2[1]

str_count(gift_passages_all$Node_word2[1])
```

Clean up the global environment.

Listing 6.16: Cleaning up the Global Environment After Isolation Process

```
# Save the objects needed for later steps and/or for auditing our work.

saveRDS(gift_passages_all, "R_Objects/Corpora/gift_passages_all.rds")

saveRDS(keepers_vector, "R_Objects/Corpora/AllGiftedPassages/keepers_
    vector.rds")

saveRDS(IRR_Keepers, "R_Objects/Corpora/AllGiftedPassages/IRR_Keepers.
    rds")

saveRDS(gift_passages_all_IRR, "R_Objects/Corpora/AllGiftedPassages/gift
    _passages_all_IRR.rds")

saveRDS(keepers_vector_internal_sampled, "R_Objects/Corpora/
    AllGiftedPassages/keepers_vector_internal_sampled.rds")

saveRDS(agreements, "R_Objects/Corpora/AllGiftedPassages/agreements.rds"
    )

saveRDS(disagreements, "R_Objects/Corpora/AllGiftedPassages/
    disagreements.rds")

saveRDS(overall_agreement, "R_Objects/Corpora/AllGiftedPassages/overall_
    agreement.rds")

saveRDS(Ratings_df, "R_Objects/Corpora/AllGiftedPassages/Ratings_df.rds"
    )
```

```
saveRDS(cohen_kappa, "R_Objects/Corpora/AllGiftedPassages/cohen_kappa.rds")

saveRDS(only_passages_all, "R_Objects/Corpora/only_passages_all.rds")

saveRDS(intermediate_gift_passages51, "R_Objects/Corpora/intermediate_gift_passages51.rds")

saveRDS(intermediate_only_passages51, "R_Objects/Corpora/intermediate_only_passages51.rds")

saveRDS(combined_word_counts51, "R_Objects/Corpora/combined_word_counts51.rds")

saveRDS(intermediate_gift_passages201, "R_Objects/Corpora/intermediate_gift_passages201.rds")

saveRDS(intermediate_only_passages201, "R_Objects/Corpora/intermediate_only_passages201.rds")

saveRDS(combined_word_counts201, "R_Objects/Corpora/combined_word_counts201.rds")

# Retrieve the names of all objects in the global environment and store them in a character vector.

all_objects <- ls()

# Create a character vector of object names in the global environment.

formatted_object_array <- paste('c("', paste(all_objects, collapse = '", "'), '")', sep = "")

# Export a formatted list to a .txt file for manual review.

write(formatted_object_array, "R_objects.txt")

# Manually review the .txt file and delete objects to be kept in the R global environment.

# Import the first line of the modified .txt file back into R.
```

```
string_of_object_names <- readLines("R_objects.txt", n = 1)

# Convert the string in the .txt file back into a character vector of
    object names.

formatted_object_array <- eval(parse(text = string_of_object_names))

# Remove the objects selected for removal from the global environment.

rm(list = c(formatted_object_array))

rm(list = c("all_objects", "formatted_object_array", "string_of_object_
   names"))

# Invoke the garbage collection function gc() to release memory occupied
     by no-longer-used objects and to compact the storage of remaining
    objects in the global environment to improve memory usage.

gc()
```

7 Corpus Consolidation

7.1 Combining the Corpora

Create one corpus with the 51-word *only* text windows and the relevant 51-word *gift** text windows. The resulting corpus will provide the basis for the remaining parts of the analysis.

Load the `gift_passages_all` and `only_passages_all` objects from the RDS files stored outside of R back into the global environment (as needed).

Listing 7.1: Loading the Requisite Objects for Corpus Consolidation

```
gift_passages_all <- readRDS("R_Objects/Corpora/gift_passages_all.rds")

only_passages_all <- readRDS("R_Objects/Corpora/only_passages_all.rds")
```

Now combine the relevant parts of both corpora.

Listing 7.2: Consolidating the Text Windows

```
gift_passages_all_relevant <- gift_passages_all %>%

# Subset the gift_passages_all data frame to include only the passages
    with relevant meanings of 'gift*' (i.e., gift_passages_all$Keepers =
    "yes").

  filter(Keepers == "yes")

str(gift_passages_all_relevant)

# Remove the now unneeded Keepers variable.

gift_passages_all_relevant <- gift_passages_all_relevant %>%

  select(-Keepers)

# Add a factor variable to both data frames for 'gifted'/'only'.

gift_passages_all_relevant <- gift_passages_all_relevant %>%

  mutate(SampleType = factor("gift", levels = c("gift", "only")))

only_passages_all <- only_passages_all %>%

  mutate(SampleType = factor("only", levels = c("gift", "only")))

# Combine the two data frames into one, and make sure the columns match.

names(gift_passages_all_relevant)

names(only_passages_all)

# Use the bind_rows() function to combine the data frames.

gift_only_passages <- bind_rows(gift_passages_all_relevant, only_
    passages_all)
```

```
class(gift_only_passages)

names(gift_only_passages)

str(gift_only_passages$SampleType == "only")

cat(gift_only_passages[[147781, 3]])

# Add a new column with the entire 51-word passages.

gift_only_passages$Passages51 <- paste0(gift_only_passages$Pre25, "␣",

  gift_only_passages$Node_word1, "␣",

  gift_only_passages$Post25)

# Convert the Passages51 column to a Quanteda corpus object.

gift_only_corpus51 <- corpus(gift_only_passages$Passages51)

# Add the 'Year', 'SampleType', and 'Annual_Count' columns as Quanteda-style 'docvars' (i.e., document variables).

docvars(gift_only_corpus51, "Year") <- gift_only_passages$Year

docvars(gift_only_corpus51, "SampleType") <- gift_only_passages$SampleType

docvars(gift_only_corpus51, "Annual_Count") <- gift_only_passages$Annual_Count

str(gift_only_corpus51)
```

7.2 Removing Left-Over HTML Markup, Replacing Contractions, and Standardizing Irregular Instances of *gift** and *only*

First, review the *gift** passages and *only* passages in the `gift_only_corpus51` object to find (a) left-over HTML markup, (b) contractions, and (c) hyphenated or compound words connected to 'gift' or 'only' that should be separated into separate strings.

To this end, create a mapping of all such cases and their replacements.

The identification of (a) left-over HTML markup and (b) contractions requires a manual review of a sample of cases and is done first, in the following steps. The identification of (c) hyphenated or compound words connected to 'gift' or 'only' that should be separated into separate strings will be done in R, as described below.

Start by exporting all of the *gift** passages and 1 percent of the *only* passages (evenly distributed over all such samples) from the `gift_only_corpus51` object for review in Excel.

Listing 7.3: Exporting All *gift** Passages and 1 Percent of *only* Passages

```
# Extract the document variables.

docvars_df <- docvars(gift_only_corpus51)

# Extract the text data as a character vector.

text_data_gift_only <- as.character(gift_only_corpus51)

# Extract the document names.

docnames_gift_only <- docnames(gift_only_corpus51)

# Filter texts with SampleType = "gift".

gift_indices <- which(docvars_df$SampleType == "gift")

gift_texts_all <- text_data_gift_only[gift_indices]

gift_docnames_all <- docnames_gift_only[gift_indices]

# Find the indices for the "only" texts.

only_indices <- which(docvars_df$SampleType == "only")

# Count the total number of texts with SampleType = "only".

total_only_texts <- length(only_indices)

# Calculate the sampling interval for one percent of the 'only' texts
```

```
    and round it to the closest integer.

sample_size <- ceiling(0.01 * total_only_texts)

sampling_interval <- max(1, round(total_only_texts / sample_size))

# Generate a resulting sequence of indices for the 'only' sample.

sampled_only_indices <- seq(1, total_only_texts, by = sampling_interval)

# Check for out-of-bounds values.

out_of_bounds <- sampled_only_indices > total_only_texts

print(out_of_bounds)

# Adjust the indices to get the actual positions in the corpus.

actual_sampled_only_indices <- only_indices[sampled_only_indices]

# Sample texts and document names using the calculated indices within
    the only_indices.

sampled_only_texts <- text_data_gift_only[actual_sampled_only_indices]

sampled_only_docnames <- docnames_gift_only[actual_sampled_only_indices]

# Combine the two sets of texts and their original document names.

combined_texts <- c(gift_texts_all, sampled_only_texts)

combined_docnames <- c(gift_docnames_all, sampled_only_docnames)

# Create a data frame for exporting to Excel for the manual review.

output_df_for_manual_review <- data.frame(OriginalDocName = combined_
    docnames, Text = combined_texts, stringsAsFactors = FALSE)

# Review the final data frame structure before exporting.

str(output_df_for_manual_review)

```

```
# Export the data frame to an Excel file.

write.xlsx(output_df_for_manual_review, file = "R_Objects/Corpora/ManualReview/sampled_texts_for_manual_review.xlsx", rowNames = FALSE)
```

Now read through the 3,570 sampled text passages in the resulting Excel file and record (a) instances of left-over HTML markup and (b) contractions (i.e., contracted or elided forms such as "they ' ve"). Create a replacement mapping that replaces all instances of left-over HTML markup with " " and each specific contraction with one possible full form (e. g. replace "they ' ve" with "they have"). Due to the stop-word procedure to be implemented in a later step (see Listing 8.2), it does not matter which full form is used for a given contraction, as all common grammar words, including the full forms of contractions, will be subsequently removed.

We created a separate Excel file called `Replacement Mapping.xlsx` to keep track of required replacements during our manual review of `sampled_texts_for_manual_review.xlsx`. In `Replacement Mapping.xlsx`, Column A is called "Find", and Column B is called "Replace With." After completing the mapping, we exported the strings to be replaced to a text file called `patterns_to_replace.txt` and the corresponding replacements to a text file called `replacements.txt`.

Then, back in R, review all unique values for 'gift*' and 'only' to find strings that reflect hyphenated or compound words connected to 'gift' or 'only' that should be separated into separate strings. Add these forms to the existing replacement mapping (made outside of R).

Extract and review the unique strings containing *gift**.

Listing 7.4: Reviewing Unique Strings Starting With *gift**

```
# Use a regular expression to find all unique 'gift*' strings within these texts.

gift_strings <- unlist(regmatches(gift_texts_all, gregexpr("\\bgift[a-zA-Z-]*[^a-zA-Z0-9]*\\b", gift_texts_all, ignore.case = TRUE)))

unique_gift_strings <- unique(gift_strings)

# Print the unique 'gift*' strings for review. Then review these.

```

```
print(unique_gift_strings)
```

The following strings are hyphenated or compound words connected to ‘gift’ that should be separated into separate strings: “gifted-and-talented”, “gift- ”, “gift-a”, “gift--an”, “gift-as”, “gifted-or”, “gifted-well”, “gift-in”, and “giftthe”.

Add the following replacements to the existing replacement mapping (outside or R) to account for these cases:

- “gifted-and-talented” → “gifted and talented”
- “gift-” → “gift ”
- “giftthe” → “gift the”

Extract and review the unique strings containing *only*.

Listing 7.5: Reviewing Unique Strings Starting With *only*

```
# Use a regular expression to find all unique 'only' strings within
    these texts.

# Create an object with the 'only' texts.

# Filter texts with SampleType = "only".

only_texts_all <- text_data_gift_only[only_indices]

# Extract the unique strings containing 'only'.

only_strings <- unlist(regmatches(only_texts_all, gregexpr("\\bonly[a-zA
    -Z-]*[^a-zA-Z0-9]*\\b", only_texts_all, ignore.case = TRUE)))

unique_only_strings <- unique(only_strings)

# Print the unique 'only' strings for review.

print(unique_only_strings)
```

The following strings are hyphenated or compound words connected to ‘only’ that should be separated into separate strings: “only-child”, “only-in-Newark”, “only-knows”, “only-too-wise”, “only- ” “Only-if”, “only-She”, “onlysuitable ”, and “only.”.

Add the following replacements to the existing replacement mapping (outside or R) to account for these cases:

- “only-in-Newark” → “only in Newark ”
- “only-too-wise” → “only too wise ”
- “only-” → “only ”
- “Only-” → “Only ”
- “onlysuitable” → “only suitable”
- “only.” → “only ”

Now apply the resulting replacement mapping to the `gift_only_corpus51` object.

Listing 7.6: Applying the Resulting Replacement Mapping

```
# Load into the R environment the mapping of patterns to be replaced and the replacements.

# Load the patterns to replace.

patterns_to_replace <- readLines("patterns_to_replace.txt")

# Load the replacements.

replacements <- readLines("replacements.txt")

# Check whether the lengths of both vectors match. If the lengths do not match, fix the problem and load the revised mappings.

if(length(patterns_to_replace) != length(replacements)) {stop("The␣lengths␣of␣patterns_to_replace␣and␣replacements␣do␣not␣match.")}

# Create a tibble for human inspection and double-check that each to-be-replaced string maps onto the replacement string is as intended.
```

```
replacement_mapping <- tibble(
  patterns_to_replace = patterns_to_replace,
  replacements = replacements
)
# Print the tibble for inspection.
print(replacement_mapping, n = Inf)
# Verify that the to-be-replaced patterns are being found in the corpus.
# Extract the text from the corpus.
text_vector <- as.character(gift_only_corpus51)
# Check for occurrences of each pattern in the corpus texts.
occurrences <- sapply(patterns_to_replace, function(pattern) {
  any(grepl(pattern, text_vector, fixed = TRUE))
})
# Create a tibble for checking occurrences.
occurrences_tibble <- tibble(
  patterns_to_replace = patterns_to_replace,
  occurs_in_corpus = occurrences
)
# Print the occurrences tibble for review.
print(occurrences_tibble, n = Inf)
# Check whether all patterns occur at least once in the corpus.
```

```r

if(!all(occurrences)) {

  warning("Not␣all␣patterns␣to␣replace␣occur␣in␣the␣corpus.")

}

# Perform the replacements and return a tibble with a summary of replacements.

# Write a function to perform the replacements and count the number of replacements.

replace_patterns <- function(text, patterns, replacements) {

  replacement_counts <- integer(length(patterns))

  for (i in seq_along(patterns)) {

    pattern <- patterns[i]

    replacement <- replacements[i]

    replacement_counts[i] <- str_count(text, fixed(pattern))

    text <- gsub(pattern, replacement, text, fixed = TRUE)

  }

  return(list(text = text, counts = replacement_counts))

}

# Apply the replacement function to each document in the corpus and collect counts.

replacement_results <- lapply(text_vector, replace_patterns, patterns = patterns_to_replace,replacements = replacements)

# Extract the replaced text and counts.

text_vector_replaced <- sapply(replacement_results, `[[`, "text")
```

```
replacement_counts <- do.call(rbind, lapply(replacement_results, `[[`, "
    counts"))

# Sum the counts for each pattern across all documents.

total_replacement_counts <- colSums(replacement_counts)

# Create a tibble for reviewing the counts.

replacement_count_summary <- tibble(

  patterns = patterns_to_replace,

  replacements = replacements,

  total_replacements = total_replacement_counts

)

# Print the summary of replacement counts and inspect them to make sure
    all replacements worked as intended in the replacement mapping.

print(replacement_count_summary, n = Inf)
```

Update the corpus with the replaced text and review the result.

Listing 7.7: Updating the Corpus

```
# Verify that the names in 'text_vector_replaced' match the document
    names in the original corpus.

original_docnames <- docnames(gift_only_corpus51)

updated_docnames <- names(text_vector_replaced)

if (!all(original_docnames == updated_docnames)) {
```

```
  stop("Warning: Document names do not match between the original corpus and the revised text vector!")}

# Create a new corpus with the replaced texts and same metadata.

gift_only_corpus51b <- corpus(text_vector_replaced, docvars = docvars(gift_only_corpus51))

# Update document names to match original corpus.

docnames(gift_only_corpus51b) <- original_docnames

# Verify the update by checking a few documents.

cat("Original corpus, first five texts:\n")

print(as.character(gift_only_corpus51)[1:5])

cat("\nReplaced text vector, first five texts:\n")

print(text_vector_replaced[1:5])

cat("\nUpdated corpus, first five texts:\n")

print(as.character(gift_only_corpus51b)[1:5])

# Compare the original and updated versions of the corpus to make sure the substitutions worked as intended.

# Count and compare the number of documents in the original and updated corpus.

num_docs_original <- ndoc(gift_only_corpus51)

num_docs_updated <- ndoc(gift_only_corpus51b)

cat("Number of documents:\n")

cat("Original corpus: ", num_docs_original, "\n")

cat("Updated corpus: ", num_docs_updated, "\n")

```

```
# Count and compare the total number of characters in the original and updated corpus.

total_chars_original <- sum(nchar(as.character(gift_only_corpus51)))

total_chars_updated <- sum(nchar(as.character(gift_only_corpus51b)))

cat("\nTotal␣number␣of␣characters:\n")

cat("Original␣corpus:␣", total_chars_original, "\n")

cat("Updated␣corpus:␣", total_chars_updated, "\n")

# Count and compare the lengths of individual documents.

lengths_original <- nchar(as.character(gift_only_corpus51))

lengths_updated <- nchar(as.character(gift_only_corpus51b))

cat("\nComparison␣of␣individual␣document␣lengths:\n")

summary(lengths_original)

summary(lengths_updated)
```

Clean up the global environment.

Listing 7.8: Cleaning up the Global Environment After Corpus Consolidation

```
# Save the objects needed for later steps and/or for auditing our work.

saveRDS(gift_passages_all_relevant, "R_Objects/Corpora/gift_passages_all_relevant.rds")

saveRDS(gift_only_passages, "R_Objects/Corpora/gift_only_passages.rds")

saveRDS(gift_only_corpus51, "R_Objects/Corpora/gift_only_corpus51.rds")
```

```
saveRDS(gift_indices, "R_Objects/Corpora/gift_indices.rds")

saveRDS(only_indices, "R_Objects/Corpora/only_indices.rds")

saveRDS(total_only_texts, "R_Objects/Corpora/total_only_texts.rds")

saveRDS(actual_sampled_only_indices, "R_Objects/Corpora/actual_sampled_
    only_indices.rds")

saveRDS(combined_texts, "R_Objects/Corpora/combined_texts.rds")

saveRDS(combined_docnames, "R_Objects/Corpora/combined_docnames.rds")

saveRDS(output_df_for_manual_review, "R_Objects/Corpora/output_df_for_
    manual_review.rds")

saveRDS(unique_gift_strings, "R_Objects/Corpora/unique_gift_strings.rds"
    )

saveRDS(unique_only_strings, "R_Objects/Corpora/unique_only_strings.rds"
    )

saveRDS(patterns_to_replace, "R_Objects/Corpora/patterns_to_replace.rds"
    )

saveRDS(replacements, "R_Objects/Corpora/replacements.rds")

saveRDS(replacement_mapping, "R_Objects/Corpora/replacement_mapping.rds"
    )

saveRDS(occurrences_tibble, "R_Objects/Corpora/occurrences_tibble.rds")

saveRDS(replacement_results, "R_Objects/Corpora/replacement_results.rds"
    )

saveRDS(text_vector_replaced, "R_Objects/Corpora/text_vector_replaced.
    rds")

saveRDS(replacement_counts, "R_Objects/Corpora/replacement_counts.rds")

saveRDS(replacement_count_summary, "R_Objects/Corpora/replacement_count_
```

```r
summary.rds")

saveRDS(gift_only_corpus51b, "R_Objects/Corpora/gift_only_corpus51b.rds")

# Retrieve the names of all objects in the global environment and store them in a character vector.

all_objects <- ls()

# Create a character vector of object names in the global environment.

formatted_object_array <- paste('c("', paste(all_objects, collapse = '", "'), '")', sep = "")

# Export a formatted list to a .txt file for manual review.

write(formatted_object_array, "R_objects.txt")

# Manually review the .txt file and delete objects to be kept in the R global environment.

# Import the first line of the modified .txt file back into R.

string_of_object_names <- readLines("R_objects.txt", n = 1)

# Convert the string in the .txt file back into a character vector of object names.

formatted_object_array <- eval(parse(text = string_of_object_names))

# Remove the objects selected for removal from the global environment.

rm(list = c(formatted_object_array))

rm(list = c("all_objects", "formatted_object_array", "string_of_object_names"))

# Invoke the garbage collection function gc() to release memory occupied by no-longer-used objects and to compact the storage of remaining objects in the global environment to improve memory usage.

```

```
gc()
```

8 Corpus Preprocessing

Load the `gift_only_corpus51` object from the RDS file stored outside of R back into the global environment (as needed).

Listing 8.1: Loading the Requisite Object for Preprocessing

```
gift_only_corpus51b <- readRDS("R_Objects/Corpora/gift_only_corpus51b.
   rds")
```

Clean, tokenize, and lemmatize the *gift** and *only* text windows and describe the resulting corpora.

First, tokenize the corpora. Also remove the node words *gift** and *only*.

For the functions used and options selected in Listing 8.2, see the documentation of Quanteda version 3.3.1 [8]. For the SMART Stop Word List used therein, see [29] and [20]. As the SMART Stop Word List contains the word *only*, the removal of that node word does not require a separate call.

Listing 8.2: Tokenizing and Preprocessing

```
gift_only_tokens51 <-

  tokens(

    gift_only_corpus51b,

    remove_punct = TRUE,

    remove_symbols = TRUE,
```

```
    remove_numbers = TRUE,
    remove_url = TRUE,
    remove_separators = TRUE,
    split_hyphens = TRUE,
    split_tags = FALSE,
    include_docvars = TRUE,
    padding = FALSE,
    verbose = TRUE) %>%
  tokens_tolower(keep_acronyms = FALSE) %>%
    tokens_remove(pattern = stopwords::stopwords("en", source = "smart")
      ) %>%
  tokens_remove(pattern = "^gift.*", valuetype = "regex") %>%
  tokens_remove(pattern = "-", valuetype = "fixed") %>%
  tokens_remove(pattern = "-", valuetype = "fixed")
```

Third, lemmatize the *gift** and *only* passages with the Python spaCy package via the reticulate package [32], a Python wrapper for R, and the spacyr package [5], a spaCy wrapper for R.

Start this process by installing Python on your device. During installation, select the option to add Python to the so-called “PATH.” This makes Python executable from the terminal, which will be required when running Python via R. The spacyr and reticulate packages were installed, but not loaded above in Section 4. To work properly with Python and the Python spaCy package, they must be loaded in the sequence described below.

Now configure Python to work with the reticulate package.

Listing 8.3: Configuring Python and Getting the Path for Python Executable File

```
# Make sure Python is set up on your device and that reticulate can find
    it.

reticulate::py_config()

# Locate the virtual instantiation of Python set up on your device by
    the reticulate package. On our device, it was located at 'C:\Users\
    LocalAdmin\Documents\.virtualenvs\r-reticulate\Scripts'. The
    directory must contain the 'python.exe' file.
```

Now open the command prompt on your computer and install spaCy from the command prompt using the pip command in Python (used for installing and managing Python packages), using the Python path identified for your device in Listing 8.3. Specification of this directory may not be necessary if Python has already been added to your PATH. For our device, for example, the command read as follows:

Listing 8.4: Installing spaCy from the Command Prompt

```
# C:/Users/LocalAdmin/Documents/.virtualenvs/r-reticulate/Scripts/python
    .exe -m pip install spacy
```

Still in the terminal, download the English language model. This, too, will depend on the exact path of the Python executable file found above. For our device, for example, the command read as follows:

Listing 8.5: Downloading the English Language Model in Python

```
# C:/Users/LocalAdmin/Documents/.virtualenvs/r-reticulate/Scripts/python
    .exe -m spacy download en_core_web_sm
```

Back in R, load the reticulate package and attach it to `python.exe` within the virtual instantiation of Python located in the aforementioned directory (i. e., its analog on your device).

Listing 8.6: Loading and Attaching the Reticulate Package

```
library(reticulate)

use_python("C:/Users/LocalAdmin/Documents/.virtualenvs/r-spacyr/Scripts/
    python.exe", required = TRUE)
```

Then load the spacyr package within R.

Listing 8.7: Loading the Spacyr Package Within R

```
library(spacyr)
```

Test the spaCy installation in R.

Listing 8.8: Testing the spaCy Installation in R

```
py_run_string("import␣spacy")
```

Now install and load spaCy and the English language model in R.

Listing 8.9: Loading the English Language Model in R

```
spacy_initialize(model = "en_core_web_sm")
```

Make sure you are using the Python spaCy version we used, which was Version 3.7.2.

Listing 8.10: Checking Python SpaCy Version

```
py_run_string("import␣spacy;␣print(spacy.__version__)")
end{myrcode}

If you are using a newer Python spaCy version, revert to Version 3.7.2.

Now insert the following Python function definition:

\begin{myrcode}[label={lst:Inserting Python Function Definition}]{Inserting Python Function Definition}
py_run_string("

def␣custom_lemmatizer(tokens):

␣␣corrected_lemmas␣=␣[]

␣␣for␣token␣in␣tokens:

␣␣␣␣lemma␣=␣token['lemma']

␣␣␣␣if␣lemma␣==␣'talente':

␣␣␣␣␣␣corrected_lemmas.append('talent')

␣␣␣␣else:

␣␣␣␣␣␣corrected_lemmas.append(lemma)

```

```
␣␣return␣corrected_lemmas

")
```

The function definition will be needed for a custom function later on, which is required to emend an inconsistency in the lemmatization (explained below).

Now verify that the spaCy lemmatization plug-in is working in R.

Listing 8.11: Verifying the SpaCy Lemmatization Plug-In

```
# Define a test sentence for testing the spaCy lemmatization plug-in.

test_sentence <- "Understanding␣that␣Greg␣understands␣us␣helps␣me,␣as␣a␣talented␣helper,␣who's␣often␣been␣helped␣in␣developing␣her␣talents␣in␣the␣past,␣to␣understand␣what␣you␣understood␣when␣Greg␣stood␣in␣the␣room␣and␣moved␣the␣music␣stand␣to␣Greg's␣side␣-␣his␣side␣-␣of␣the␣room."

# Use the spacy_parse() function to lemmatize the sentence.

parsed_text <- spacy_parse(test_sentence)

# Show lemmatized words and review them to make sure the lemmatization is working.

print("Lemmatized␣Words:")

print(parsed_text$lemma)
```

Once assured that the spaCy lemmatization plug-in is working properly, convert the `gift_only_tokens51` object back into a corpus object and thence into a data frame in preparation for lemmatization.

Listing 8.12: Converting the `gift_only_tokens51` Object Back into a Corpus Object

```
gift_only_corpus_downstream51 <- vapply(gift_only_tokens51, paste, FUN.
    VALUE = character(1), collapse = "␣")

class(gift_only_corpus_downstream51)

str(gift_only_corpus_downstream51)

head(gift_only_corpus_downstream51)

# Convert gift_only_corpus_downstream51 to a data frame.

gift_only_corpus_downstream_df51 <- data.frame(text = gift_only_corpus_
    downstream51, stringsAsFactors = FALSE)

View(gift_only_corpus_downstream_df51)

names(gift_only_corpus_downstream_df51)
```

Our conversion of the `gift_only_tokens51` object back into a corpus object followed the solution suggested by [1].

Use the `spacy_parse()` function to lemmatize the texts.

Listing 8.13: Lemmatizing Our Texts

```
lemmatized_gift_only_texts51 <- spacy_parse(gift_only_corpus_downstream_
    df51$text, lemma = TRUE, entity = FALSE, pos = TRUE, sentence_id =
    FALSE)

class(lemmatized_gift_only_texts51)

names(lemmatized_gift_only_texts51)

```

```
lemmatized_gift_only_texts51[1:31,]

View(lemmatized_gift_only_texts51)

dim(lemmatized_gift_only_texts51)

str(lemmatized_gift_only_texts51)

head(lemmatized_gift_only_texts51)
```

Due to an irregularity in the lemmatization of 'talented,' which we noticed at a later point in this workflow, the token 'talented' was variously lemmatized to 'talented' or to 'talente,' depending on the part of speech (pos), which varies, as illustrated with the following call. Similarly, there is a one-time inconsistency in the lemmatization of 'talents' to 'talents' (rather than to 'talent') due to a different part-of-speech assessment (i. e., 'PROPN' rather than the usual 'NOUN'). The token 'talent' is not affected; it is consistently lemmatized as 'talent,' despite a number of part-of-speech assessments returning as 'PROPN' rather than 'NOUN.' The following listing illustrates the inconsistencies.

Listing 8.14: Reviewing Lemmatization Inconsistencies

```
filtered_data <- lemmatized_gift_only_texts51 %>%

  filter(token == "talented")

print(filtered_data)

filtered_data2 <- lemmatized_gift_only_texts51 %>%

  filter(token == "talents")

print(filtered_data2)

filtered_data3 <- lemmatized_gift_only_texts51 %>%

  filter(token == "talent")

print(filtered_data3)
```

Consolidate the lemmata provided for the tokens ‘talent,’ ‘talents,’ and ‘talented’ as ‘talent’ and the various parts of speech as ‘NOUN.’

Listing 8.15: Consolidating Lemmata

```
lemmatized_gift_only_texts51_corrected <- lemmatized_gift_only_texts51 %>%
  mutate(
    # Consolidate the lemmata 'talented', 'talent', and 'talents' as 'talent' for the tokens 'talented', 'talent', and 'talent'.
    lemma = case_when(
      token %in% c("talented", "talent", "talents") ~ "talent",
      lemma %in% c("talent", "talented", "talente", "talents") ~ "talent",
      TRUE ~ lemma
    ),
    # Consolidate the various part-of-speech (pos) values as 'NOUN' for all (new) lemma values 'talent'.
    pos = case_when(
      lemma == "talent" ~ "NOUN",
      TRUE ~ pos
    )
  )
filtered_data4 <- lemmatized_gift_only_texts51_corrected %>%
  filter(lemma == "talent")
```

Convert the `spacyr_parsed` **`data.frame`** back to a Quanteda tokens object.

Listing 8.16: Converting the SpaCy Object back to Quanteda

```
# Aggregate the lemmata based on doc_id and convert them back to a
    Quanteda tokens object.

gift_only_tokens_lemmatized51 <- as.tokens(lemmatized_gift_only_texts51_
    corrected, use_lemma = TRUE)

# Check the result against the tokenized texts in gift_only_tokens51.

gift_only_tokens51[[1]]

gift_only_tokens_lemmatized51[[1]]

gift_only_tokens51[[2]]

gift_only_tokens_lemmatized51[[2]]

gift_only_tokens51[[3]]

gift_only_tokens_lemmatized51[[3]]

gift_only_tokens51[[4]]

gift_only_tokens_lemmatized51[[4]]

gift_only_tokens51[[5]]

gift_only_tokens_lemmatized51[[5]]

gift_only_tokens51[[67]]

gift_only_tokens_lemmatized51[[67]]

# Append the original document variables (docvars) to the new tokens
    object. Extract the docvars from gift_only_tokens51.

original_docvars51 <- docvars(gift_only_tokens51)
```

```

# Assign the extracted docvars to gift_only_tokens_lemmatized51.

docvars(gift_only_tokens_lemmatized51) <- original_docvars51

str(gift_only_tokens_lemmatized51)
```

Inspect the lemmatized tokens object, and then describe the *gift** and *only* passages therein.

Listing 8.17: Inspecting the Lemmatized Tokens Object

```
number_of_lines51 <- length(gift_only_tokens_lemmatized51)

print(number_of_lines51)

docvar_data51 <- docvars(gift_only_tokens_lemmatized51)

number_of_gift_lines51 <- sum(docvar_data51$SampleType == "gift")

print(number_of_gift_lines51)

number_of_only_lines51 <- sum(docvar_data51$SampleType == "only")

print(number_of_only_lines51)

# Check the work.

number_of_lines51 - number_of_gift_lines51 - number_of_only_lines51

# The result should equal 0.

# Check whether, after preprocessing and tokenization (above), any of
    the documents now have 0 tokens. If yes, these should be removed.

token_counts_per_document51 <- sapply(gift_only_tokens_lemmatized51,
    length)

```

```r
gift_only_tokens_lemmatized51b <- gift_only_tokens_lemmatized51[token_
    counts_per_document51 != 0]

token_counts_per_document51b <- sapply(gift_only_tokens_lemmatized51b,
    length)

length(token_counts_per_document51) - length(token_counts_per_
    document51b)

# The result equals 0. Hence, no document has 0 tokens. Hence, no
    documents need to be removed.

# Spot check the resulting tokens to ensure they are as expected.

print(gift_only_tokens_lemmatized51[1:3], max_ntoken = 51)
```

Now get descriptive statistics for the *gift** and *only* documents.

Listing 8.18: Describing the *gift** and *only* Documents in the Lemmatized Tokens Object

```r
# Access the docvars from the tokens object.

docvar_data51 <- docvars(gift_only_tokens_lemmatized51)

# Prepare count variables.

count_gift51 <- 0

count_only51 <- 0

count_list_gift51 <- c()

count_list_only51 <- c()

count_list_total51 <- c()

# Iterate over the tokens list to count tokens per text and classify the
```

```
    counts based on sample type.
for(i in 1:length(gift_only_tokens_lemmatized51)) {
  num_tokens51 <- length(gift_only_tokens_lemmatized51[[i]])
  count_list_total51 <- c(count_list_total51, num_tokens51)
  if(docvar_data51$SampleType[i] == "gift") {
    count_gift51 <- count_gift51 + num_tokens51
    count_list_gift51 <- c(count_list_gift51, num_tokens51)
  } else if(docvar_data51$SampleType[i] == "only") {
    count_only51 <- count_only51 + num_tokens51
    count_list_only51 <- c(count_list_only51, num_tokens51)
  }
}
# Calculate the mean and the standard deviation (SD).
mean_gift51 <- mean(count_list_gift51)
print(mean_gift51)
sd_gift51 <- sd(count_list_gift51)
print(sd_gift51)
mean_only51 <- mean(count_list_only51)
print(mean_only51)
sd_only51 <- sd(count_list_only51)
print(sd_only51)
```

```r
mean_total51 <- mean(count_list_total51)

print(mean_total51)

sd_total51 <- sd(count_list_total51)

print(sd_total51)
```

Clean up the global environment.

Listing 8.19: Cleaning up the Global Environment After Corpus Preprocessing

```r
# Save the objects needed for later steps and/or for auditing our work.

saveRDS(gift_only_tokens51, "R_Objects/Corpora/gift_only_tokens51.rds")

saveRDS(test_sentence, "R_Objects/Corpora/test_sentence.rds")

saveRDS(parsed_text, "R_Objects/Corpora/parsed_text.rds")

saveRDS(gift_only_tokens_lemmatized51, "R_Objects/Corpora/gift_only_tokens_lemmatized51.rds")

saveRDS(number_of_lines51, "R_Objects/Corpora/number_of_lines51.rds")

saveRDS(number_of_gift_lines51, "R_Objects/Corpora/number_of_gift_lines51.rds")

saveRDS(number_of_only_lines51, "R_Objects/Corpora/number_of_only_lines51.rds")

saveRDS(count_list_gift51, "R_Objects/Corpora/count_list_gift51.rds")

saveRDS(count_list_only51, "R_Objects/Corpora/count_list_only51.rds")

saveRDS(count_list_total51, "R_Objects/Corpora/count_list_total51.rds")
```

```r
# Retrieve the names of all objects in the global environment and store them in a character vector.
all_objects <- ls()
# Create a character vector of object names in the global environment.
formatted_object_array <- paste('c("', paste(all_objects, collapse = '", "'), '")', sep = "")
# Export a formatted list to a .txt file for manual review.
write(formatted_object_array, "R_objects.txt")
# Manually review the .txt file and delete objects to be kept in the R global environment.
# Import the first line of the modified .txt file back into R.
string_of_object_names <- readLines("R_objects.txt", n = 1)
# Convert the string in the .txt file back into a character vector of object names.
formatted_object_array <- eval(parse(text = string_of_object_names))
# Remove the objects selected for removal from the global environment.
rm(list = c(formatted_object_array))
rm(list = c("all_objects", "formatted_object_array", "string_of_object_names"))
# Invoke the garbage collection function gc() to release memory occupied by no-longer-used objects and to compact the storage of remaining objects in the global environment to improve memory usage.
gc()
```

9 Keyword Identification

Load the lemmatized tokens object `gift_only_tokens_lemmatized51` back into the global environment (as needed) and create a document–feature matrix with rows by sample type using the Quanteda `dfm()` function.

Listing 9.1: Preparing for the Keyword Identification

```
gift_only_tokens_lemmatized51 <- readRDS("R_Objects/Corpora/gift_only_
    tokens_lemmatized51.rds")

str(gift_only_tokens_lemmatized51)

gift_only_dfm51 <- dfm(gift_only_tokens_lemmatized51) %>%

  dfm_group(groups = SampleType)

str(gift_only_dfm51)

dim(gift_only_dfm51)

gift_only_dfm51[, 1:10]
```

Use the `textstat_keyness()` function [9] to implement a keyness analysis to identify keywords in the *gift** samples (i. e., the target corpus) in comparison to the *only* samples (i. e., the reference corpus).

Listing 9.2: Implementing a Keyness Analysis

```
gift_only_keywords51 <- textstat_keyness(gift_only_dfm51, target = "gift
    ", measure = "lr")

# Add p values corrected for the false-discovery rate (FDR).

gift_only_keywords51$p_fdr <- p.adjust(gift_only_keywords51$p, method =
    "fdr")
```

```

# Remove all keywords in which there are fewer than 10 tokens in the
    target corpus and the reference corpus.

gift_only_keywords51_filtered <- filter(gift_only_keywords51, n_target >
     9, n_reference > 9)

# Filter out all keywords that do not have significant FDR p values and
    do not have a positive G^2 statistic.

gift_only_keywords51_significant <- filter(gift_only_keywords51_filtered
    , G2 > 0, p_fdr < 0.05)

head(gift_only_keywords51_significant)

View(gift_only_keywords51_significant)
```

Add normalized versions of both count variables for comparing the occurrences of word types between the two corpora of differing size.

Listing 9.3: Normalizing Count Variables by Corpus

```
# Ascertain the total number of word tokens in the 'gift*' and 'only'
    corpora by summing the word tokens by document and sample type. Start
     by calculating the word-token counts for each document.

token_counts <- sapply(gift_only_tokens_lemmatized51, length)

# Create a new column in the list object 'doc_vars' for the token counts
    .

doc_vars <- docvars(gift_only_tokens_lemmatized51)

doc_vars$token_count <- token_counts

# Summarize the word-token counts by sample type.

```

```
corpus_sizes <- doc_vars %>%
  group_by(SampleType) %>%
  summarize(total_tokens = sum(token_count))
# Review the sizes of both corpora to select a normalization level.
print(corpus_sizes)
# Normalize n_target and n_reference at per 100,000 word tokens and add the resulting vectors to the gift_only_keywords51_significant object. Calculate normalization factors for each corpus.
normalization_factor_gift <- 100000 / corpus_sizes$total_tokens[corpus_sizes$SampleType == 'gift']
normalization_factor_only <- 100000 / corpus_sizes$total_tokens[corpus_sizes$SampleType == 'only']
# Normalize n_target and n_reference.
gift_only_keywords51_significant <- gift_only_keywords51_significant %>%
  mutate(
    n_target_norm = n_target * normalization_factor_gift,
    n_reference_norm = n_reference * normalization_factor_only
  )
# Round the normalized values to the nearest whole number.
gift_only_keywords51_significant <- gift_only_keywords51_significant %>%
  mutate(
    n_target_norm = round(n_target_norm),
    n_reference_norm = round(n_reference_norm)
```

```
  )

# Display the head of the updated data frame to confirm the changes.

head(gift_only_keywords51_significant)

# Move p_fdr two positions to the left so it follows the p-values vector
   .

gift_only_keywords51_significant <- gift_only_keywords51_significant %>%

  relocate(p_fdr, .after = p)

head(gift_only_keywords51_significant)
```

Add a column with the false-discovery-rate-corrected *p* values expressed as a log10 transformation.

Listing 9.4: Adding a Log10 Transformation

```
gift_only_keywords51_significant <- gift_only_keywords51_significant %>%

  mutate(log10_p_fdr = log10(p_fdr))

# Move log10_p_fdr four positions to the left so it follows the p_fdr
   vector.

gift_only_keywords51_significant <- gift_only_keywords51_significant %>%

  relocate(log10_p_fdr, .after = p_fdr)

head(gift_only_keywords51_significant)
```

Export the final data frame to Excel, using the openxlsx package [28] to allow for pre-defining the format of the Excel columns.

Listing 9.5: Exporting Keyword Information to Excel

```
# Create a new workbook for the impending Excel export.

wbkeywords <- createWorkbook()

# Add a worksheet to the workbook.

addWorksheet(wbkeywords, "Sheet1")

# Create custom column format styles for the Excel export via openxlsx.

header_style <- createStyle(fontName = "Calibri", fontColour = "black",
    textDecoration = "bold")

char_style <- createStyle(numFmt = "@") # This is for text data.

numeric_with_decimals_style <- createStyle(numFmt = "
    0.0000000000000000000000") # This allows for 22 digits after the
    decimal point.

whole_numbers_style <- createStyle(numFmt = "0") # Needed for whole
    numbers.

# Export the data frame to the worksheet.

writeData(wbkeywords, "Sheet1", gift_only_keywords51_significant)

# Define the number of rows to be formatted in the worksheet.

num_rows <- nrow(gift_only_keywords51_significant)

# Apply the column format styles to specific columns.

addStyle(wbkeywords, "Sheet1", style = header_style, rows = 1, cols =
    1:9)

addStyle(wbkeywords, "Sheet1", style = char_style, rows = 2:(num_rows +
    1), cols = 1)

```

```
addStyle(wbkeywords, "Sheet1", style = numeric_with_decimals_style, rows = 2:(num_rows + 1), cols = 2)
addStyle(wbkeywords, "Sheet1", style = numeric_with_decimals_style, rows = 2:(num_rows + 1), cols = 3)
addStyle(wbkeywords, "Sheet1", style = numeric_with_decimals_style, rows = 2:(num_rows + 1), cols = 4)
addStyle(wbkeywords, "Sheet1", style = numeric_with_decimals_style, rows = 2:(num_rows + 1), cols = 5)
addStyle(wbkeywords, "Sheet1", style = whole_numbers_style, rows = 2:(num_rows + 1), cols = 6)
addStyle(wbkeywords, "Sheet1", style = whole_numbers_style, rows = 2:(num_rows + 1), cols = 7)
addStyle(wbkeywords, "Sheet1", style = whole_numbers_style, rows = 2:(num_rows + 1), cols = 8)
addStyle(wbkeywords, "Sheet1", style = whole_numbers_style, rows = 2:(num_rows + 1), cols = 9)

# Set the column widths.

setColWidths(wbkeywords, sheet = "Sheet1", cols = 1, widths = "15")
setColWidths(wbkeywords, sheet = "Sheet1", cols = 2, widths = "30")
setColWidths(wbkeywords, sheet = "Sheet1", cols = 3, widths = "30")
setColWidths(wbkeywords, sheet = "Sheet1", cols = 4, widths = "30")
setColWidths(wbkeywords, sheet = "Sheet1", cols = 5, widths = "30")
setColWidths(wbkeywords, sheet = "Sheet1", cols = 6, widths = "15")
setColWidths(wbkeywords, sheet = "Sheet1", cols = 7, widths = "15")
setColWidths(wbkeywords, sheet = "Sheet1", cols = 8, widths = "15")
```

```
setColWidths(wbkeywords, sheet = "Sheet1", cols = 9, widths = "15")

# Save the resulting Excel workbook.

saveWorkbook(wbkeywords, "R_Objects/Keywords/Gift-only_keywords.xlsx",
    overwrite = FALSE)
```

Assess the extent of semantic overlap among the 92 keywords via a comparison of their respective synonyms as included in the Wordnet database [14, 26] and visualize the keywords with overlapping synonyms with the pheatmap package [19].

Before starting, the Wordnet program [26], located here, and version 21.0.2 of the Java Development Kit from Oracle [24], located here, must be locally installed. On Windows machines, add the installation directories to the system's PATH environment variable. On Unix-based systems, perform a similar procedure by modifying the PATH environment variable to include the installation directories of both programs.

In R, prepare the R session for working with the locally saved Wordnet data. In the following listing, we use our absolute path. The path must be changed to reflect your local installation.

Listing 9.6: Preparing the R Session for Wordnet Data

```
# Load the rJava package.

library(rJava)

# Set environment variables for the WordNet and Java installations
    required by the R session.

Sys.setenv(WNHOME = "C:/Program␣Files␣(x86)/WordNet/2.1")

# Load the wordnet package.

library(wordnet)

# Initialize rJava. It was already loaded above.

.jinit()
```

```

# Set the path to the Wordnet dictionary.

setDict("C:/Program␣Files/WordNet/2.1/dict")

# Test that the R session can access the Wordnet data.

synonyms("talent", pos = "NOUN")
```

Extract the keywords from the `gift_only_keywords51_significant` object and lemmatize these. For this, the Python spaCy and reticulate packages must still be loaded and operational, as described as of Step 3 in Section 8.

Listing 9.7: Extracting and Lemmatizing Keywords

```
# If no longer loaded, reload the spacyr and reticulate packages.

library(spacyr)

library(reticulate)

# Extract the 'feature' vector from the 'gift_only_keywords51_
    significant' object, which contains the names of the keywords.

keywords <- gift_only_keywords51_significant$feature

# Write a function for using the spacy_parse() function to lemmatize the
     keywords.

lemmatize_keywords <- function(keyword_vector) {

  lemmatized_tokens <- spacy_parse(keyword_vector, lemma = TRUE, entity
      = FALSE, pos = TRUE, sentence_id = FALSE)

  return(lemmatized_tokens)

}
```

```

# Call the function to lemmatize the keywords and inspect the resulting object.

lemmatized_result <- lemmatize_keywords(keywords)

head(lemmatized_result)

str(lemmatized_result)

print(lemmatized_result)

# Extract the lemmata and parts of speech (pos).

lemmatize_keywords_result <- lemmatized_result %>%

  select(lemma, pos)
```

Review and correct the lemmatizations.

Listing 9.8: Reviewing and Correcting the Lemmatizations

```
print(lemmatize_keywords_result)

# Replace 'PROPN' with 'NOUN'.

lemmatize_keywords_result <- lemmatize_keywords_result %>%

  mutate(pos = ifelse(pos == "PROPN", "NOUN", pos))

# Emend erroneous or unlikely parts of speech

lemmatize_keywords_result <- lemmatize_keywords_result %>%

  mutate(pos = case_when(

    lemma == "surgeon" & pos == "VERB" ~ "NOUN",
```

```
      lemma == "divine" & pos == "VERB" ~ "ADJ",
      lemma == "inherit" & pos == "NOUN" ~ "VERB",
      TRUE ~ pos  # Maintains the current pos values in the other rows.
    )
)
# Review the emended object.
print(lemmatize_keywords_result)
```

Now retrieve the synonyms for the lemmatized keywords from the locally saved Wordnet data.

Listing 9.9: Retrieving the Synonyms from Wordnet

```
# Write a function for using the wordnet synonym() function serially
get_synonyms_dynamic_pos <- function(lemmatize_keywords_result) {
  synonyms_list <- Map(function(lemma, pos) {
    synonyms <- synonyms(lemma, pos = pos)
    return(synonyms)
  }, lemmatize_keywords_result$lemma, lemmatize_keywords_result$pos)
  return(synonyms_list)
}
# Call the bespoke function to retrieve the Wordnet synonyms of the
   lemmatized keywords according to keyword and part of speech (pos).
```

```
synonyms_result <- get_synonyms_dynamic_pos(lemmatize_keywords_result)

# Review the resulting object.

class(synonyms_result)

str(synonyms_result)
```

Count the number of shared synonyms among all pairs of keywords and keep track of these with a similarity matrix that records the counts.

Listing 9.10: Counting Shared Synonyms

```
# Create an empty similarity matrix using the number of keywords in the
    'lemmatize_keywords_result' object.

similarity_matrix <- matrix(0, nrow = 92, ncol = 92)

keyword_names <- keywords

rownames(similarity_matrix) <- colnames(similarity_matrix) <- keyword_
    names

# Use a similarity calculation loop that evaluates all of the pairs of
    keywords, counts the number of identical keywords recorded for any
    given pair of keywords, and populates the respective cells in the
    similarity matrix with the resulting count. The inner loop starts
    with a condition that ensures that the pair-wise comparison process
    skips comparing a given keyword with itself.

for (i in 1:92) {

  for (j in 1:92) {

    if (i != j) {

```

```
      shared_elements <- length(intersect(synonyms_result[[i]], synonyms
         _result[[j]]))

      similarity_matrix[i, j] <- shared_elements

    }

  }

}
```

Review the resulting matrix and then exclude all keywords that share no synonyms with other keywords in preparation of visualizing the extent of synonym overlap among the keywords.

Listing 9.11: Excluding Keywords Without Shared Synonyms

```
# Check the structure of the matrix.

str(similarity_matrix)

# View the top-left corner of the matrix.

similarity_matrix[1:30, 1:10]

# Get summary statistics for the entire matrix.

summary(as.vector(similarity_matrix))

# Count the number of keyword pairs with synonyms.

count_greater_than_zero <- sum(similarity_matrix > 0)

print(count_greater_than_zero)

cell_count_overall <- sum(similarity_matrix >= 0)

print(cell_count_overall)
```

```r

# Visualize the distribution of similarity scores.

hist(as.vector(similarity_matrix), main = "Distribution of Similarity Scores", xlab = "Number of Shared Elements")

# Exclude rows and columns that only contain 0s. Start by finding the indices of rows and columns where the maximum value is greater than 0.

rows_to_keep <- apply(similarity_matrix, 1, function(x) max(x) > 0)

cols_to_keep <- apply(similarity_matrix, 2, function(x) max(x) > 0)

# Subset the matrix to keep only those rows and columns that have shared synonyms.

filtered_similarity_matrix <- similarity_matrix[rows_to_keep, cols_to_keep]
```

Calculate the modal value for synonym counts among all keyword pairs with one or more synonyms.

Listing 9.12: Calculating the Modal Value for Synonym Overlaps

```r
# Flatten the matrix into a vector and keep only non-zero values.

non_zero_values <- as.vector(filtered_similarity_matrix)

non_zero_values <- non_zero_values[non_zero_values > 0]

# Create a frequency table and find the modal value.

value_counts <- table(non_zero_values)

modal_value <- as.numeric(names(value_counts)[which.max(value_counts)])

# Print the modal value.
```

```
print(modal_value)
```

Plot a heatmap of the remaining keywords using the already loaded pheatmap package [19] and export the resulting plot.

Listing 9.13: Plotting Heatmap of Remaining Keywords

```
heatmap <- pheatmap(filtered_similarity_matrix,

  cluster_rows = FALSE,

  cluster_cols = FALSE,

  show_rownames = TRUE,

  display_numbers = TRUE,

  number_format = "%.0f",

  fontsize = 12,

  show_colnames = TRUE)

# Export the plot in various formats.

# Adjust the plot margins to add an extra ca. 2 mm of white space.

# Load the grid package (included in base R) and install and load the
    gridExtra package here, which may conflict with dplyr.

library(gridExtra)

library(grid)

# Define the white space to add around the plot (in inches).

```

```r
margin_size <- 0.079 # Approximately 2mm in inches to approximate about a 2mm border of white space.

# Create a blank plot for margins.

blank_plot <- grid.rect(gp = gpar(col = "white"))

# Combine the heatmap with the blank plot for the margins.

combined_plot <- gridExtra::arrangeGrob(blank_plot, blank_plot, blank_plot, blank_plot, heatmap$gtable, blank_plot, blank_plot, blank_plot, blank_plot, ncol = 3, nrow = 3, widths = unit(c(margin_size, 7.5 - 2 * margin_size, margin_size), "in"), heights = unit(c(margin_size, 7.5 - 2 * margin_size, margin_size), "in"))

# Export as PDF.

ggsave(filename = "Fig2.pdf",

  plot = combined_plot,

  device = "pdf",

  path = "R_Objects/Keywords",

  width = 7.5, height = 7.5, units = "in")

# Export as TIFF.

ggsave(filename = "Fig2.tiff",

  plot = combined_plot,

  device = "tiff",

  path = "R_Objects/Keywords",

  width = 7.5, height = 7.5, units = "in", dpi = 300)

# Export as PNG.

ggsave(filename = "Fig2.png",
```

```
  plot = combined_plot,
  device = "png",
  path = "R_Objects/Keywords",
  width = 7.5, height = 7.5, units = "in", dpi = 300)
# Remove gridExtra and reload dplyr.
detach("package:gridExtra", unload = TRUE)
library(dplyr)
```

Clean up the global environment.

Listing 9.14: Cleaning up the Global Environment After Keyword Identification

```
# Save the objects needed for later steps and/or for auditing our work.
saveRDS(gift_only_dfm51, "R_Objects/Keywords/gift_only_dfm51.rds")
saveRDS(gift_only_keywords51, "R_Objects/Keywords/gift_only_keywords51.
    rds")
saveRDS(gift_only_keywords51_filtered, "R_Objects/Keywords/gift_only_
    keywords51_filtered.rds")
saveRDS(gift_only_keywords51_significant, "R_Objects/Keywords/gift_only_
    keywords51_significant.rds")
saveRDS(token_counts, "R_Objects/Keywords/token_counts.rds")
saveRDS(corpus_sizes, "R_Objects/Keywords/corpus_sizes.rds")
saveRDS(normalization_factor_gift, "R_Objects/Keywords/normalization_
```

```
factor_gift.rds")

saveRDS(normalization_factor_only, "R_Objects/Keywords/normalization_factor_only.rds")

saveRDS(keywords, "R_Objects/Keywords/keywords.rds")

saveRDS(lemmatized_result, "R_Objects/Keywords/lemmatized_result.rds")

saveRDS(lemmatize_keywords_result, "R_Objects/Keywords/lemmatize_keywords_result.rds")

saveRDS(synonyms_result, "R_Objects/Keywords/synonyms_result.rds")

saveRDS(similarity_matrix, "R_Objects/Keywords/similarity_matrix.rds")

saveRDS(count_greater_than_zero, "R_Objects/Keywords/count_greater_than_zero.rds")

saveRDS(cell_count_overall, "R_Objects/Keywords/cell_count_overall.rds")

saveRDS(rows_to_keep, "R_Objects/Keywords/rows_to_keep.rds")

saveRDS(cols_to_keep, "R_Objects/Keywords/cols_to_keep.rds")

saveRDS(filtered_similarity_matrix, "R_Objects/Keywords/filtered_similarity_matrix.rds")

saveRDS(non_zero_values, "R_Objects/Keywords/non_zero_values.rds")

saveRDS(value_counts, "R_Objects/Keywords/value_counts.rds")

saveRDS(modal_value, "R_Objects/Keywords/modal_value.rds")

# Retrieve the names of all objects in the global environment and store them in a character vector.

all_objects <- ls()

# Create a character vector of object names in the global environment.

formatted_object_array <- paste('c("', paste(all_objects, collapse = '",
```

```
␣"'), '")', sep = "")

# Export a formatted list to a .txt file for manual review.

write(formatted_object_array, "R_objects.txt")

# Manually review the .txt file and delete objects to be kept in the R global environment.

# Import the first line of the modified .txt file back into R.

string_of_object_names <- readLines("R_objects.txt", n = 1)

# Convert the string in the .txt file back into a character vector of object names.

formatted_object_array <- eval(parse(text = string_of_object_names))

# Remove the objects selected for removal from the global environment.

rm(list = c(formatted_object_array))

rm(list = c("all_objects", "formatted_object_array", "string_of_object_names"))

# Invoke the garbage collection function gc() to release memory occupied by no-longer-used objects and to compact the storage of remaining objects in the global environment to improve memory usage.

gc()
```

10 Theme Identification

Identify themes in the *gift** passages using principal component analysis (PCA), following approaches as set out in recent methods literature [10, 21].

Load the lemmatized tokens object `gift_only_tokens_lemmatized51` back into the global environment (as needed) and extract the relevant data.

Listing 10.1: Preparing for the Theme Identification

```
gift_only_tokens_lemmatized51 <- readRDS("R_Objects/Corpora/gift_only_tokens_lemmatized51.rds")

# Subset the 'gift*' passages.

gift_tokens_lemmatized51 <- tokens_subset(gift_only_tokens_lemmatized51, SampleType == "gift")

class(gift_tokens_lemmatized51)

str(gift_tokens_lemmatized51)

head(gift_tokens_lemmatized51)

# Convert the tokenized 'gift*' passages into a document-feature matrix.

gift_dfm51 <- dfm(gift_tokens_lemmatized51)

class(gift_dfm51)

str(gift_dfm51)

dim(gift_dfm51)

gift_dfm51[,1:10]
```

10.1 Determining a Document-Exclusion Threshold

Exclude words from the document–feature matrix that occur infrequently across the 2,104 *gift** passages. Base the inclusion threshold and threshold-clarification procedure on recent best-practice guidelines [10, 21] for using PCA to identify themes in texts. One guideline notes that the final threshold specification is “dataset-dependent” ([21], p. 4) and advises to exclude words that appear in low percentages of texts while still yielding a “meaningful and interpretable number of themes” ([21], p. 5). The same guideline mentions the exclusion of words that appear in fewer than or equal to 5 percent of texts as an example of one possible exclusion threshold for this type of approach. To determine a threshold value that excludes words that are infrequent across our *gift** passages, whilst also keeping sufficient words in the document–feature matrix to form the basis for PCA-derived findings (i. e., themes) that are meaningful and interpretable, implement

an iterative process of testing possible thresholds starting with the noted 5-percent threshold.

For each possible threshold value, create the resulting filtered document–feature matrix, binarize the matrix for PCA (as advised in the same guidelines[10, 21]), and carry out an initial PCA to see whether the resulting components provide themes that are meaningful and interpretable.

Calculate document frequency for the unfiltered document–feature matrix. That is, for each word type (column), count how many times that word type has a value other than 0 for a given row (cell). Use the apply() function with the second argument set to "2" for column-wise application of the function.

Listing 10.2: Calculating Document Frequency

```
doc_freq51 <- apply(gift_dfm51 , 2, function(x) sum(x > 0))
```

Calculate possible word-inclusion thresholds for document frequencies starting at 5 percent of texts.

Listing 10.3: Calculating Possible Word-Inclusion Thresholds

```
Threshold5percent <- round(0.05 * length(gift_dfm51@Dimnames$docs))

Threshold2.5percent <- round(0.025*length(gift_dfm51@Dimnames$docs))

Threshold1.25percent <- round(0.0125*length(gift_dfm51@Dimnames$docs))
```

Identify the columns to be retained according to each possible document-inclusion threshold.

Listing 10.4: Identifying Columns to Be Retained

```
cols_to_keep51_105 <- names(doc_freq51[doc_freq51 >= Threshold5percent])

cols_to_keep51_53 <- names(doc_freq51[doc_freq51 >= Threshold2.5percent
```

```
    ])

cols_to_keep51_26 <- names(doc_freq51[doc_freq51 >= Threshold1.25percent
    ])
```

Use the three possible vectors of columns to keep to subset the full version of the document–feature matrix. Then review the number of documents retained in each version of the subset matrix.

Listing 10.5: Subsetting the Full Matrix With Possible Vectors of Columns to Keep

```
gift_dfm51_105 <- gift_dfm51[, cols_to_keep51_105]

gift_dfm51_53 <- gift_dfm51[, cols_to_keep51_53]

gift_dfm51_26 <- gift_dfm51[, cols_to_keep51_26]

dim(gift_dfm51_105)

dim(gift_dfm51_53)

dim(gift_dfm51_26)
```

Convert the Quanteda document–feature matrices to base-R matrices, as this is needed for the subsequent step.

Listing 10.6: Converting to Base-R Matrices

```
gift_data_matrix51_105 <- as.matrix(gift_dfm51_105)

gift_data_matrix51_53 <- as.matrix(gift_dfm51_53)

gift_data_matrix51_26 <- as.matrix(gift_dfm51_26)
```

Mutate the base-R matrices into binary matrices. Following the aforementioned guideline,[10, 21] this is a prerequisite step before performing PCA on the three possible matrices.

Listing 10.7: Mutating the Base-R Matrices Into Binary Matrices

```
# Convert the matrices into a data frames.

gift_data_df51_105 <- as.data.frame(gift_data_matrix51_105)

gift_data_df51_53 <- as.data.frame(gift_data_matrix51_53)

gift_data_df51_26 <- as.data.frame(gift_data_matrix51_26)

# Convert all non-zero values to 1.

binary_df51_105 <- gift_data_df51_105 %>%

  mutate_all(~ifelse(. != 0, 1, 0))

binary_df51_53 <- gift_data_df51_53 %>%

  mutate_all(~ifelse(. != 0, 1, 0))

binary_df51_26 <- gift_data_df51_26 %>%

  mutate_all(~ifelse(. != 0, 1, 0))

# Convert the data frames back into matrices.

binary_matrix51_105 <- as.matrix(binary_df51_105)

binary_matrix51_53 <- as.matrix(binary_df51_53)

binary_matrix51_26 <- as.matrix(binary_df51_26)
```

Run a parallel analysis [18] on each possible binary document–feature matrix with the `fa.parallel()` function [27] to find an optimal number of components to keep for the given matrix. In consideration of the non-deterministic nature of parallel analysis, run the analysis 100 times per possible matrix and take the modal value for suggested number of components.

Listing 10.8: Running Three Preliminary Parallel Analyses

```
# Create empty vectors to store the results.

component_suggestions51_105 <- numeric(100)

component_suggestions51_53 <- numeric(100)

component_suggestions51_26 <- numeric(100)

# Set the numbers of runs.

num_runs51_105 <- 100

num_runs51_53 <- 100

num_runs51_26 <- 100

# Create loops to run fa.parallel() for each possible matrix and store
    the factor number suggestions.

for (i in 1:num_runs51_105) {

  # Run fa.parallel().

  result51_105 <- fa.parallel(binary_matrix51_105, fm = "minres")

  # Extract the suggested number of components from the result.

  component_suggestions51_105[i] <- result51_105$ncomp

}

for (i in 1:num_runs51_53) {

  # Run fa.parallel().

  result51_53 <- fa.parallel(binary_matrix51_53, fm = "minres")

  # Extract the suggested number of components from the result.
```

```

  component_suggestions51_53[i] <- result51_53$ncomp

}

for (i in 1:num_runs51_26) {

  # Run fa.parallel().

  result51_26 <- fa.parallel(binary_matrix51_26, fm = "minres")

  # Extract the suggested number of components from the result.

  component_suggestions51_26[i] <- result51_26$ncomp

}

# Calculate the modal value for each vector of component suggestions (
    for each possible matrix). Load the modeest package only here for
    finding the modal value via its mlv() function, because the modeest
    package conflicts with the psych package.

library(modeest)

mlv(component_suggestions51_105, method = "mfv")

mlv(component_suggestions51_53, method = "mfv")

mlv(component_suggestions51_26, method = "mfv")

# Detach the modeest package and reload the psych package.

detach("package:modeest", unload = TRUE)

library(psych)
```

A 1-component PCA result cannot provide meaningful information about possible themes. Hence, exclude the 5-percent threshold option from consideration. Consider the matrices created with the 2.5-percent and 1.25-percent inclusion thresholds by running initial PCAs using the respective numbers of recommended components from the parallel analysis.

Start by running PCAs for the two remaining predetermined possible inclusion thresholds. For each, review

the resulting components' words to assess whether they yield themes that are meaningful and interpretable.

Listing 10.9: Running Two Preliminary PCAs

```
pca_results_51_53 <- run_pca_analysis(binary_matrix51_53, 7, "binary_
    matrix51_53")

View(pca_results_51_53$Loadings)

pca_results_51_26 <- run_pca_analysis(binary_matrix51_26, 37, "binary_
    matrix51_36")

View(pca_results_51_26$Loadings)
```

The 2.5-percent inclusion threshold (i. e., the 53-word inclusion threshold) yields a small number of uninterpretable components. The 1.25-percent inclusion threshold (i. e., the 26-word inclusion threshold) yields a large number of mostly uninterpretable components. The result of the 1.25-percent inclusion threshold shows some signs of meaningful topics among its components.

Create ten new matrices for additional possible inclusion thresholds that are gradually higher than the 1.25-percent inclusion threshold, moving upwards in whole numbers in increments of 1 from 27 to 36. For each of these, also run a parallel analysis to find a recommended number of components for conducting a PCA on that matrix. Then run a PCA for each new binarized document–feature matrix and review the resulting components to see whether they yield themes that are meaningful and interpretable.

Define a function to run the entire preparatory process of creating additional matrices with the new inclusion thresholds, running a parallel analysis for each, and calculating the modal value for each parallel analysis to derive a recommended number of components for each new matrix for a subsequent PCA.

Listing 10.10: Preparing Ten Additional Possible Matrices

```
run_analysis <- function(original_matrix, cols_to_keep, num_runs = 100)
    {

  # Subset the original matrix.
```

```
subset_matrix <- original_matrix[, cols_to_keep]

# Convert the subset matrix to a base-R matrix.

data_matrix <- as.matrix(subset_matrix)

# Mutate the base-R matrix into a binary matrix.

data_df <- as.data.frame(data_matrix)

binary_df <- data_df %>%

  mutate_all(~ifelse(. != 0, 1, 0))

binary_matrix <- as.matrix(binary_df)

# Run a parallel analysis for each matrix to find a recommended modal
   value for the number of components for running a subsequent PCA.

component_suggestions <- numeric(num_runs)

for (i in 1:num_runs) {

  result <- fa.parallel(binary_matrix, fm = "minres")

  component_suggestions[i] <- result$ncomp

}

# Calculate the modal value for the number of components.

library(modeest)

mode_value <- mlv(component_suggestions, method = "mfv")

# Detach the modeest package and reload the psych package.

detach("package:modeest", unload = TRUE)

library(psych)
```

```
  # Return both the modal values and the binary matrices for the 10 new
      matrices (based on the additional possible inclusion thresholds)
      for use in the next step.

  return(list(mode_value = mode_value, binary_matrix = binary_matrix))

}

# Define the original matrix and column vectors to keep.

original_matrix <- gift_dfm51

# Define column vectors for each additional desired threshold.

cols_to_keep_list <- list(

  cols_to_keep51_27 = names(doc_freq51[doc_freq51 >= 27]),

  cols_to_keep51_28 = names(doc_freq51[doc_freq51 >= 28]),

  cols_to_keep51_29 = names(doc_freq51[doc_freq51 >= 29]),

  cols_to_keep51_30 = names(doc_freq51[doc_freq51 >= 30]),

  cols_to_keep51_31 = names(doc_freq51[doc_freq51 >= 31]),

  cols_to_keep51_32 = names(doc_freq51[doc_freq51 >= 32]),

  cols_to_keep51_33 = names(doc_freq51[doc_freq51 >= 33]),

  cols_to_keep51_34 = names(doc_freq51[doc_freq51 >= 34]),

  cols_to_keep51_35 = names(doc_freq51[doc_freq51 >= 35]),

  cols_to_keep51_36 = names(doc_freq51[doc_freq51 >= 36])

)

# Create an empty list to store results.

all_modes <- list()

```

```
# Loop through each set of columns to keep, run the parallel analyses,
    calculate the modal values, and return these.

for (name in names(cols_to_keep_list)) {

  cols_to_keep <- cols_to_keep_list[[name]]

  mode_value <- run_analysis(original_matrix, cols_to_keep)

  all_modes[[name]] <- mode_value

}

# Print the resulting modal values for the ten new matrices.

modal_values_ten_additional_matrices <- sapply(all_modes, function(x) x$
    mode_value)

print(modal_values_ten_additional_matrices)
```

Now run PCAs for the additional matrices based on the additional new inclusion thresholds, using the recommended numbers of components returned in the previous step from the parallel analyses. For each additional PCA result, assess whether it yields themes (i. e., lists of words that together load onto a given component) that are meaningful and interpretable. If more than one result provides meaningful and interpretable themes, select the result with the most coherent set of themes for inclusion in the study.

Listing 10.11: Running Preliminary PCAs on the Ten Additional Possible Matrices

```
# Create an empty list to store results from multiple PCA runs.

all_10_pca_results <- list()

# Create a list of matrices and their corresponding numbers of component
    values for PCA analysis.

# Extract the ten binary matrices from the 'all_modes' object.

```

```
binary_matrices <- lapply(all_modes, function(x) x$binary_matrix)

# Now create the list of ten matrices and parallel-analysis-derived
    recommended numbers of components.

matrices_and_components <- list(

  list(matrix = binary_matrices$cols_to_keep51_36, nfactors = modal_
     values_ten_additional_matrices["cols_to_keep51_36"], name = "binary
     _matrix51_36"),

  list(matrix = binary_matrices$cols_to_keep51_35, nfactors = modal_
     values_ten_additional_matrices["cols_to_keep51_35"], name = "binary
     _matrix51_35"),

  list(matrix = binary_matrices$cols_to_keep51_34, nfactors = modal_
     values_ten_additional_matrices["cols_to_keep51_34"], name = "binary
     _matrix51_34"),

  list(matrix = binary_matrices$cols_to_keep51_33, nfactors = modal_
     values_ten_additional_matrices["cols_to_keep51_33"], name = "binary
     _matrix51_33"),

  list(matrix = binary_matrices$cols_to_keep51_32, nfactors = modal_
     values_ten_additional_matrices["cols_to_keep51_32"], name = "binary
     _matrix51_32"),

  list(matrix = binary_matrices$cols_to_keep51_31, nfactors = modal_
     values_ten_additional_matrices["cols_to_keep51_31"], name = "binary
     _matrix51_31"),

  list(matrix = binary_matrices$cols_to_keep51_30, nfactors = modal_
     values_ten_additional_matrices["cols_to_keep51_30"], name = "binary
     _matrix51_30"),

  list(matrix = binary_matrices$cols_to_keep51_29, nfactors = modal_
     values_ten_additional_matrices["cols_to_keep51_29"], name = "binary
     _matrix51_29"),

  list(matrix = binary_matrices$cols_to_keep51_28, nfactors = modal_
     values_ten_additional_matrices["cols_to_keep51_28"], name = "binary
     _matrix51_28"),
```

```
  list(matrix = binary_matrices$cols_to_keep51_27, nfactors = modal_values_ten_additional_matrices["cols_to_keep51_27"], name = "binary_matrix51_27")

)

# Now run PCAs on all of the matrices stored in the 'matrices_and_components' object and write the outputs to an 'all_10_pca_results' object for later review. Use the matrix-specific recommended numbers of components (from the parallel analysis) that are also stored in the 'matrices_and_components' object.

for (mf in matrices_and_components) {

  result <- run_pca_analysis(mf$matrix, mf$nfactors, mf$name)

  all_10_pca_results[[mf$name]] <- result

}
```

Review the loadings component by component and all together from each PCA (i. e., for each matrix with a given possible inclusion threshold) to assess whether and to which extent that PCA yielded meaningful and interpretable themes.

Listing 10.12: Reviewing the PCA Loadings From the 10 Additional Possible Inclusion Thresholds

```
# Review the PCA component loadings result for the matrix with the 36-word inclusion threshold.

View(all_10_pca_results$binary_matrix51_36$Loadings)

# Review the PCA component loadings result for the matrix with the 35-word inclusion threshold.

View(all_10_pca_results$binary_matrix51_35$Loadings)

```

```
# Review the PCA component loadings result for the matrix with the 34-
    word inclusion threshold.

View(all_10_pca_results$binary_matrix51_34$Loadings)

# Review the PCA component loadings result for the matrix with the 33-
    word inclusion threshold.

View(all_10_pca_results$binary_matrix51_33$Loadings)

# Review the PCA component loadings result for the matrix with the 32-
    word inclusion threshold.

View(all_10_pca_results$binary_matrix51_32$Loadings)

# Review the PCA component loadings result for the matrix with the 31-
    word inclusion threshold.

View(all_10_pca_results$binary_matrix51_31$Loadings)

# Review the PCA component loadings result for the matrix with the 30-
    word inclusion threshold.

View(all_10_pca_results$binary_matrix51_30$Loadings)

# Review the PCA component loadings result for the matrix with the 29-
    word inclusion threshold.

View(all_10_pca_results$binary_matrix51_29$Loadings)

# Review the PCA component loadings result for the matrix with the 28-
    word inclusion threshold.

View(all_10_pca_results$binary_matrix51_28$Loadings)

# Review the PCA component loadings result for the matrix with the 27-
    word inclusion threshold.

View(all_10_pca_results$binary_matrix51_27$Loadings)
```

The PCA results for the matrices with the 36-word, 35-word, and 34-word inclusion thresholds yielded generally meaningful and interpretable components (themes) respectively. The PCA results for the matrices

with lower thresholds yielded results that are hard to interpret. We selected the PCA result from the 34-word inclusion threshold, because it provided the overall most meaningful and interpretable set of results.

10.2 Preparing and Implementing the Theme-Identification Procedure

Using the 34-word inclusion threshold, now focus the PCA investigation on a document–feature matrix including all words in the `gift_dfm51` object that occur in at least 34 different documents (rows). For the sake of better explanation and easier replication, we will repeat some of the steps described durnig the investigation of the best inclusion threshold.

Describe the object before and after implementing the filtering process.

Listing 10.13: Filtering the `gift_dfm51` Object

```
# Describe the length variability of all documents.

doc_lengths_gift_dfm51 <- rowSums(gift_dfm51)

summary(doc_lengths_gift_dfm51)

# Identify the columns to be retained (i.e., those columns for which doc
    _freq is 34 or greater) using the 'doc_freq51' object calculated
    previously.

cols_to_keep51_34 <- names(doc_freq51[doc_freq51 >= 34])

head(cols_to_keep51_34)

summary(cols_to_keep51_34)

# Use the vector of columns to keep to subset the previous version of
    the matrix.

gift_dfm51_34 <- gift_dfm51[, cols_to_keep51_34]

dim(gift_dfm51_34)

# Describe the length variability of the documents after excluding the
    less frequently occurring word types.

```

```
doc_lengths_gift_dfm51_34 <- rowSums(gift_dfm51_34)

summary(doc_lengths_gift_dfm51_34)

sd(doc_lengths_gift_dfm51_34)
```

Convert the Quanteda document–feature matrix to a base-R matrix and describe the resulting matrix.

Listing 10.14: Converting to a Base-R Matrix and Describing It

```
gift_data_matrix51_34 <- as.matrix(gift_dfm51_34)

ncol(gift_data_matrix51_34)

# Describe the distribution of counts in the matrix. Start by
    calculating its sparsity.

total_cells51_34 <- ncol(gift_data_matrix51_34) * nrow(gift_data_
    matrix51_34)

zero_cells51_34 <- sum(gift_data_matrix51_34 == 0)

sparsity51_34 <- zero_cells51_34 / total_cells51_34

print(sparsity51_34)

# Calculate basic descriptive statistics for the non-0 elements.

non_zero_elements51_34 <- gift_data_matrix51_34[gift_data_matrix51_34 !=
    0]

basic_stats51_34 <- summary(non_zero_elements51_34)

print(basic_stats51_34)

# Show the distribution of frequencies.

```

```
hist(non_zero_elements51_34, main="Frequency␣Distribution␣of␣Non-Zero␣Elements",
      xlab="Value", ylab="Frequency")
flat_matrix51_34 <- as.vector(gift_data_matrix51_34)
freq_table_base51_34 <- table(flat_matrix51_34)
print(freq_table_base51_34)
```

Mutate lstinlinegift_data_matrix51_34 into a binary matrix and describe the resulting matrix.

Listing 10.15: Mutating the Base-R Matrix Into a Binary Matrix and Describing It

```
# Convert the matrix to a data frame.
gift_data_df51_34 <- as.data.frame(gift_data_matrix51_34)
# Convert all non-zero values to 1.
binary_df51_34 <- gift_data_df51_34 %>%
  mutate_all(~ifelse(. != 0, 1, 0))
# Convert data frame back to a matrix.
binary_matrix51_34 <- as.matrix(binary_df51_34)
dim(binary_matrix51_34)
# Calculate the sparsity of the binary matrix. Start by counting the number of zeros in the matrix.
num_zeros51_34 <- sum(binary_matrix51_34 == 0)
# Calculate the total number of entries in the binary matrix.
```

```
total_entries51_34 <- length(binary_matrix51_34)

# Calculate the proportion of zeros.

proportion_zeros51_34 <- num_zeros51_34 / total_entries51_34

# The value should be equal to that of the 'sparsity51_34' object (above), indicating that the conversion to a binary matrix worked as expected.

print(proportion_zeros51_34)

dim(binary_matrix51_34)
```

Examine the Kaiser–Meyer–Olkin Measure of Sampling Adequacy and Bartlett's Test of Sphericity.

Listing 10.16: Assessing Sampling Adequacy and Sphericity

```
correlation_matrix51_34 <- cor(binary_matrix51_34, method = "pearson")

kmo_result51_34 <- KMO(correlation_matrix51_34)

print(paste("KMO Value: ", kmo_result51_34$MSA[1]))

n_samples51_34 <- nrow(binary_matrix51_34)

bartlett_result51_34 <- cortest.bartlett(correlation_matrix51_34, n = n_samples51_34)

print(paste("Bartlett's Test: Chi-square = ", bartlett_result51_34$chisq,

  ", df = ", bartlett_result51_34$df,

  ", p-value = ", format.pval(bartlett_result51_34$p.value, digits = 2)))
```

Run a parallel analysis [18] on the binary matrix with the `fa.parallel()` function [27] to find an optimal number of components to keep. In consideration of the non-deterministic nature of parallel analysis, run the analysis 100 times and take the modal value for suggested number of components.

In the next listing we include the code needed to run a parallel analysis on the `binary_matrix51_34` object. However, as different iterations of a parallel analysis can yield slightly different values, we skipped this listing and instead extracted the modal value previously ascertained for the version of document–feature matrix with the 34-word inclusion threshold, as documented in Listing 10.18.

Listing 10.17: Running a Parallel Analysis

```
# Create an empty vector to store the results.

component_suggestions51_34 <- numeric(100)

# Set the number of runs.

num_runs51_34 <- 100

# Create a loop to run fa.parallel() and store factor suggestions.

for (i in 1:num_runs51_34) {

  # Run fa.parallel().

  result51_34 <- fa.parallel(binary_matrix51_34, fm = "minres")

  # Extract the suggested number of components from the result.

  component_suggestions51_34[i] <- result51_34$ncomp

}

# View the vector of component suggestions and calculate the modal value. Load the modeest package only here for finding the modal value via its mlv() function, because the modeest package conflicts with the psych package.

library(modeest)

```

```
print(component_suggestions51_34)

mlv(component_suggestions51_34, method = "mfv")

# Detach the modeest package and reload the psych package.

detach("package:modeest", unload = TRUE)

library(psych)
```

If the previous listing is skipped as per our suggestion, then extract the modal value for the `binary_matrix51_34` object from the `all_modes` list object created previously in Listing 10.10.

Listing 10.18: Extracting the Modal Value for `binary_matrix51_34` from `all_modes`

```
PA_binary_matrix51_34_mode <- all_modes$cols_to_keep51_34$mode_value

print(PA_binary_matrix51_34_mode)
```

As reported already after Listing 10.10, the modal value was 20 for the 100 suggested numbers of components we calculated. We then implemented the PCA with the number of components set at 20. Due to the non-deterministic nature of parallel analysis, its replication can yield slightly different values.

Implement the principal component analysis (PCA) using the `principal()` function in the psych package [27]. Use varimax rotation as per recommendations for PCA for quantitative text analysis [10, 21].

Listing 10.19: Implementing a Principal Component Analysis (PCA)

```
PCAresult51_34_20 <- principal(binary_matrix51_34, nfactors = 20, rotate
    = "varimax")

print(PCAresult51_34_20)
```

```

class(PCAresult51_34_20)

str(PCAresult51_34_20)

names(PCAresult51_34_20)
```

Create an overview in tibble format reporting loadings, proportion of variance, cumulative variance, proportion of variance explained, and cumulative proportion of explained variance. Pull the loadings from `PCAresult51_34_20$loadings` and the other values from `PCAresult51_34_20$Vaccounted`. In the latter object, the relevant metrics are, as per [27], "Sum of Squares loadings," "Proportion Var," and "Cumulative Var." "Sum of Squares loadings" is the sum of the squared component loadings associated with each original variable for a given component. It represents the amount of variance that a specific principal component explains by accounting for how much each original variable contributes to that component. "Proportion Var" is the proportion of variance explained by a given component as a proportion of the variance of all variables. "Cumulative Var" is the cumulative variance explained by all components (in order) up to and including the current component as a proportion of the variance of all variables.

Listing 10.20: Creating an Overview of PCA Results

```
# Extract the component loadings, the variable names, and the component
    names.

loadings_matrix_51_34_20 <- PCAresult51_34_20$loadings

dim(loadings_matrix_51_34_20)

variables51_34_20 <- rownames(loadings_matrix_51_34_20)

components51_34_20 <- colnames(loadings_matrix_51_34_20)

# Format the loadings matrix into a long-form data frame. Each row
    reflects a single variable-component loading value.

tidy_loadings51_34_20 <- data.frame(

```

```
  Variable = rep(variables51_34_20, times = length(components51_34_20)), # So 93 variables * 20 components.
  Component = rep(components51_34_20, each = length(variables51_34_20)), # Repeat each component name for each variable.
  Value = as.vector(loadings_matrix_51_34_20) # The loading values are put into one column.
)
# Retain loadings >= 0.2 and rename "RCn" as "Component n".
filtered_loadings51_34_20 <- tidy_loadings51_34_20 %>%
  filter(Value >= 0.2) %>%
  arrange(Component, desc(Value)) %>%
  mutate(Component = str_replace(Component, "RC", "Component␣"))
# Group loadings by component; place the loading values in parentheses after each variable; and limit to three decimal places. Now the tibble will have one row for each component.
formatted_loadings51_34_20 <- filtered_loadings51_34_20 %>%
  group_by(Component) %>%
  summarize(
    Loadings = paste0(Variable, "␣(", sprintf("%.3f", Value), ")", collapse = ",␣"),
    .groups = 'drop'
  ) %>%
  arrange(Component)
```

Prepare the variance metrics noted above.

Listing 10.21: Preparing the Display of the PCA Metrics

```
# Put the five metrics contained in 'PCAresult51_34_20$Vaccounted' into a long-form data frame.

variance_metrics_long51_34_20 <- as.data.frame(as.table(PCAresult51_34_20$Vaccounted))

# Add corresponding column names to the resulting long-form data frame.

colnames(variance_metrics_long51_34_20) <- c("Metric", "Component", "Value")

# Create a character vector with the names of the metrics we plan to add to the tibble (using the names imported from 'PCAresult51_34_20$Vaccounted').

metrics_of_interest51_34_20 <- c("SS loadings", "Proportion Var", "Cumulative Var")

# Filter 'variance_metrics_long51_34_20' for the metrics of interest.

variance_metrics_filtered51_34_20 <- variance_metrics_long51_34_20 %>%

  filter(Metric %in% metrics_of_interest51_34_20) %>%

  mutate(Component = str_replace(Component, "RC", "Component ")) %>%

  arrange(Component)

# Pivot such that the three metric names in Column 1 become four column names, and organize the rows according to the component names with the arrange() function.

variance_metrics_wide51_34_20 <- variance_metrics_filtered51_34_20 %>%

  pivot_wider(names_from = Metric, values_from = Value) %>%

  arrange(Component)
```

```
# Join 'formatted_loadings51_34_20' (two columns) and 'variance_metrics_
    wide51_34_20' (five columns) by the shared Component column.
final_tibble51_34_20 <- formatted_loadings51_34_20 %>%
  left_join(variance_metrics_wide51_34_20, by = "Component")
# Rename the columns with the desired wordings.
final_tibble51_34_20_renamed <- final_tibble51_34_20 %>%
  rename(
    `Sum of Squares Loadings` = `SS loadings`,
    `Proportion of Variance` = `Proportion Var`,
    `Cumulative Variance` = `Cumulative Var`
  )
# Round the values in the right two columns to five decimal places (to
    allow for three decimal places after manual conversion to percentages
     in the manuscript).
final_tibble51_34_20_rounded <- final_tibble51_34_20_renamed %>%
  mutate(
    `Sum of Squares Loadings` = round(`Sum of Squares Loadings`, 3),
    `Proportion of Variance` = round(`Proportion of Variance`, 5),
    `Cumulative Variance` = round(`Cumulative Variance`, 5)
  )
# Reorder the tibble based on the sum of squares loadings in descending
    order.
final_tibble51_34_20_sorted <- final_tibble51_34_20_rounded %>%
```

```
  arrange(desc(`Sum of Squares Loadings`))

# Preview the resulting tibble.

print(final_tibble51_34_20_sorted, n = 20)

View(final_tibble51_34_20_sorted)
```

Save the tibble as an Excel file for subsequent manual review and annotation, using the openxlsx package [28] to allow for pre-defining the format of the Excel columns. For the process of reviewing and annotating the component loadings, see the Results section of primary manuscript.

Listing 10.22: Exporting the PCA Results to Excel

```
# Add an empty left column for later manual annotation in the Excel
    environment.

final_tibble51_34_20_sorted_emptycol <- final_tibble51_34_20_sorted %>%

  mutate(`Name of Theme` = NA_character_) %>%

  select(`Name of Theme`, everything())

# Create a new workbook for the impending Excel import.

PCALoadingswbkeywords_34_20 <- createWorkbook()

# Add a worksheet to the workbook.

addWorksheet(PCALoadingswbkeywords_34_20, "Sheet1")

# Create styles.

header_style <- createStyle(fontName = "Calibri", fontColour = "black",
    textDecoration = "bold")

char_style <- createStyle(numFmt = "@")  # This is for text data.
```

```
numeric_with_decimals_style <- createStyle(numFmt = "0.00000")  # This
    allows for five digits after the decimal point.

# Write the tibble data to the worksheet.

writeData(PCALoadingswbkeywords_34_20, "Sheet1", final_tibble51_34_20_
    sorted_emptycol)

# Set column widths.

setColWidths(PCALoadingswbkeywords_34_20, sheet = "Sheet1", cols = 1,
    widths = "35")

setColWidths(PCALoadingswbkeywords_34_20, sheet = "Sheet1", cols = 2,
    widths = "15")

setColWidths(PCALoadingswbkeywords_34_20, sheet = "Sheet1", cols = 3,
    widths = "80")

setColWidths(PCALoadingswbkeywords_34_20, sheet = "Sheet1", cols = 4,
    widths = "22")

setColWidths(PCALoadingswbkeywords_34_20, sheet = "Sheet1", cols = 5,
    widths = "22")

setColWidths(PCALoadingswbkeywords_34_20, sheet = "Sheet1", cols = 6,
    widths = "22")

# Apply the column format styles to specific columns.

addStyle(PCALoadingswbkeywords_34_20, "Sheet1", style = header_style,
    rows = 1, cols = 1:6)

# Apply the character style with repetitions.

char_rows = rep(2:21, each = 3)  # For rows 2-21, each repeated thrice (
    for columns 1-3).

char_cols = rep(1:3, times = 20)  # For columns 1-3, each repeated 20
    times (to match rows 2-21).

```

```
addStyle(PCALoadingswbkeywords_34_20, "Sheet1", style = char_style, rows
     = char_rows, cols = char_cols)

# Apply 'numeric_with_decimals_style' with repetitions. The step is
    required because the openxlsx package maps the styles to the cells
    via Cartesian mapping.

num_rows = rep(2:21, each = 3)  # For rows 2-21, each repeated thrice (
    for columns 4-6)

num_cols = rep(4:6, times = 20)  # For columns 4-6, each repeated 20
    times (to match rows 2-21)

addStyle(PCALoadingswbkeywords_34_20, "Sheet1", style = numeric_with_
    decimals_style, rows = num_rows, cols = num_cols)

# Save the resulting Excel workbook.

saveWorkbook(PCALoadingswbkeywords_34_20, "R_Objects/PCA/PCALoadings51_
    34_20.xlsx", overwrite = FALSE)
```

Clean up the global environment.

Listing 10.23: Cleaning up the Global Environment After Theme Identification

```
# Save the objects needed for later steps and/or for auditing our work.

saveRDS(gift_tokens_lemmatized51, "R_Objects/PCA/gift_tokens_
    lemmatized51.rds")

saveRDS(gift_dfm51, "R_Objects/PCA/gift_dfm51.rds")

saveRDS(doc_freq51, "R_Objects/PCA/doc_freq51.rds")

saveRDS(Threshold5percent, "R_Objects/PCA/Threshold5percent.rds")

saveRDS(Threshold2.5percent, "R_Objects/PCA/Threshold2.5percent.rds")
```

```
saveRDS(Threshold1.25percent, "R_Objects/PCA/Threshold1.25percent.rds")

saveRDS(cols_to_keep51_105, "R_Objects/PCA/cols_to_keep51_105.rds")

saveRDS(cols_to_keep51_53, "R_Objects/PCA/cols_to_keep51_53.rds")

saveRDS(cols_to_keep51_26, "R_Objects/PCA/cols_to_keep51_26.rds")

saveRDS(gift_dfm51_105, "R_Objects/PCA/gift_dfm51_105.rds")

saveRDS(gift_dfm51_53, "R_Objects/PCA/gift_dfm51_53.rds")

saveRDS(gift_dfm51_26, "R_Objects/PCA/gift_dfm51_26.rds")

saveRDS(binary_df51_105, "R_Objects/PCA/binary_df51_105.rds")

saveRDS(binary_df51_53, "R_Objects/PCA/binary_df51_53.rds")

saveRDS(binary_df51_26, "R_Objects/PCA/binary_df51_26.rds")

saveRDS(result51_105, "R_Objects/PCA/result51_105.rds")

saveRDS(result51_53, "R_Objects/PCA/result51_53.rds")

saveRDS(result51_26, "R_Objects/PCA/result51_26.rds")

saveRDS(component_suggestions51_105, "R_Objects/PCA/component_
    suggestions51_105.rds")

saveRDS(component_suggestions51_53, "R_Objects/PCA/component_
    suggestions51_53.rds")

saveRDS(component_suggestions51_26, "R_Objects/PCA/component_
    suggestions51_26.rds")

saveRDS(pca_results_51_53, "R_Objects/PCA/pca_results_51_53.rds")

saveRDS(pca_results_51_26, "R_Objects/PCA/pca_results_51_26.rds")

saveRDS(all_modes, "R_Objects/PCA/all_modes.rds")
```

```
saveRDS(modal_values_ten_additional_matrices, "R_Objects/PCA/modal_
    values_ten_additional_matrices.rds")

saveRDS(all_10_pca_results, "R_Objects/PCA/all_10_pca_results.rds")

saveRDS(binary_matrices, "R_Objects/PCA/binary_matrices.rds")

saveRDS(matrices_and_components, "R_Objects/PCA/matrices_and_components.
    rds")

saveRDS(doc_lengths_gift_dfm51, "R_Objects/PCA/doc_lengths_gift_dfm51.
    rds")

saveRDS(cols_to_keep51_34, "R_Objects/PCA/cols_to_keep51_34.rds")

saveRDS(gift_dfm51_34, "R_Objects/PCA/gift_dfm51_34.rds")

saveRDS(doc_lengths_gift_dfm51_34, "R_Objects/PCA/doc_lengths_gift_dfm51
    _34.rds")

saveRDS(gift_data_matrix51_34, "R_Objects/PCA/gift_data_matrix51_34.rds"
    )

saveRDS(total_cells51_34, "R_Objects/PCA/total_cells51_34.rds")

saveRDS(zero_cells51_34, "R_Objects/PCA/zero_cells51_34.rds")

saveRDS(sparsity51_34, "R_Objects/PCA/sparsity51_34.rds")

saveRDS(non_zero_elements51_34, "R_Objects/PCA/non_zero_elements51_34.
    rds")

saveRDS(flat_matrix51_34, "R_Objects/PCA/flat_matrix51_34.rds")

saveRDS(freq_table_base51_34, "R_Objects/PCA/freq_table_base51_34.rds")

saveRDS(gift_data_df51_34, "R_Objects/PCA/gift_data_df51_34.rds")

saveRDS(binary_df51_34, "R_Objects/PCA/binary_df51_34.rds")

saveRDS(binary_matrix51_34, "R_Objects/PCA/binary_matrix51_34.rds")
```

```
saveRDS(num_zeros51_34, "R_Objects/PCA/num_zeros51_34.rds")

saveRDS(total_entries51_34, "R_Objects/PCA/total_entries51_34.rds")

saveRDS(proportion_zeros51_34, "R_Objects/PCA/proportion_zeros51_34.rds")

saveRDS(correlation_matrix51_34, "R_Objects/PCA/correlation_matrix51_34.rds")

saveRDS(kmo_result51_34, "R_Objects/PCA/kmo_result51_34.rds")

saveRDS(bartlett_result51_34, "R_Objects/PCA/bartlett_result51_34.rds")

saveRDS(PA_binary_matrix51_34_mode, "R_Objects/PCA/PA_binary_matrix51_34_mode.rds")

saveRDS(PCAresult51_34_20, "R_Objects/PCA/PCAresult51_34_20.rds")

saveRDS(loadings_matrix_51_34_20, "R_Objects/PCA/loadings_matrix_51_34_20.rds")

saveRDS(variables51_34_20, "R_Objects/PCA/variables51_34_20.rds")

saveRDS(components51_34_20, "R_Objects/PCA/components51_34_20.rds")

saveRDS(tidy_loadings51_34_20, "R_Objects/PCA/tidy_loadings51_34_20.rds")

saveRDS(filtered_loadings51_34_20, "R_Objects/PCA/filtered_loadings51_34_20.rds")

saveRDS(formatted_loadings51_34_20, "R_Objects/PCA/formatted_loadings51_34_20.rds")

saveRDS(variance_metrics_long51_34_20, "R_Objects/PCA/variance_metrics_long51_34_20.rds")

saveRDS(final_tibble51_34_20_rounded, "R_Objects/PCA/final_tibble51_34_20_rounded.rds")

saveRDS(final_tibble51_34_20_sorted, "R_Objects/PCA/final_tibble51_34_20
```

```
_sorted.rds")

# Retrieve the names of all objects in the global environment and store them in a character vector.

all_objects <- ls()

# Create a character vector of object names in the global environment.

formatted_object_array <- paste('c("', paste(all_objects, collapse = '", "'), '")', sep = "")

# Export a formatted list to a .txt file for manual review.

write(formatted_object_array, "R_objects.txt")

# Manually review the .txt file and delete objects to be kept in the R global environment.

# Import the first line of the modified .txt file back into R.

string_of_object_names <- readLines("R_objects.txt", n = 1)

# Convert the string in the .txt file back into a character vector of object names.

formatted_object_array <- eval(parse(text = string_of_object_names))

# Remove the objects selected for removal from the global environment.

rm(list = c(formatted_object_array))

rm(list = c("all_objects", "formatted_object_array", "string_of_object_names"))

# Invoke the garbage collection function gc() to release memory occupied by no-longer-used objects and to compact the storage of remaining objects in the global environment to improve memory usage.

gc()
```

11 Validation

We validate the themes identified in the PCA-based theme identification process by evaluating the differential prevalence of observed themes in the lemmatized *gift** and *only* corpora.

First, load the lemmatized tokens object `gift_only_tokens_lemmatized51` back into the global environment (as needed).

Listing 11.1: Preparing for the Validation

```
gift_only_tokens_lemmatized51 <- readRDS("R_Objects/Corpora/gift_only_
    tokens_lemmatized51.rds")

str(gift_only_tokens_lemmatized51)
```

Second, construct a set of theme-specific dictionaries (i.e., word lists) based on themes identified by the PCA using the Quanteda dictionary() function [12]. See the Validity Check subsection in the main manuscript for additional details on preparing the dictionaries based on the PCA results.

Listing 11.2: Constructing Theme-Specific Dictionaries

```
GiftedFictionThemes <- dictionary(list(

  FormalEducation = c("school", "high", "talent", "class", "run", "kid")
    ,

  YouthAndBoys = c("age", "boy", "student", "young", "year"),

  WrittenCommunication = c("write", "class", "time", "read", "special",
    "student"),

  ParentsAndArt = c("mother", "father", "leave", "art", "learn"),

  PotentNaturalAbility = c("bring", "begin", "world", "power", "great",
    "human", "ability", "natural", "possess"),
```

```

  SupernaturalAbility = c("magic", "family", "bear", "think"),

  Joy = c("laugh", "smile", "make", "run", "back"),

  VerbalCommunication = c("talk", "word", "people", "speak", "tell", "
     understand", "story", "true", "ask", "inside"),

  Music = c("play", "music", "love", "learn")

))
```

Third, extract individual key entries and their associated word lists as two separate vectors.

Listing 11.3: Extracting Individual Dictionaries

```
dictionary_names <- names(GiftedFictionThemes)

dictionary_list <- lapply(dictionary_names, function(x)
   GiftedFictionThemes[[x]])
```

Fourth, find and count matches in the documents using the dictionaries so extracted. This step requires defining a bespoke `find_matches_subcount()` function built around the `grepl()` function. The `grepl()` function returns a logical vector indicating whether a given term from the `patterns` object is present in a given entry in the `doc_tokens` object (i. e., the texts to be searched). The `sum()` function counts instances of TRUE (= 1) returned from the `grepl()` function. Each count issued by `sum()` is associated via the `sapply()` function with a unique compound string value. This string is created by combining the searched word pattern (from the `patterns` object) and the value (a character string) set during each iteration for the `prefix` argument. The `find_matches_subcount()` thus iterates over the `patterns` object, returning a vector called `counts`, in which each element represents the count of occurrences for a respective pattern in `doc_tokens`.

Listing 11.4: Finding and Counting Matches by Dictionary in the Documents

```
# Implement the bespoke function described above.

find_matches_subcount <- function(doc_tokens, patterns, prefix) {

  counts <- sapply(patterns, function(pattern) {

    sum(grepl(pattern, doc_tokens))

  })

  names(counts) <- paste0(prefix, "_", patterns)

  return(counts)

}

# Initialize a list to store the final counts for all documents.

final_results <- vector("list", length = length(gift_only_tokens_
    lemmatized51))

names(final_results) <- names(gift_only_tokens_lemmatized51)

# Loop through each document in the 'gift_only_tokens_lemmatized51'
    object.

for (doc_id in seq_along(gift_only_tokens_lemmatized51)) {

  # Retrieve tokens for the current document.

  current_doc_tokens <- gift_only_tokens_lemmatized51[[doc_id]]

  # Initialize a named vector to store counts for the current document.

  current_doc_counts <- integer(0)

  # Loop through each dictionary in 'dictionary_list'.

```

```
  for (dict_id in seq_along(dictionary_list)) {

    # Retrieve current dictionary patterns based on the value specified for 'dict_id' as per the value returned from the 'dictionary_list' by the seq_along() function.

    current_patterns <- dictionary_list[[dict_id]]

    # Specify the prefix (dictionary name).

    current_prefix <- dictionary_names[dict_id]

    # Use the find_matches_subcount() function to get counts for the current document and current dictionary.

    subcounts <- find_matches_subcount(current_doc_tokens, current_patterns, current_prefix)

    # Append these subcounts to the counts for the current document.

    current_doc_counts <- c(current_doc_counts, subcounts)

  }

  # Store the counts for the current document in the 'final_results' list.

  final_results[[doc_id]] <- current_doc_counts

}

str(final_results)

final_results[[3]]
```

Fifth, recast the resulting list object as a data frame for analysis and shorten the variable names while doing so.

Listing 11.5: Converting the List Object `final_results` to a Data Frame

```
# Convert the list elements to data frames and add a Text column.

final_results_dfs <- lapply(1:length(final_results), function(i) {

  temp_df <- as.data.frame(t(final_results[[i]]))

  temp_df$Text <- names(final_results)[i]

  return(temp_df)

})

# Bind the data frames together row-wise.

final_results_df <- do.call(rbind, final_results_dfs)

names(final_results_df)

# Rename the columns with abbreviated dictionary names to make working
    with the data frame easier.

colnames(final_results_df) <- gsub("^FormalEducation_", "FE_", colnames(
    final_results_df))

colnames(final_results_df) <- gsub("^YouthAndBoys_", "YaB_", colnames(
    final_results_df))

colnames(final_results_df) <- gsub("^WrittenCommunication_", "WC_",
    colnames(final_results_df))

colnames(final_results_df) <- gsub("^ParentsAndArt_", "PaA_", colnames(
    final_results_df))

colnames(final_results_df) <- gsub("^PotentNaturalAbility_", "PNA_",
    colnames(final_results_df))

colnames(final_results_df) <- gsub("^SupernaturalAbility_", "SA_",
    colnames(final_results_df))
```

```

colnames(final_results_df) <- gsub("^Joy_", "Joy_", colnames(final_
    results_df))

colnames(final_results_df) <- gsub("^VerbalCommunication_", "VC_",
    colnames(final_results_df))

colnames(final_results_df) <- gsub("^Music_", "Music_", colnames(final_
    results_df))

# Move the Text column to the first position.

final_results_df <- final_results_df[, c('Text', setdiff(names(final_
    results_df), 'Text'))]

names(final_results_df)

View(final_results_df)

str(final_results_df)

final_results_df[1,]
```

Sixth, add sum variables for each set of theme variables (i. e., for each dictionary set of word-based variables) and sum the counts for each word in each dictionary.

Listing 11.6: Adding Sum Variables for Each Dictionary

```
# Create a function to add sum columns for each category.

add_sum_columns <- function(df, prefix) {

  # Create names for the new columns by appending '_sum' to the prefixes
      stored in 'prefix'.

  sum_column <- paste0(prefix, "_sum")

```

```r
  # Identify columns to sum and return the actual names of these columns. In the grep() function, the argument 'value = TRUE' returns the column name rather than an index location. The regular expression '^' limits this process to those column names that start with a given prefix.
  cols_to_sum <- grep(paste0("^", prefix), names(df), value = TRUE)
  # Perform the row-wise addition on each row individually needed to imbue each new sum column with data.
  df <- df %>%
    # Do so row by row.
    rowwise() %>%
    # Use the names stored in the 'sum_column' object to create a new column and populate it with the sums resulting from the summing of all the columns that start with a given prefix. The characters `:=` are a so-called 'walrus operator' in the dplyr package. They assign names dynamically between objects on two sides of an assignment.
    mutate({{sum_column}} := sum(c_across(starts_with({{prefix}})))) # This is where the columns that share a prefix are actually being summed.
  # Return the new data frame.
  return(df)
}
# Create a list of prefixes for summing the columns by dictionary theme.
prefix_list <- c("FE", "YaB", "WC", "PaA", "PNA", "SA", "Joy", "VC", "Music")
# Sum the terms associated with each of the ten categories, using the newly created function for adding sum columns.
```

```
final_results_df_with_sums <- final_results_df

for (prefix in prefix_list) {

  final_results_df_with_sums <- add_sum_columns(final_results_df_with_sums, prefix)

}

# Un-group the data frame to remove the dplyr-induced grouped data attribute (to avoid incompatibility in later analyses).

final_results_df_with_sums <- ungroup(final_results_df_with_sums)

View(final_results_df_with_sums[1:6,])
```

Seventh, add back the (now missing) document-level variable (docvar) for sample type (SampleType) from the Quanteda object whence the texts where taken (i. e., from the `gift_only_tokens_lemmatized51` object).

Listing 11.7: Adding Back the Sample Type Docvar

```
# Get the docvars from the 'gift_only_tokens_lemmatized51' object.

docvars51 <- docvars(gift_only_tokens_lemmatized51)

# Bind these as columns on to the 'final_results_df_with_sums' object.

final_results_sums_df <- cbind(final_results_df_with_sums, docvars51)

names(final_results_sums_df)

# Rearrange the columns such that the new docvars (Year, SampleType, and Annual_Count) appear after the Text count name, followed by the sum variables.

final_results_sums_df <- final_results_sums_df %>%
```

```
  select(Text, Annual_Count, Year, SampleType, FE_sum, YaB_sum, WC_sum,
     PaA_sum, PNA_sum, SA_sum, Joy_sum, VC_sum, Music_sum, everything())

names(final_results_sums_df)

str(final_results_sums_df)

final_results_sums_df[1,]

gift_only_tokens_lemmatized51[1,1]

class(gift_only_tokens_lemmatized51)

gift_only_tokens_lemmatized51[1:3,]
```

Eighth, compare the mean sums of each theme in the *gift** versus the *only* passages. This step involves several substeps that will be presented in separate listings.

Start by recording the lengths of each text via the `gift_only_tokens_lemmatized51` object using the ntoken() helper function from Quanteda [7], which counts the tokens in each list entry within Quanteda tokens objects.

Listing 11.8: Recording the Lengths of Each Text

```
text_lengths <- ntoken(gift_only_tokens_lemmatized51)

# Reconstitute the data frame object, whilst inserting the 'text_lengths
   ' vector as the second column (using base-R subsetting).

final_results_sums_df <- data.frame(final_results_sums_df[, 1], Text_
   Length = text_lengths, final_results_sums_df[, -1])

# Correct the naming of the first column back to 'Text'. Due to a base-R
    convention for the subsetting we used, it has been inadvertently
   renamed.

```

```
names(final_results_sums_df)[1] <- "Text"

str(final_results_sums_df)
```

Add normalized counts for each sum variable.

Listing 11.9: Adding Normalized Counts for Each Sum Variable

```
# Loop through each sum variable prefix to identify the full variable
    name and normalize it against the values in '$Text_Length'.

for (prefix in prefix_list) {

  # Generate the full variable names for the sum variables.

  var_name <- paste0(prefix, "_sum")

  # Generate new variable names for recording the normalized counts.

  new_var_name <- paste0(var_name, "_norm")

  # Compute normalized counts and add them as new columns, using the
      ifelse() function to handle the 0 counts.

  final_results_sums_df[[new_var_name]] <- ifelse(final_results_sums_df$
      Text_Length > 0,

    final_results_sums_df[[var_name]] / final_results_sums_df$Text_
        Length,

    0)

}

str(final_results_sums_df)

View(final_results_sums_df)
```

```
names(final_results_sums_df)
```

Test the values we intend to compare for normality with the Kolmogorov–Smirnov Test with Lilliefors Correction for each group (used due to the large sample size instead of the Shapiro–Wilk Test). For simplicity's sake, send the outputs directly into locally saved .txt files for later review and reporting.

Listing 11.10: Testing the Validation Data for Normality

```
# 1

# Formal Education (FE)

sink("R_Objects/ThemesValidation/lillie.test_FE_gift.txt", append = TRUE
    )

lillie.test(final_results_sums_df$FE_sum_norm[final_results_sums_df$
    SampleType == "gift"])

sink()

sink("R_Objects/ThemesValidation/lillie.test_FE_only.txt", append = TRUE
    )

lillie.test(final_results_sums_df$FE_sum_norm[final_results_sums_df$
    SampleType == "only"])

sink()

# 2

# Youth and Boys (YaB)

sink("R_Objects/ThemesValidation/lillie.test_YaB_gift.txt", append =
    TRUE)

lillie.test(final_results_sums_df$YaB_sum_norm[final_results_sums_df$
```

```
    SampleType == "gift"])

sink()

sink("R_Objects/ThemesValidation/lillie.test_YaB_only.txt", append =
    TRUE)

lillie.test(final_results_sums_df$YaB_sum_norm[final_results_sums_df$
    SampleType == "only"])

sink()

# 3

# Written Communication (WC)

sink("R_Objects/ThemesValidation/lillie.test_WC_gift.txt", append = TRUE
    )

lillie.test(final_results_sums_df$WC_sum_norm[final_results_sums_df$
    SampleType == "gift"])

sink()

sink("R_Objects/ThemesValidation/lillie.test_WC_only.txt", append = TRUE
    )

lillie.test(final_results_sums_df$WC_sum_norm[final_results_sums_df$
    SampleType == "only"])

sink()

# 4

# Parents and Art (PaA)

sink("R_Objects/ThemesValidation/lillie.test_PaA_gift.txt", append =
    TRUE)

lillie.test(final_results_sums_df$PaA_sum_norm[final_results_sums_df$
    SampleType == "gift"])

```

```
sink()

sink("R_Objects/ThemesValidation/lillie.test_PaA_only.txt", append =
    TRUE)

lillie.test(final_results_sums_df$PaA_sum_norm[final_results_sums_df$
    SampleType == "only"])

sink()

# 5

# Potent Natural Ability (PNA)

sink("R_Objects/ThemesValidation/lillie.test_PNA_gift.txt", append =
    TRUE)

lillie.test(final_results_sums_df$PNA_sum_norm[final_results_sums_df$
    SampleType == "gift"])

sink()

sink("R_Objects/ThemesValidation/lillie.test_PNA_only.txt", append =
    TRUE)

lillie.test(final_results_sums_df$PNA_sum_norm[final_results_sums_df$
    SampleType == "only"])

sink()

# 6

# Supernatural Ability (SA)

sink("R_Objects/ThemesValidation/lillie.test_SA_gift.txt", append = TRUE
    )

lillie.test(final_results_sums_df$SA_sum_norm[final_results_sums_df$
    SampleType == "gift"])

sink()

```

```
sink("R_Objects/ThemesValidation/lillie.test_SA_only.txt", append = TRUE
    )

lillie.test(final_results_sums_df$SA_sum_norm[final_results_sums_df$
    SampleType == "only"])

sink()

# 7

# Joy (Joy)

sink("R_Objects/ThemesValidation/lillie.test_Joy_gift.txt", append =
    TRUE)

lillie.test(final_results_sums_df$Joy_sum_norm[final_results_sums_df$
    SampleType == "gift"])

sink()

sink("R_Objects/ThemesValidation/lillie.test_Joy_only.txt", append =
    TRUE)

lillie.test(final_results_sums_df$Joy_sum_norm[final_results_sums_df$
    SampleType == "only"])

sink()

# 8

# Verbal Communication (VC)

sink("R_Objects/ThemesValidation/lillie.test_VC_gift.txt", append = TRUE
    )

lillie.test(final_results_sums_df$VC_sum_norm[final_results_sums_df$
    SampleType == "gift"])

sink()

sink("R_Objects/ThemesValidation/lillie.test_VC_only.txt", append = TRUE
    )
```

```
lillie.test(final_results_sums_df$VC_sum_norm[final_results_sums_df$
    SampleType == "only"])

sink()

# 9

# Music (Music)

sink("R_Objects/ThemesValidation/lillie.test_Music_gift.txt", append =
    TRUE)

lillie.test(final_results_sums_df$Music_sum_norm[final_results_sums_df$
    SampleType == "gift"])

sink()

sink("R_Objects/ThemesValidation/lillie.test_Music_only.txt", append =
    TRUE)

lillie.test(final_results_sums_df$Music_sum_norm[final_results_sums_df$
    SampleType == "only"])

sink()
```

Test for equality of variances for each group with Levene's Test. For simplicity's sake, send the outputs directly into locally saved .txt files for later review and reporting.

Listing 11.11: Testing the Validation Data for Equality of Variances

```
# 1

# Formal Education (FE)

sink("R_Objects/ThemesValidation/leveneTest_FE.txt", append = TRUE)

```

```
leveneTest(final_results_sums_df$FE_sum_norm ~ final_results_sums_df$SampleType)

sink()

# 2

# Youth and Boys (YaB)

sink("R_Objects/ThemesValidation/leveneTest_YaB.txt", append = TRUE)

leveneTest(final_results_sums_df$YaB_sum_norm ~ final_results_sums_df$SampleType)

sink()

# 3

# Written Communication (WC)

sink("R_Objects/ThemesValidation/leveneTest_WC.txt", append = TRUE)

leveneTest(final_results_sums_df$WC_sum_norm ~ final_results_sums_df$SampleType)

sink()

# 4

# Parents and Art (PaA)

sink("R_Objects/ThemesValidation/leveneTest_PaA.txt", append = TRUE)

leveneTest(final_results_sums_df$PaA_sum_norm ~ final_results_sums_df$SampleType)

sink()

# 5

# Potent Natural Ability (PNA)

```

```
sink("R_Objects/ThemesValidation/leveneTest_PNA.txt", append = TRUE)

leveneTest(final_results_sums_df$PNA_sum_norm ~ final_results_sums_df$
    SampleType)

sink()

# 6

# Supernatural Ability (SA)

sink("R_Objects/ThemesValidation/leveneTest_SA.txt", append = TRUE)

leveneTest(final_results_sums_df$SA_sum_norm ~ final_results_sums_df$
    SampleType)

sink()

# 7

# Joy (Joy)

sink("R_Objects/ThemesValidation/leveneTest_Joy.txt", append = TRUE)

leveneTest(final_results_sums_df$Joy_sum_norm ~ final_results_sums_df$
    SampleType)

sink()

# 8

# Verbal Communication (VC)

sink("R_Objects/ThemesValidation/leveneTest_VC.txt", append = TRUE)

leveneTest(final_results_sums_df$VC_sum_norm ~ final_results_sums_df$
    SampleType)

sink()

# 9

```

```
# Music (Music)

sink("R_Objects/ThemesValidation/leveneTest_Music.txt", append = TRUE)

leveneTest(final_results_sums_df$Music_sum_norm ~ final_results_sums_df$
    SampleType)

sink()
```

Compare the means with Welch's *t* Test (a parametric test that does not require equal variances) as well as with the Mann–Whitney *U* Test (a non-parametric test that requires neither equal variances nor normal distributions). The parametric test should be sufficient despite the violation of normality in light of the large sample sizes. The Mann–Whitney *U* Test adds additional confidence that the test statistic is not merely reflecting artifacts of the data. Then assess the effect sizes of the differences with Cohen's *d* as well as with Cliff's delta, which does not require a specific shape or spread of the distribution.

Listing 11.12: Comparing Means in the Validation Data

```
# Initialize a tibble to store summary results.

summary_results_themes <- tibble()

# Create a list of normalized sum variables to test.

norm_sum_vars <- c("FE_sum_norm", "YaB_sum_norm", "WC_sum_norm", "PaA_
    sum_norm", "PNA_sum_norm", "SA_sum_norm", "Joy_sum_norm", "VC_sum_
    norm", "Music_sum_norm")

# Set the names of the themes.

theme_map <- list(

  FE = "Formal␣Education",

  YaB = "Youth␣and␣Boys",

  WC = "Written␣Communication",
```

```
  PaA = "Parents␣and␣Art",

  PNA = "Potent␣Natural␣Ability",

  SA = "Supernatural␣Ability",

  Joy = "Joy",

  VC = "Verbal␣Communication",

  Music = "Music"

)

# Loop through each normalized sum variable, effecting the needed tests
    and measurements: Mann-Whitney U Tests, Welch's t Tests, means, Cohen
    's d, and Cliff's delta.

for (var in norm_sum_vars) {

  # Extract the specific variable and sample type from the data frame.

  specific_var <- final_results_sums_df[[var]]

  sample_type <- final_results_sums_df$SampleType

  # Run the Mann-Whitney U Tests.

  mw_test_result <- wilcox.test(specific_var ~ sample_type)

  # Run Welch's t Tests.

  welch_test_result <- t.test(specific_var ~ sample_type, var.equal =
     FALSE)

  # Calculate the means for the 'gift*' and 'only' groups.

  mean_gift <- mean(specific_var[sample_type == 'gift'], na.rm = TRUE)

  mean_only <- mean(specific_var[sample_type == 'only'], na.rm = TRUE)

```

```
# Calculate Cohen's d.

cohens_d_result <- cohen.d(specific_var ~ sample_type)

# Calculate Cliff's delta.

cliffs_delta_result <- cliff.delta(specific_var[sample_type == 'gift'
    ], specific_var[sample_type == 'only'])

# Map a variable prefix to a theme if the variable suffix is "_sum_
    norm" using the base-R substr() function to match a certain
    numerical range within a character vector. Integrate an if-else
    loop to make sure we are only using the desired normalized
    variables in the 'final_results_sums_df' object, all nine of which
    end with "_sum_norm".

if (grepl("_sum_norm$", var)) {

  theme_abbreviation <- substr(var, 1, nchar(var) - 9)

  if (theme_abbreviation %in% names(theme_map)) {

    theme <- theme_map[[theme_abbreviation]]

    # Create a tibble row with the calculated and tested data outputs.

    row_tibble <- tibble(

      Theme = theme,

      `Mean ('gift*')` = mean_gift,

      `Mean ('only')` = mean_only,

      Welch = welch_test_result$statistic,

      `Welch df` = welch_test_result$parameter,

      `Welch p` = welch_test_result$p.value,

      `Welch 95% CI` = paste(welch_test_result$conf.int[1], "to",
        welch_test_result$conf.int[2]),
```

```r
        U = mw_test_result$statistic,
        `Mann-Whitney p` = mw_test_result$p.value,
        `Cohens d` = cohens_d_result$estimate,
        `Cliffs d` = cliffs_delta_result$estimate
      )
      # Add the new row to the summary tibble.
      summary_results_themes <- bind_rows(summary_results_themes, row_tibble)
    } else {
      warning(paste("Theme abbreviation not found in theme_map:", theme_abbreviation))
    }
  }
}
# Print out the summary tibble.
print(summary_results_themes)
```

Export the final tibble to Excel, using the openxlsx package [28] to allow for pre-defining the format of the Excel columns.

Listing 11.13: Exporting Validation Results

```
# Create a new workbook for the impending Excel import.

wb_summary_validation <- createWorkbook()

# Add a worksheet to the workbook.

addWorksheet(wb_summary_validation, "Sheet1")

# Create custom column format styles for the Excel export via openxlsx.

header_style <- createStyle(fontName = "Calibri", fontColour = "black",
    textDecoration = "bold")

char_style <- createStyle(numFmt = "@") # This is for text data.

numeric_with_decimals_style <- createStyle(numFmt = "0.000") # This
    allows for three digits after the decimal point.

# Export the data frame to the worksheet.

writeData(wb_summary_validation, "Sheet1", summary_results_themes)

# Define the number of rows to be formatted in the worksheet.

num_rows <- nrow(summary_results_themes)

# Apply the column format styles to specific columns.

addStyle(wb_summary_validation, "Sheet1", style = header_style, rows =
    1, cols = 1:11)

addStyle(wb_summary_validation, "Sheet1", style = char_style, rows = 2:(
    num_rows + 1), cols = 1)

addStyle(wb_summary_validation, "Sheet1", style = numeric_with_decimals_
    style, rows = 2:(num_rows + 1), cols = 2)

addStyle(wb_summary_validation, "Sheet1", style = numeric_with_decimals_
```

```
style, rows = 2:(num_rows + 1), cols = 3)

addStyle(wb_summary_validation, "Sheet1", style = numeric_with_decimals_style, rows = 2:(num_rows + 1), cols = 4)

addStyle(wb_summary_validation, "Sheet1", style = numeric_with_decimals_style, rows = 2:(num_rows + 1), cols = 5)

addStyle(wb_summary_validation, "Sheet1", style = numeric_with_decimals_style, rows = 2:(num_rows + 1), cols = 6)

addStyle(wb_summary_validation, "Sheet1", style = numeric_with_decimals_style, rows = 2:(num_rows + 1), cols = 7)

addStyle(wb_summary_validation, "Sheet1", style = numeric_with_decimals_style, rows = 2:(num_rows + 1), cols = 8)

addStyle(wb_summary_validation, "Sheet1", style = numeric_with_decimals_style, rows = 2:(num_rows + 1), cols = 9)

addStyle(wb_summary_validation, "Sheet1", style = numeric_with_decimals_style, rows = 2:(num_rows + 1), cols = 10)

addStyle(wb_summary_validation, "Sheet1", style = numeric_with_decimals_style, rows = 2:(num_rows + 1), cols = 11)

# Set column widths.

setColWidths(wb_summary_validation, sheet = "Sheet1", cols = 1, widths = "20")

setColWidths(wb_summary_validation, sheet = "Sheet1", cols = 2, widths = "20")

setColWidths(wb_summary_validation, sheet = "Sheet1", cols = 3, widths = "20")

setColWidths(wb_summary_validation, sheet = "Sheet1", cols = 4, widths = "20")

setColWidths(wb_summary_validation, sheet = "Sheet1", cols = 5, widths = "20")
```

```
setColWidths(wb_summary_validation, sheet = "Sheet1", cols = 6, widths =
     "20")

setColWidths(wb_summary_validation, sheet = "Sheet1", cols = 7, widths =
     "42")

setColWidths(wb_summary_validation, sheet = "Sheet1", cols = 8, widths =
     "20")

setColWidths(wb_summary_validation, sheet = "Sheet1", cols = 9, widths =
     "20")

setColWidths(wb_summary_validation, sheet = "Sheet1", cols = 10, widths
    = "20")

setColWidths(wb_summary_validation, sheet = "Sheet1", cols = 11, widths
    = "20")

# Save the resulting Excel workbook.

saveWorkbook(wb_summary_validation, "R_Objects/ThemesValidation/
    Validation_Summary_Themes.xlsx", overwrite = FALSE)
```

Clean up the global environment.

Listing 11.14: Cleaning up the Global Environment After Validation

```
# Save the objects needed for later steps and/or for auditing our work.

saveRDS(GiftedFictionThemes, "R_Objects/ThemesValidation/
    GiftedFictionThemes.rds")

saveRDS(dictionary_list, "R_Objects/ThemesValidation/dictionary_list.rds
    ")

saveRDS(final_results, "R_Objects/ThemesValidation/final_results.rds")
```

```r
saveRDS(final_results_df, "R_Objects/ThemesValidation/final_results_df.rds")
saveRDS(final_results_df_with_sums, "R_Objects/ThemesValidation/final_results_df_with_sums.rds")
saveRDS(text_lengths, "R_Objects/ThemesValidation/text_lengths.rds")
saveRDS(final_results_sums_df, "R_Objects/ThemesValidation/final_results_sums_df.rds")
saveRDS(summary_results_themes, "R_Objects/ThemesValidation/summary_results_themes.rds")
# Retrieve the names of all objects in the global environment and store them in a character vector.
all_objects <- ls()
# Create a character vector of object names in the global environment.
formatted_object_array <- paste('c("', paste(all_objects, collapse = '", "'), '")', sep = "")
# Export a formatted list to a .txt file for manual review.
write(formatted_object_array, "R_objects.txt")
# Manually review the .txt file and delete objects to be kept in the R global environment.
# Import the first line of the modified .txt file back into R.
string_of_object_names <- readLines("R_objects.txt", n = 1)
# Convert the string in the .txt file back into a character vector of object names.
formatted_object_array <- eval(parse(text = string_of_object_names))
# Remove the objects selected for removal from the global environment.
```

```
rm(list = c(formatted_object_array))

rm(list = c("all_objects", "formatted_object_array", "string_of_object_
    names"))

# Invoke the garbage collection function gc() to release memory occupied
     by no-longer-used objects and to compact the storage of remaining
    objects in the global environment to improve memory usage.

gc()
```

12 Sentiment Analysis

Subject the *gift** and *only* passages to sentiment analysis.

First, load the non-lemmatized tokens object `gift_only_tokens51` from the RDS file back into the global environment.

Listing 12.1: Loading the Non-Lemmatized Tokens Object

```
gift_only_tokens51 <- readRDS("R_Objects/Corpora/gift_only_tokens51.rds"
    )
```

Second, take note of the Lexicoder Sentiment Dictionary as included in Quanteda [4, 37]. It should already be loaded along with the quanteda package as `data_dictionary_LSD2015`.

Listing 12.2: Taking Note of the Lexicoder Sentiment Dictionary

```
class(data_dictionary_LSD2015)

```

```
str(data_dictionary_LSD2015)
```

Third, create a function for converting the glob patterns [3] used in the Lexicoder Sentiment Dictionary to the regular-expression (regex) patterns needed for the sentiment analysis (below).

Listing 12.3: Converting Glob Patterns

```
glob_to_regex <- function(glob) {

  first_char <- ifelse(substr(glob, nchar(glob), nchar(glob)) == "*", "", "$")

  paste0("^", gsub("\\*", "", glob), first_char)

}
```

Fourth, extract and count positive sentiments.

Start by extracting positive sentiments from the Lexicoder Sentiment Dictionary, converting the positive sentiment word tokens to regex patterns with the `glob_to_regex()` function from Listing 12.3, and creating a function using the `grep()` function to find positive sentiment matches in documents.

Listing 12.4: Preparing for the Positive Sentiment Analysis

```
positive_sentiments <- data_dictionary_LSD2015$positive

positive_patterns <- sapply(positive_sentiments, glob_to_regex)

find_sentiment_matches <- function(doc_tokens) {

  grep(paste(positive_patterns, collapse = "|"), doc_tokens)

```

```
}
```

Use the `lapply()` function to find matches with positive sentiment tokens across all documents. The resulting object contains the index locations of the matched positive sentiment tokens. Save the object outside or R for later use.

Listing 12.5: Finding Positive Sentiment Tokens

```
positive_sentiment_index_locations_l <- lapply(gift_only_tokens51, find_
    sentiment_matches)
```

Calculate the relative frequency of positive sentiments in the `positive_sentiment_index_locations_l` object.

Listing 12.6: Calculating the Relative Frequency of Positive Sentiments

```
# Count the number of positive sentiments per document.

positive_sentiment_passage_counts_v <- lengths(positive_sentiment_index_
    locations_l)

# Count the tokens per document.

token_counts_per_document51 <- sapply(gift_only_tokens51, length)

# Divide the positive sentiment counts by the token counts.

relative_frequency_pos_sentiments <- (positive_sentiment_passage_counts_
    v / token_counts_per_document51)
```

Fifth, extract and count negative sentiments.

Start by extracting negative sentiments from the Lexicoder Sentiment Dictionary, converting the negative sentiment word tokens to regex patterns with the `glob_to_regex()` function from Listing 12.3, and creating a function using the `grep()` function to find negative sentiment matches in documents.

Listing 12.7: Preparing for the Negative Sentiment Analysis

```
negative_sentiments <- data_dictionary_LSD2015$negative

negative_patterns <- sapply(negative_sentiments, glob_to_regex)

# The resulting vector of negative sentiment tokens ('negative_patterns
    ') is too long for the grep() function given the R memory limits of
    our R environment. For that reason, we suggest dividing it into 3
    vectors of roughly equal length.

n <- length(negative_patterns)

group_sizes <- c(floor(n/3), floor(n/3), n - 2*floor(n/3))

group_indices <- rep(1:3, times = group_sizes)

split_patterns <- split(negative_patterns, f = group_indices)

str(split_patterns)

# Create a function using the grep() function to find negative sentiment
     matches that also accounts for the list of negative sentiments being
     split into three shorter lists.

find_neg_sentiment_matches_for_subset_patterns <- function(doc_tokens,
    subset_of_patterns) {

  grep(paste(subset_of_patterns, collapse = "|"), doc_tokens)

}
```

Use the `lapply()` function to find matches with negative sentiment tokens across all documents. The resulting object contains the index locations of the matched negative sentiment tokens. Save the object outside or R for later use.

Listing 12.8: Finding Negative Sentiment Tokens

```
negative_sentiment_index_locations_lists <- lapply(split_patterns,
    function(pattern_subset) {

  lapply(gift_only_tokens51, find_neg_sentiment_matches_for_subset_
     patterns, subset_of_patterns = pattern_subset)

})

# Combine the results of all three pattern subsets into one list (
    analogous to the one for positive sentiments).

negative_sentiment_index_locations_l <- lapply(seq_along(gift_only_
    tokens51), function(doc_idx) {

  unique(unlist(lapply(negative_sentiment_index_locations_lists,
     function(lst) lst[[doc_idx]])))

})
```

Calculate the relative frequency of negative sentiments in the `negative_sentiment_index_locations_l` object.

Listing 12.9: Calculating the Relative Frequency of Negative Sentiments

```
# Count the number of negative sentiments per document.

negative_sentiment_passage_counts_v <- lengths(negative_sentiment_index_
    locations_l)

# We need the number of tokens per document. We have already counted
    this, above, and stored it in the 'token_counts_per_document51'
    object.

```

```
# Divide the negative sentiment counts by the token counts.

relative_frequency_neg_sentiments <- (negative_sentiment_passage_counts_
    v / token_counts_per_document51)
```

Sixth, create a data frame with the positive and negative sentiment count information for comparative analyses.

Listing 12.10: Collecting the Sentiment Count Information

```
# Extract the SampleType docvar.

sample_types <- attr(gift_only_tokens51, "docvars")$SampleType

# Create a data frame for the results using the following labels for the
     sentiment variables: AFPS = absolute frequency of positive
    sentiments; RFPS = relative frequency of positive sentiments; AFNS =
    absolute frequency of negative sentiments; and RFNS = relative
    frequency of negative sentiments.

sentiment_results_df <- data.frame(

  SampleType = sample_types,

  Tokens = token_counts_per_document51,

  AFPS = positive_sentiment_passage_counts_v,

  RFPS = relative_frequency_pos_sentiments,

  AFNS = negative_sentiment_passage_counts_v,

  RFNS = relative_frequency_neg_sentiments

)

# Check the results.
```

```
str(sentiment_results_df)

head(sentiment_results_df)

tail(sentiment_results_df)

View(sentiment_results_df)
```

Seventh, compare the sample types regarding the prevalence of positive/negative sentiment tokens (using the relative frequency of positive/negative sentiments).

Listing 12.11: Comparing Negative and Positive Sentiments by Sample Type

```
# Extract the sentiment frequency information by sample type.

RFPS_gift <- sentiment_results_df$RFPS[sentiment_results_df$SampleType == "gift"]

RFPS_only <- sentiment_results_df$RFPS[sentiment_results_df$SampleType == "only"]

RFNS_gift <- sentiment_results_df$RFNS[sentiment_results_df$SampleType == "gift"]

RFNS_only <- sentiment_results_df$RFNS[sentiment_results_df$SampleType == "only"]

# Describe the data using the describe() function from the psych package.

desc_RFPS_gift <- as.data.frame(describe(RFPS_gift))

desc_RFNS_gift <- as.data.frame(describe(RFNS_gift))

desc_RFPS_only <- as.data.frame(describe(RFPS_only))

```

```
desc_RFNS_only <- as.data.frame(describe(RFNS_only))

# Add columns with names for each variable.

desc_RFPS_gift$Description <- "Positive sentiments (gift)"

desc_RFNS_gift$Description <- "Negative sentiments (gift)"

desc_RFPS_only$Description <- "Positive sentiments (only)"

desc_RFNS_only$Description <- "Negative sentiments (only)"

# Bind together the resulting data frames.

Descriptives_RFs <- bind_rows(desc_RFPS_gift, desc_RFNS_gift, desc_RFPS_
    only, desc_RFNS_only)

# Move the names variable to the left-most column.

Descriptives_RFs <- Descriptives_RFs %>%

  select(Description, everything())

# Delete the vars column, which is a variable count in the psych package
     describe() function output that we don't need.

Descriptives_RFs <- Descriptives_RFs %>%

  select(-vars)

# Check the output.

str(Descriptives_RFs)

View(Descriptives_RFs)
```

Eighth, export the `Descriptives_RFs` object to Excel, using the openxlsx package [28] to allow for pre-defining the format of the Excel columns.

Listing 12.12: Exporting the `Descriptives_RFs` Object to Excel

```
# Create a new workbook for the impending Excel import.

DescriptivesWb <- createWorkbook()

# Add a worksheet to the workbook.

addWorksheet(DescriptivesWb, "Sheet1")

# Create custom column format styles for the Excel export via openxlsx.

header_style <- createStyle(fontName = "Calibri", fontColour = "black", textDecoration = "bold")

char_style <- createStyle(numFmt = "@") # This is for text data.

numeric_with_decimals_style <- createStyle(numFmt = "0.000") # This allows for three digits after the decimal point.

whole_numbers_style <- createStyle(numFmt = "0") # Needed for whole numbers.

# Export the data frame to the worksheet.

writeData(DescriptivesWb, "Sheet1", Descriptives_RFs)

# Define the number of rows to be formatted in the worksheet.

num_rows <- nrow(Descriptives_RFs)

# Set the column width to 25 units for column 1.

setColWidths(DescriptivesWb, sheet = "Sheet1", cols = 1, widths = "25")

# Set the column widths to 16 units for columns 2-13.

setColWidths(DescriptivesWb, sheet = "Sheet1", cols = 2:13, widths = "16")

```

```
# Apply the column format styles to specific columns.

addStyle(DescriptivesWb, "Sheet1", style = header_style, rows = 1, cols
    = 1:13)

addStyle(DescriptivesWb, "Sheet1", style = char_style, rows = 2:(num_
    rows + 1), cols = 1)

# Apply 'numeric_with_decimals_style' with repetitions via a loop. The
    loop is needed because the createWorkbook() function does not allow a
     setting to be applied to different ranges of rows and columns. This
    is because the openxlsx package maps the styles to the cells via
    Cartesian mapping.

for (col_num in 3:13) {

  addStyle(DescriptivesWb, "Sheet1", style = numeric_with_decimals_style
     , rows = 2:(num_rows + 1), cols = col_num)

}

addStyle(DescriptivesWb, "Sheet1", style = whole_numbers_style, rows =
    2:(num_rows + 1), cols = 2)

# Save the resulting Excel workbook.

saveWorkbook(DescriptivesWb, "R_Objects/Sentiments/Descriptives_RFs.xlsx
    ", overwrite = FALSE)
```

Ninth, test the values we intend to compare for normality with the Kolmogorov–Smirnov Test with Lilliefors Correction for each group (used due to the large sample size instead of the Shapiro–Wilk Test). For simplicity's sake, send the outputs directly into locally saved .txt files for later review and reporting.

Listing 12.13: Testing the Sentiment Data for Normality

```
sink("R_Objects/Sentiments/lillie.test_RFPS_gift.txt", append = TRUE)

```

```
lillie.test(RFPS_gift)

sink()

sink("R_Objects/Sentiments/lillie.test_RFPS_only.txt", append = TRUE)

lillie.test(RFPS_only)

sink()

sink("R_Objects/Sentiments/lillie.test_RFNS_gift.txt", append = TRUE)

lillie.test(RFNS_gift)

sink()

sink("R_Objects/Sentiments/lillie.test_RFNS_only.txt", append = TRUE)

lillie.test(RFNS_only)

sink()
```

Tenth, test for equality of variances for each group with Levene's Test. For simplicity's sake, send the outputs directly into locally saved .txt files for later review and reporting.

Listing 12.14: Testing the Sentiment Data for Equality of Variances

```
sink("R_Objects/Sentiments/leveneTest_RFPS.txt", append = TRUE)

leveneTest(sentiment_results_df$RFPS ~ sentiment_results_df$SampleType)

sink()

sink("R_Objects/Sentiments/leveneTest_RFNS.txt", append = TRUE)

leveneTest(sentiment_results_df$RFNS ~ sentiment_results_df$SampleType)

```

```
sink()
```

Eleventh, compare the means with Welch's *t* Test (a parametric test that does not require equal variances) as well as with the Mann–Whitney *U* Test (a non-parametric test that requires neither equal variances nor normal distributions). The parametric test should be sufficient despite the violation of normality in light of the large sample sizes. The Mann–Whitney *U* Test adds additional confidence that the test statistic is not merely reflecting artifacts of the data. Then assess the effect sizes of the differences with Cohen's *d* as well as with Cliff's delta, which does not require a specific shape or spread of the distribution.

Listing 12.15: Comparing Means in the Sentiment Data

```
# Initialize a tibble to store summary results.
summary_results_sentiments <- tibble()
# Create a list of normalized sum variables to test.
norm_sentiments <- c("RFPS", "RFNS")
# Set the names of the variables.
theme_map <- list(
  RFPS = "Relative␣Frequency␣of␣Positive␣Sentiments",
  RFNS = "Relative␣Frequency␣of␣Negative␣Sentiments"
)
# Loop through each normalized sum variable, effecting the needed tests and measurements: Mann-Whitney U tests, Welch's t tests, means, Cohen's d, and Cliff's delta.
for (var in norm_sentiments) {
  # Extract the specific variable and sample type from the data frame.
```

```r
specific_var <- sentiment_results_df[[var]]

sample_type <- sentiment_results_df$SampleType

# Run the Mann-Whitney U tests.

mw_test_result <- wilcox.test(specific_var ~ sample_type)

# Run Welch's t tests.

welch_test_result <- t.test(specific_var ~ sample_type, var.equal = FALSE)

# Calculate the means for the 'gift*' and 'only' groups.

mean_gift <- mean(specific_var[sample_type == 'gift'], na.rm = TRUE)

mean_only <- mean(specific_var[sample_type == 'only'], na.rm = TRUE)

# Calculate Cohen's d.

cohens_d_result <- cohen.d(specific_var ~ sample_type)

# Calculate Cliff's delta.

cliffs_delta_result <- cliff.delta(specific_var[sample_type == 'gift'], specific_var[sample_type == 'only'])

# Map a variable prefix to a variable name if the variable name is "RFPS" or "RFNS". Integrate an if-else loop to make sure we are only using the desired normalized variables in the 'final_results_sums_df' object.

# Define the combined pattern.

pattern <- "RFPS|RFNS"

if (grepl(pattern, var)) {

  theme_abbreviation <- var

  if (theme_abbreviation %in% names(theme_map)) {
```

```r
      theme <- theme_map[[theme_abbreviation]]
      # Create a tibble row with the calculated and tested data outputs.
      row_tibble <- tibble(
        Sentiment = theme,
        `Mean ('gift*')` = mean_gift,
        `Mean ('only')` = mean_only,
        Welch = welch_test_result$statistic,
        `Welch df` = welch_test_result$parameter,
        `Welch p` = welch_test_result$p.value,
        `Welch 95% CI` = paste(welch_test_result$conf.int[1], "to", welch_test_result$conf.int[2]),
        U = mw_test_result$statistic,
        `Mann-Whitney p` = mw_test_result$p.value,
        `Cohens d` = cohens_d_result$estimate,
        `Cliffs d` = cliffs_delta_result$estimate
      )
      # Add the new row to the summary tibble.
      summary_results_sentiments <- bind_rows(summary_results_sentiments, row_tibble)
    } else {
      warning(paste("Theme abbreviation not found in theme_map:", theme_abbreviation))
```

```
    }

  }

}

# Print out the summary tibble.

print(summary_results_sentiments)
```

Twelfth, export the `summary_results_sentiments` object to Excel, using the openxlsx package [28] to allow for pre-defining the format of the Excel columns.

Listing 12.16: Exporting the Sentiment results to Excel

```
# Create a new workbook for the impending Excel import.

wb_summary_sentiments <- createWorkbook()

# Add a worksheet to the workbook.

addWorksheet(wb_summary_sentiments, "Sheet1")

# Create custom column format styles for the Excel export via openxlsx.

header_style <- createStyle(fontName = "Calibri", fontColour = "black",
    textDecoration = "bold")

char_style <- createStyle(numFmt = "@") # This is for text data.

numeric_with_decimals_style <- createStyle(numFmt = "0.000") # This
    allows for three digits after the decimal point.

# Export the data frame to the worksheet.

writeData(wb_summary_sentiments, "Sheet1", summary_results_sentiments)

```

```
# Define the number of rows to be formatted in the worksheet.

num_rows <- nrow(summary_results_sentiments)

# Apply the column format styles to specific columns.

addStyle(wb_summary_sentiments, "Sheet1", style = header_style, rows =
    1, cols = 1:11)

addStyle(wb_summary_sentiments, "Sheet1", style = char_style, rows = 2:(
    num_rows + 1), cols = 1)

addStyle(wb_summary_sentiments, "Sheet1", style = numeric_with_decimals_
    style, rows = 2:(num_rows + 1), cols = 2)

addStyle(wb_summary_sentiments, "Sheet1", style = numeric_with_decimals_
    style, rows = 2:(num_rows + 1), cols = 3)

addStyle(wb_summary_sentiments, "Sheet1", style = numeric_with_decimals_
    style, rows = 2:(num_rows + 1), cols = 4)

addStyle(wb_summary_sentiments, "Sheet1", style = numeric_with_decimals_
    style, rows = 2:(num_rows + 1), cols = 5)

addStyle(wb_summary_sentiments, "Sheet1", style = numeric_with_decimals_
    style, rows = 2:(num_rows + 1), cols = 6)

addStyle(wb_summary_sentiments, "Sheet1", style = numeric_with_decimals_
    style, rows = 2:(num_rows + 1), cols = 7)

addStyle(wb_summary_sentiments, "Sheet1", style = numeric_with_decimals_
    style, rows = 2:(num_rows + 1), cols = 8)

addStyle(wb_summary_sentiments, "Sheet1", style = numeric_with_decimals_
    style, rows = 2:(num_rows + 1), cols = 9)

addStyle(wb_summary_sentiments, "Sheet1", style = numeric_with_decimals_
    style, rows = 2:(num_rows + 1), cols = 10)

addStyle(wb_summary_sentiments, "Sheet1", style = numeric_with_decimals_
    style, rows = 2:(num_rows + 1), cols = 11)
```

```
# Set the column widths.

setColWidths(wb_summary_sentiments, sheet = "Sheet1", cols = 1, widths =
    "35")

setColWidths(wb_summary_sentiments, sheet = "Sheet1", cols = 2, widths =
    "20")

setColWidths(wb_summary_sentiments, sheet = "Sheet1", cols = 3, widths =
    "20")

setColWidths(wb_summary_sentiments, sheet = "Sheet1", cols = 4, widths =
    "20")

setColWidths(wb_summary_sentiments, sheet = "Sheet1", cols = 5, widths =
    "20")

setColWidths(wb_summary_sentiments, sheet = "Sheet1", cols = 6, widths =
    "20")

setColWidths(wb_summary_sentiments, sheet = "Sheet1", cols = 7, widths =
    "42")

setColWidths(wb_summary_sentiments, sheet = "Sheet1", cols = 8, widths =
    "20")

setColWidths(wb_summary_sentiments, sheet = "Sheet1", cols = 9, widths =
    "20")

setColWidths(wb_summary_sentiments, sheet = "Sheet1", cols = 10, widths
   = "20")

setColWidths(wb_summary_sentiments, sheet = "Sheet1", cols = 11, widths
   = "20")

# Save the resulting Excel workbook.

saveWorkbook(wb_summary_sentiments, "R_Objects/Sentiments/Sentiments_
   Summary.xlsx", overwrite = FALSE)
```

Clean up the global environment.

Listing 12.17: Cleaning up the Global Environment After Sentiment Analysis

```
# Save the objects needed for later steps and/or for auditing our work.

saveRDS(positive_sentiment_index_locations_l, "R_Objects/Sentiments/positive_sentiment_index_locations_l.rds")

saveRDS(positive_sentiment_passage_counts_v, "R_Objects/Sentiments/positive_sentiment_passage_counts_v.rds")

saveRDS(token_counts_per_document51, "R_Objects/Sentiments/token_counts_per_document51.rds")

saveRDS(relative_frequency_pos_sentiments, "R_Objects/Sentiments/relative_frequency_pos_sentiments.rds")

saveRDS(negative_sentiment_index_locations_lists, "R_Objects/Sentiments/negative_sentiment_index_locations_lists.rds")

saveRDS(negative_sentiment_index_locations_l, "R_Objects/Sentiments/negative_sentiment_index_locations_l.rds")

saveRDS(negative_sentiment_passage_counts_v, "R_Objects/Sentiments/negative_sentiment_passage_counts_v.rds")

saveRDS(relative_frequency_neg_sentiments, "R_Objects/Sentiments/relative_frequency_neg_sentiments.rds")

saveRDS(sample_types, "R_Objects/Sentiments/sample_types.rds")

saveRDS(sentiment_results_df, "R_Objects/Sentiments/sentiment_results_df.rds")

saveRDS(Descriptives_RFs, "R_Objects/Sentiments/Descriptives_RFs.rds")

# Retrieve the names of all objects in the global environment and store them in a character vector.

all_objects <- ls()

```

```r
# Create a character vector of object names in the global environment.

formatted_object_array <- paste('c("', paste(all_objects, collapse = '", "'), '")', sep = "")

# Export a formatted list to a .txt file for manual review.

write(formatted_object_array, "R_objects.txt")

# Manually review the .txt file and delete objects to be kept in the R global environment.

# Import the first line of the modified .txt file back into R.

string_of_object_names <- readLines("R_objects.txt", n = 1)

# Convert the string in the .txt file back into a character vector of object names.

formatted_object_array <- eval(parse(text = string_of_object_names))

# Remove the objects selected for removal from the global environment.

rm(list = c(formatted_object_array))

rm(list = c("all_objects", "formatted_object_array", "string_of_object_names"))

# Invoke the garbage collection function gc() to release memory occupied by no-longer-used objects and to compact the storage of remaining objects in the global environment to improve memory usage.

gc()
```

13 Additional Operations for Manuscript Preparation

This section includes code we compiled—while writing up our main manuscript—that was not part of the code pipeline constituting the quantitative text analysis described in the manuscript.

We required an example of a passage before, during, and after preprocessing (for Table 1 in the main manuscript).

Use text 4 (text4), for example, but do not use text 3. Text 3 has a noted omission due to copyright restrictions (noted in the main manuscript), which makes it less helpful for illustrative purposes.

First, load the required objects into the global environment and subset the final object as needed for the example described in Table 1 of the main manuscript.

Listing 13.1: Preparing the Example Extraction Process

```
doc_lengths_gift_dfm51_34 <- readRDS("R_Objects/PCA/doc_lengths_gift_
    dfm51_34.rds")

gift_dfm51_34 <- readRDS("R_Objects/PCA/gift_dfm51_34.rds")

gift_only_corpus51 <- readRDS("R_Objects/Corpora/gift_only_corpus51.rds"
    )

gift_only_tokens51 <- readRDS("R_Objects/Corpora/gift_only_tokens51.rds"
    )

gift_only_tokens_lemmatized51 <- readRDS("R_Objects/Corpora/gift_only_
    tokens_lemmatized51.rds")

gift_tokens_lemmatized51 <- tokens_subset(gift_only_tokens_lemmatized51,
     SampleType == "gift")
```

Second, calculate deciles for the distribution of token lengths in the *gift** passages (referred to also as 'documents') after they were filtered for the principal component analysis (PCA). The lengths are stored in lstinlinedoc_lengths_gift_dfm51_34. We will use the deciles to characterize where our example falls within the length distribution of the filtered passages examined in the PCA.

Listing 13.2: Calculating Deciles

```
deciles <- quantile(doc_lengths_gift_dfm51_34, probs = seq(0.1, 1, by =
    0.1))

```

```
print(deciles)
```

Third, find the token length of text 4.

Listing 13.3: Finding the Token Length of Text 4

```
length_text4 <- doc_lengths_gift_dfm51_34["text4"]
```

Fourth, determine in which text-length decile text4 falls.

Listing 13.4: Determining the Decile of Text 4

```
decile_text4 <- sum(length_text4 > deciles) + 1

print(decile_text4)
```

According to `decile_text4`, text 4 falls into the eighth decile for length in the document–feature matrix prepared for the PCA. With five word tokens, it is among the longer texts. The length distribution of the document–feature matrix prepared for the PCA is described in the main manuscript.

Fifth, extract text 4 from the original, not-yet-pre-processed corpus object.

Listing 13.5: Extracting From the Unprocessed Corpus

```
text4_1 <- gift_only_corpus51[4,]

Print the entire content of text4_1 without truncation.
```

```

cat(text4_1, sep = "\n")
```

Sixth, extract text 4 from the pre-processed, but not yet lemmatized tokens object.

Listing 13.6: Extracting From the Partially Processed Corpus

```
text4_2 <- gift_only_tokens51[4,]

# Convert the tokens object to a character string by joining the tokens.

text4_2_text <- paste(text4_2, collapse = "␣")

# Print the entire content of text4_2 without truncation.

cat(text4_2_text, sep = "\n")
```

Seventh, extract text 4 from the pre-processed, lemmatized tokens object.

Listing 13.7: Extracting From the Lemmatized Corpus

```
text4_3 <- gift_tokens_lemmatized51[4,]

# Convert the tokens object to a character string by joining the tokens.

text4_3_text <- paste(text4_3, collapse = "␣")

# Print the entire content of 'text4_3' without truncation.

cat(text4_3_text, sep = "\n")
```

Eighth, extract text 4 from the document–feature matrix used for the PCA. As described in Listing **??** and

more conceptually in the main manuscript, this object was restricted to word types that occur in at least 35 of the 2,104 texts that make up the *gift** corpus.

Listing 13.8: Extracting From the Filtered Corpus

```
text4_4 <- gift_dfm51_34[4,]

# Extract non-zero features from the document-feature matrix for the
    document 'text4_4'. To this end, start by identifying columns with
    non-zero values.

non_zero_cols <- colSums(text4_4) > 0

# Now subset the document-feature matrix to include only non-zero
    columns.

non_zero_features <- text4_4[, non_zero_cols]

# Convert the non-zero features to a data frame for easier viewing.

non_zero_df <- convert(non_zero_features, to = "data.frame")

# Print the non-zero features and their counts.

print(non_zero_df)
```

We also needed to describe the false-discovery-rate-corrected p value for the keyword ‘genius’ in terms of sigma (presupposing a standard normal distribution). To this end, convert the FDR-corrected two-tailed p value to a one-tailed p value.

Listing 13.9: Calculating Sigma for the FDR-Corrected p Value for ‘genius’

```
# Load the keywords object.

gift_only_keywords51_significant <- readRDS("R_Objects/Keywords/gift_
```

```
    only_keywords51_significant.rds")

# Find the position of 'genius' in the 'feature' vector.

print(gift_only_keywords51_significant$feature)

# Extract the 19th value in the vector false-discovery-rate-corrected p
    values.

str(gift_only_keywords51_significant)

FDR_P_Genius <- gift_only_keywords51_significant[19,4]

# Convert the FDR-corrected two-tailed p value to a one-tailed p-value.

FDR_P_Genius_1t <- FDR_P_Genius / 2

# Find the z-score (standard deviations from the mean).

Genus_z_score <- qnorm(1 - FDR_P_Genius_1t)

cat("The␣z-score␣corresponding␣to␣a␣two-tailed␣p-value␣of", FDR_P_Genius
    , "is", round(Genus_z_score, 11), "standard␣deviations␣from␣the␣mean
    .\n")
```

Clean up the global environment.

Listing 13.10: Cleaning up the Global Environment After the Additional Operations

```
# Retrieve the names of all objects in the global environment and store
    them in a character vector.

all_objects <- ls()

# Create a character vector of object names in the global environment.

formatted_object_array <- paste('c("', paste(all_objects, collapse = '",
```

```
␣"'), '")', sep = "")

# Export a formatted list to a .txt file for manual review.

write(formatted_object_array, "R_objects.txt")

# Manually review the .txt file and delete objects to be kept in the R global environment.

# Import the first line of the modified .txt file back into R.

string_of_object_names <- readLines("R_objects.txt", n = 1)

# Convert the string in the .txt file back into a character vector of object names.

formatted_object_array <- eval(parse(text = string_of_object_names))

# Remove the objects selected for removal from the global environment.

rm(list = c(formatted_object_array))

rm(list = c("all_objects", "formatted_object_array", "string_of_object_names"))

# Invoke the garbage collection function gc() to release memory occupied by no-longer-used objects and to compact the storage of remaining objects in the global environment to improve memory usage.

gc()
```

References

[1] ab0rt. *How to convert a tokens object into a corpus object*. Part of R Language Collective. Stack Overflow. Oct. 2021. URL: https://stackoverflow.com/questions/69591928/how-to-convert-a-tokens-object-into-a-corpus-object (visited on 12/03/2023).

[2] Auguie Baptiste. *gridExtra: Miscellaneous Functions for "Grid" Graphics*. R package version 2.3. 2017. URL: https://CRAN.R-project.org/package=gridExtra.

[3] Bell Laboratories. *Unix Glob Reference*. Online image. Accessed on: 8 December 2023. 1971. URL: https://commons.wikimedia.org/wiki/File:Unix_Glob_Reference.png.

[4] Kenneth Benoit and other contributors. *Lexicoder Sentiment Dictionary (2015)*. R package version 3.3.1. 2015. URL: https://search.r-project.org/CRAN/refmans/quanteda/html/data_dictionary_LSD2015.html.

[5] Kenneth Benoit and Akitaka Matsuo. *spacyr: Wrapper to the 'spaCy' 'NLP' Library*. R package version 1.3.0. 2023. URL: https://spacyr.quanteda.io.

[6] Kenneth Benoit and Adam Obeng. *readtext: Import and Handling for Plain and Formatted Text Files*. R package version 0.90. 2023. URL: https://CRAN.R-project.org/package=readtext.

[7] Kenneth Benoit et al. *Count the Number of Tokens or Types — ntoken*. Accessed on: 23 September 2023. 2023. URL: https://quanteda.io/reference/ntoken.html.

[8] Kenneth Benoit et al. "quanteda: An R Package for the Quantitative Analysis of Textual Data." In: *Journal of Open Source Software* 3.30 (2018), p. 774. DOI: 10.21105/joss.00774. URL: https://quanteda.io.

[9] Kenneth Benoit et al. *quanteda.textstats: Textual Statistics for the Quantitative Analysis of Textual Data*. en-GB. R package version 0.96.4. European Research Council. London, United Kingdom, 2023. URL: https://CRAN.R-project.org/package=quanteda.textstats.

[10] Ryan L. Boyd. "Psychological text analysis in the digital humanities." In: *Data Analytics in Digital Humanities*. Ed. by Shalin Hai-Jew. Cham: Springer International Publishing, 2017, pp. 161–189.

[11] John M. Chambers. *Graphical Methods for Data Analysis*. English. Boston, MA: Duxbury Press, 1983, pp. XIV, 395. ISBN: 9780534980528.

[12] *Dictionary function documentation in Quanteda*. Accessed on: 7 December 2023. 2023. URL: https://quanteda.io/reference/dictionary.html.

[13] Ingo Feinerer, Kurt Hornik, and Mike Wallace. *WordNet Interface*. Accessed on: 4 January 2024. CRAN. Jan. 2023. URL: https://cran.r-project.org/web/packages/wordnet/wordnet.pdf.

[14] Christiane Fellbaum, ed. *WordNet: An Electronic Lexical Database*. Cambridge, MA: MIT Press, 1998.

[15] John Fox and Sanford Weisberg. *An R Companion to Applied Regression*. Third. Thousand Oaks CA: Sage, 2019. URL: https://socialsciences.mcmaster.ca/jfox/Books/Companion/.

[16] *Full-Text Corpus Data Licensing*. Available from: Corpus Data Organization. Accessed on 18 February 2024. 2024. URL: https://www.corpusdata.org/purchase.asp.

[17] Juergen Gross and Uwe Ligges. *nortest: Tests for Normality*. R package version 1.0-4. 2015. URL: https://CRAN.R-project.org/package=nortest.

[18] J. L. Horn. "A rationale and test for the number of factors in factor analysis." In: *Psychometrika* 30.2 (1965), pp. 179–185. DOI: 10.1007/bf02289447.

[19] Raivo Kolde. *Pretty Heatmaps*. Accessed on: 4 January 2024. CRAN. Oct. 2022. URL: https://cran.r-project.org/web/packages/pheatmap/pheatmap.pdf.

[20] David D. Lewis et al. "RCV1: A New Benchmark Collection for Text Categorization Research." In: *Journal of Machine Learning Research* 5 (2004), pp. 361–397.

[21] David M. Markowitz. "The meaning extraction method. An approach to evaluate content patterns from large-scale language data." In: *Front. Commun.* 6 (2021), Article 588823. DOI: 10.3389/fcomm.2021.588823.

[22] Merriam-Webster. *Gift – Merriam-Webster's Dictionary*. https://www.merriam-webster.com/dictionary/gift. Accessed on: 25 September 2023. n.d.

[23] Jeroen Ooms. *writexl: Export Data Frames to Excel 'xlsx' Format*. R package version 1.4.2. 2023. URL: https://CRAN.R-project.org/package=writexl.

[24] Oracle. *Java Downloads*. Accessed on: 4 January 2024. 2024. URL: %5Cspecialurl%7Bhttps://www.oracle.com/java/technologies/downloads/#jdk21-windows%7D.

[25] Paul Poncet. *modeest: Mode Estimation*. R package version 2.4.0. 2019. URL: https://CRAN.R-project.org/package=modeest.

[26] Princeton University. *About WordNet: WordNet*. Accessed on: 4 January 2024. 2010. URL: https://wordnet.princeton.edu/.

[27] William Revelle. *psych: Procedures for Psychological, Psychometric, and Personality Research*. R package version 2.3.6. Northwestern University. Evanston, Illinois, 2023. URL: https://CRAN.R-project.org/package=psych.

[28] Philipp Schauberger and Alexander Walker. *openxlsx: Read, Write and Edit xlsx Files*. R package version 4.2.5.2. 2023. URL: https://CRAN.R-project.org/package=openxlsx.

[29] R Core Team. *data_stopwords_smart {stopwords}*. n.d. URL: https://search.r-project.org/CRAN/refmans/stopwords/html/data_stopwords_smart.html (visited on 12/02/2023).

[30] Marco Torchiano. *effsize: Efficient Effect Size Computation*. R package version 0.8.1. 2020. DOI: 10.5281/zenodo.1480624. URL: https://CRAN.R-project.org/package=effsize.

[31] Simon Urbanek. *Low-Level R to Java Interface*. Version 1.0-10. R package version 1.0-10, License LGPL-2.1. CRAN. Dec. 2023. URL: https://cran.r-project.org/web/packages/rJava/rJava.pdf.

[32] Kevin Ushey, JJ Allaire, and Yuan Tang. *reticulate: Interface to 'Python'*. R package version 1.30. 2023. URL: https://CRAN.R-project.org/package=reticulate.

[33] Hadley Wickham. *stringr: Simple, Consistent Wrappers for Common String Operations*. R package version 1.5.0. 2022. URL: https://CRAN.R-project.org/package=stringr.

[34] Hadley Wickham et al. *devtools: Tools to Make Developing R Packages Easier*. R package version 2.4.5. 2022. URL: https://CRAN.R-project.org/package=devtools.

[35] Hadley Wickham et al. *Scale Functions for Visualization*. R package version 1.3.0, https://github.com/r-lib/scales. Posit, PBC. CRAN, Nov. 2023. URL: https://scales.r-lib.org.

[36] Hadley Wickham et al. "Welcome to the tidyverse." In: *Journal of Open Source Software* 4.43 (2019), p. 1686. DOI: 10.21105/joss.01686. URL: https://doi.org/10.21105/joss.01686.

[37] Lori Young and Stuart Soroka. "Affective news: The automated coding of sentiment in political texts." In: *Political Communication* 29.2 (2012), pp. 205–231. URL: https://doi.org/10.1080/10584609.2012.671234.
