# Supplementary material for: Cultural framing of giftedness in recent US fictional texts
Source: PLoS One. 2024 Aug 29;19(8):e0307222. doi: 10.1371/journal.pone.0307222 (PMC11361604; doi:10.1371/journal.pone.0307222)
Supplement: Attachment — Submitted filename: Reply to editor Apr 24.pdf [file pone.0307222.s002.pdf]

Cultural framing of giftedness in recent US fiction

This paper investigated the cultural meanings associated with ‘gifted’ and/or ‘giftedness’ through the analysis of US fiction corpus. The authors have endeavoured to review the terms apart from the ordinary angle of perceptions and attitudes surveys usually concerning the context of formal education. The Corpus of Contemporary American English (COCA) was used as a major source for the recent decades of US fiction. Researchers aims to locate the notions of the highlighted terms by examining the salient words types (i.e., themes) used before and after the terms and the sentiment when these expressions are used in texts. Outcomes from the keyword identification procedure shows that in general those terms are located mostly with positive meanings. The obtained meanings were mainly around ten possible themes such as, potent natural ability and written communication. For the sentiment analysis the authors used a lookup dictionary for positive and negative emotions to examine the extract corpus. Results indicate a significant difference for the positive emotion words comparing to the negative ones.

Overall I find the study well designed and executed and the paper well-presented and thorough. I find the research questions to be answered appropriately by the method and the analysis used. Nonetheless, I am a little more skeptical than the authors regarding the manual coding process to remove unrelated usage of ‘giftedness’, the possibility of errors being included cannot be ruled out. Therefore, it may be more reliable to use an electronic instrument for the analysis or at least use another reviewing eye for their manual coding procedure. The same would be applicable for the process of resolving the encoding errors. Moreover, authors need to be clear with regard to the tool used to locate the found results for instance, when limiting their keyword investigation to word tokens occurred in target and reference corpus. Also, when they built a matrix for target and reference corpus, is there any software assisting in the step? This should help in study reduplication in a different context and clarifying the process followed for general readers and unexperienced researchers. In addition, I certainly understand the authors attempts to advertise their gap knowledge as cultural products are essential in indicating the views on gifted people (i.e., giftedness), however, the educational setting surveys are to complete the picture and though shouldn’t appear in contracting place in the paper.

Ultimately, I think the paper has a great potential to establish several more important bridges on using corpus to investigate a nation views regarding specific term. The study is interesting and generally well though through, and there is certainly merit in examining if general minor corrections mentioned above and the ones to be followed were addressed.

Minor issues

Line 125: try to break your literature into more titles for instance, line 125 should be something like ‘studies on cultural products on/of giftedness’

Line 196: can be inserted on your line 220 under the present research with doing necessary corrections/amendments.

Line 321: in the sentence ‘In unclear cases’, it is unclear what do you mean (rephrase) the sentence.

Lines 331-332: have you resolved encoding errors manually? Clarify.

Line 354: better write it as ‘we will describe the implemented analytical procedures’.

Line 355: it would be better to refer to research questions one or two by using RQ1 or RQ2 starting from its second appearance on text.

Lines 354-355 and 359-365: you have used the word ‘implement’ a lot try to find another synonymous.

Line 386: what the tool you used to limit your keyword investigation?

Lines 389-390: is there a software assisting you in built matrix, if so please specify.

Line 390: what (2-) is used for? You mean at the two columns?

Line 393: you mentioned “we then assess using an inferential statistical measure”, what is that? Again, have you completed through a software? Always draw the complete picture for your readers.

Lines 400-401: what is the Principal Component Analysis (PCA)? Who invented it and why it would be reliable in such a case?

Line 402: “was not needed” why? remind the reader of your aim.

Line 414: at the end of this sentence, it seems would need to continue speaking about second or third followed procedure. Therefore, whether you delete (first) from the sentence, so the reader wouldn’t expect the continuity of ideas or add next processe/s to the same paragraph.

Line 424: what are the bases for your cutoff point? You seem to provide an answer at line 430 so try to relocate the sentence.

Lines 432-436, try to break the sentence for better comprehension.
Line 437: “The document-feature matrix consisted of 187,406 values of 0”, you mean at 187,406 text instances you have got 0 word type? It doesn’t make sense as you have found more with lower text instances, please defend/explain your finding.
Line 438-439: try to differentiate between text instances and values found with paratheses for instance, 5.813 (1 value) or any other style.
Line 441: write the numbers/equation in a better way.
Line 464: how you entered the target words in the dictionary, manually?
Lines 566-568: unclear sentence, rewrite please.
Lines 610-611: rewrite the sentence to reduce the use of ‘for’ in it.
Lines 614-617: you have already mentioned the components above so, no need for the repetition in these lines.
Lines 697-698: “it is reasonable in the context of exploratory research”, according to what you have built this conclusion?
Line 784: “to live on in” ?
Line 843: replace ‘within’ with ‘in’ to avoid repetition.
Line 856: “as such, such as” replace one.
Lines 869-873: a prolonged sentence, try to break it up.
