# Supplementary material for: Cultural framing of giftedness in recent US fictional texts
Source: PLoS One. 2024 Aug 29;19(8):e0307222. doi: 10.1371/journal.pone.0307222 (PMC11361604; doi:10.1371/journal.pone.0307222)
Supplement: Attachment — Submitted filename: Response to Reviewers.pdf [file pone.0307222.s003.pdf]

Universität Regensburg

Dr. Daniel Patrick Balestrini, Lecturer
Chair of School Research, School Development, and Evaluation
Faculty of Human Sciences
Institute of Education Sciences
Telephone +49 941 943–3652
Telefax +49 941 943–1993
Universitätsstraße 31
D–93053 Regensburg
Germany
daniel-patrick.balestrini@ur.de

Universität Regensburg ▪ D–93040, Regensburg, Germany

Luobing Dong
Academic Editor
PLOS ONE

26 June 2024

Dear Luobing Dong:

Thank you for your email and feedback of 16 May 2024 on our submission PONE-D-24-06759! Please also express our thanks to the unnamed reviewer who provided us with detailed feedback. Your feedback, the additional editor comments, and the reviewer's feedback were very helpful for improving our manuscript.

In addressing the points raised, we needed to revise our code documentation and rerun the code pipeline. We created a revised version of the repository we submitted with our first manuscript version in February. The revised repository will be hosted alongside the first one and is located here. Access will be grated to editors and reviewers upon request. We plan to make the repository public once our manuscript has been accepted.

Below my name, please find our responses on all the points raised by you and the reviewer. We reproduced the comments in ***boldface italics,*** followed by our responses in regular font.

Thank you for reviewing our revised manuscript!

Sincerely,

Daniel Patrick Balestrini

Daniel Patrick Balestrini

Comments by the Academic Editor

Thank you for submitting this material. Based on the reviewer's feedback, we need to make the following revisions:

Thank you for your comments as academic editor! We attended to all comments.

We need to further elaborate the perspective and provide more supporting evidence. Please add relevant examples and data to strengthen your argument.

We attended to all of the instances in which the reviewer requested more elaboration/explanation, and this involved adding an additional source of supporting data to our manuscript. For example, we …

- Elaborated the relationship between our work and earlier work by adding additional references to how our approach complements earlier work in the Introduction (see Lines 109–110 in the change-tracked revised manuscript) and in the Concluding Remark (see Lines 1,044–1,045);
- Added an external inter-rater agreement test after our manual coding procedure (to show that our coding procedure was reliable);
- Revised how we deal with cleaning of the original corpus data to be more systematic;
- Resolved two issues raised by the reviewer regarding our methods (tools, building of our matrix) by adding additional references to our Technical Supplement (S1 File); and
- Improved our documentation of the procedure we used to identify a word-inclusion threshold for our document–feature matrix.

You will find longer descriptions of these revisions among our responses below.

In the conclusion section, we need to summarize the key points of the article more clearly and concisely. Try to encapsulate the main thesis and core conclusions in a more succinct manner.

We revised our conclusion section (Concluding Remark) to summarize the key points of the article more clearly and succinctly.

Overall, please carefully check the logic and coherence of the article to ensure smooth transitions between paragraphs. Also pay attention to the accuracy and literary quality of the phrasing.

We reread the entire manuscript to check its logic and coherence and to ensure smooth transitions between paragraphs. We made numerous small changes to improve clarity, reading flow, and the accuracy and consistency of wordings and phrasing. For example, we standardized our use of the terms ‘fiction’ and ‘fictional texts’ with ‘fictional texts’ throughout the entire manuscript.

A native speaker of English read the manuscript and gave us feedback on phrasing and idiomatic style. This helped us to root out typos, revise awkward turns of phrase, and avoid wording inconsistencies.

Journal Requirements:

1. When submitting your revision, we need you to address these additional requirements.

Please ensure that your manuscript meets PLOS ONE's style requirements, including those for file naming. The PLOS ONE style templates can be found at

https://journals.plos.org/plosone/s/file?id=wjVg/PLOSOne_formatting_sample_main_body.pdf and

https://journals.plos.org/plosone/s/file?id=ba62/PLOSOne_formatting_sample_title_authors_affiliations.pdf

Thank you for directing our attention to these resources. We reviewed both documents and revised the headings, tables, figures, bibliography, and file names in accordance with the PLOS ONE standards. We processed our figure images with the PACE image-editing tool to ensure they comply with PLOS ONE image guidelines.

2. Please review your reference list to ensure that it is complete and correct. If you have cited papers that have been retracted, please include the rationale for doing so in the manuscript text, or remove these references and replace them with relevant current references. Any changes to the reference list should be mentioned in the rebuttal letter that accompanies your revised manuscript. If you need to cite a retracted article, indicate the article's retracted status in the References list and also include a citation and full reference for the retraction notice.

We reviewed the reference list and made formatting adjustments to comply with the PLOS ONE (Vancouver) reference system. In one case, we had accidentally referenced the same publication with two different numbers. We removed the duplicate reference. We added one new reference in response to reviewer feedback. The added reference is noted in our response to the reviewer (below), and both the removed duplicate reference and the added reference are pointed out in the change-tracked revised manuscript. Finally, we realized that we had included one parenthetical literature citation in the manuscript. We provided a proper PLOS-ONE style reference for this citation.

Additional Editor Comments:

This paper investigated the cultural meanings associated with 'gifted' and/or 'giftedness' through the analysis of US fiction corpus. The authors have endeavoured to review the terms apart from the ordinary angle of perceptions and attitudes surveys usually concerning the context of formal education. The Corpus of Contemporary American English (COCA) was used as a major source for the recent decades of US fiction. Researchers aims to locate the notions of the highlighted terms by examining the salient words types (i.e., themes) used before and after the terms and the sentiment when these expressions are used in texts. Outcomes from the keyword identification procedure shows that in general those terms are located mostly with positive meanings. The obtained meanings were mainly around ten possible themes such as, potent natural ability and written communication. For the sentiment analy-

sis the authors used a lookup dictionary for positive and negative emotions to examine the extract corpus. Results indicate a significant difference for the positive emotion words comparing to the negative ones.
Overall I find the study well designed and executed and the paper well-presented and thorough. I find the research questions to be answered appropriately by the method and the analysis used.

Thank you for your positive feedback and your review of our manuscript! We appreciate your comments, questions, and suggestions and have revised our manuscript to address all of the concerns raised by the academic editor and the reviewer. We respond to all points below, point by point.

Nonetheless, I am a little more skeptical than the authors regarding the manual coding process to remove unrelated usage of 'giftedness', the possibility of errors being included cannot be ruled out. Therefore, it may be more reliable to use an electronic instrument for the analysis or at least use another reviewing eye for their manual coding procedure.

Thank you for pointing this out!

To address this concern, we recruited an independent rater who independently coded a systematic sample of 200 of the 9,151 passages we manually coded. The 200 passages we used for the external coding were spread out evenly over the 9,151 passages. The rater worked independently following the procedure we originally used (and described in the original manuscript and the Technical Supplement). The independent-rater test of our coding procedure showed that our manual coding procedure was highly accurate, with an agreement of 93%. We added information about this to the manuscript and details of the coding procedure to the revised Technical Supplement. See Lines 336–344 in the change-tracked revised manuscript and pp. 29–32 in the Technical Supplement. We have also included the instruction letter we sent to the manual coder and the file the coder returned in the revised version of our data repository. We added one additional reference to the literature providing a basis for our external-rating procedure (1), which is listed as Reference Number 78 in the change-tracked revised manuscript (see Lines 1,275–1,277).

The same would be applicable for the process of resolving the encoding errors.

Thank you for pointing this out!

We developed and implemented a more systematic approach to dealing with encoding issues. We revised the first part of our data-cleaning procedure. See Lines 347–362 in the change-tracked revised manuscript and Section 7.2 (pp. 43–53) in the revised Technical Supplement.

Because the new procedure changed the corpus on which we based our analyses, we reran the entire code pipeline of the study. This led to small changes in the values we report for the analyses. All such changes are indicated in the change-tracked revised manuscript.

Moreover, authors need to be clear with regard to the tool used to locate the found results for instance, when limiting their keyword investigation to word tokens occurred in target and reference corpus.

For all steps, we used existing tools (code packages) running in R and, in a few cases, in Python as well as custom-built functions. For example, a key package of tools for text mining are the functions provided by the Quanteda suite of text-mining tools. The Technical Supplement includes all the code and tools we used to carry out the study. It explains how to use the tools and provides comments for the custom functions we wrote. Readers should be able to replicate our study by following the Technical Supplement and employing the same tools described therein. The supplement also includes version information and citations with bibliographic references (at the back of the supplement) for all the existing text-mining tools we employed.

In the changed-tracked revised manuscript, we added two additional remarks referring readers to the Technical Supplement to help readers find the information on our tools and methods. In the changed-tracked revised manuscript, we added a brief reference to the supplement at Lines 279–280 and a longer explanation of the supplement in Lines 376–379.

Also, when they built a matrix for target and reference corpus, is there any software assisting in the step? This should help in study reduplication in a different context and clarifying the process followed for general readers and unexperienced researchers.

We explain the matrix creation in detail and provide a bibliographic reference for the text-mining function we used (the Quanteda dfm() function) in the supplement. See the first sentence in Section 9 and Listing 9.1 on p. 71 in the revised Technical Supplement.

We added an extra remark to the main manuscript when we mention creating the matrix for the keyword identification procedure. The new remark directs readers to the supplement regarding the creation of the matrix. See the new parenthetical remark in the revised changed-tracked version of the main manuscript at Lines 424–425.

We understand that not providing the names of such functions and tools in the main manuscript means the reader of the main manuscript will have to cross-check technical details about functions and the like in the supplement. However, we decided it was a better trade-off for the readability of our main manuscript.

In addition, I certainly understand the authors attempts to advertise their gap knowledge as cultural products are essential in indicating the views on gifted people (i.e., giftedness), however, the educational setting surveys are to complete the picture and though shouldn't appear in contracting place in the paper.

Thank you for this comment.

To make clear that we view our approach not as based a contradictory claim but as one offering an additional perspective, we frame the investigation of cultural products as a "complementary method" (see Line 105 in the change-tracked revised manuscript). Then, in the Discussion, we describe how our approach and findings elaborate or complement the picture created by earlier educational-settings surveys. In Lines 890–892 of the changed-tracked revised manuscript, we explain that our findings do not invalidate earlier findings based on surveys done in educational settings, but rather provide a complementary perspective: "This does not imply an invalidation of earlier findings indicating ambivalent or negative appraisals of the concept […] but requires a careful explanation." After that remark, we then provide an explanation of how our findings build on the earlier findings (rather than invalidating them).

To address your concern, we added additional remarks drawing readers' attention to the complementary nature of our approach and findings:

- In the Introduction, we added an additional sentence in Lines 109–110 (in the change-tracked revised version) to elaborate how our work builds on earlier findings by providing additional context.
- In the Concluding Remark, we added an additional adverbial phrase to remind readers at the very end of the manuscript that we view our research as a complementary approach providing additional insights complementing the earlier work: "To shed more light on the cultural framing of giftedness," (see Line 1,037–1,038 in the changed-tracked revised manuscript).

Ultimately, I think the paper has a great potential to establish several more important bridges on using corpus to investigate a nation views regarding specific term. The study is interesting and generally well though through, and there is certainly merit in examining if general minor corrections mentioned above and the ones to be followed were addressed.

Thank you very much for your positive assessment of our work and your helpful feedback on our manuscript. We appreciate all the points you have made and have endeavored to improve the manuscript by addressing all of them.

Minor issues

Line 125: try to break your literature into more titles for instance, line 125 should be something like 'studies on cultural products on/of giftedness'

Thank you for this helpful comment. We added two level-3 headings to break the literature review in the section to which you refer into smaller units of information. You will find the new sub-sub-headings at Lines 117 and 129 in the revised change-tracked manuscript.

Line 196: can be inserted on your line 220 under the present research with doing necessary corrections/amendments.

Following your suggestion, we moved the leve-2 heading "The present research" one paragraph up (earlier) in the manuscript. The heading is now located on Line 203 in the revised change-tracked manuscript.

Line 321: in the sentence 'In unclear cases', it is unclear what do you mean (rephrase) the sentence.

We rewrote the phrase to provide a better explanation of what we mean. We deleted "In unclear cases" and instead wrote "When the 51-word text windows provided insufficient context for determining the sense of the lexeme "gift,"." For the manual coding process, it was usually obvious which sense of the word was being used by looking at a few words to the left and right of the node word 'gift.' You will find the revision on Lines 333–335 of the changed-tracked revised manuscript. In some cases, more context was needed. For that reason, the manual coding process included longer excerpts with 100 words before and after 'gift.' We also explain this point in the Technical Supplement. See p. 28 (second paragraph from top) in the revised supplement.

Lines 331-332: have you resolved encoding errors manually? Clarify.

Yes, we used a manual process. However, in light of the earlier related point (above), we revised our procedure here. See our detailed explanation above in this letter.

Line 354: better write it as 'we will describe the implemented analytical procedures'.

Following your suggestion, we changed the tense of the statement from simple present to future by adding a "will" in Line 387 of the revised change-tracked manuscript.

Line 355: it would be better to refer to research questions one or two by using RQ1 or RQ2 starting from its second appearance on text.

We adopted this suggestion throughout our manuscript. At the first occurrence of "Research Question 1" (Line 246 in the revised change-tracked manuscript) we introduce the abbreviation "RQ1." At the first occurrence of "Research Question 2" (Line 249 in the revised change-tracked manuscript) we introduce the abbreviation "RQ2." We maintained the written-out forms of both terms when "Research Question" appears in a heading, but added the abbreviation to the heading to help readers remember the abbreviation (see Lines 539 and 738).

Lines 354-355 and 359-365: you have used the word 'implement' a lot try to find another synonymous.

We changed “implemented” to “performed” on Lines 389 and 393 in the change-tracked revised manuscript.

Line 386: what the tool you used to limit your keyword investigation?

We used the filter() function from the dplyr package in R. The dplyr package is contained within the larger tidyverse package, also in R. For our filter() call, see Line 9 in Listing 9.2 in the Technical Supplement (p. 72 in the revised Technical Supplement). We provided a bibliographic reference for the tidyverse package in the supplement (see Reference 35 in the revised Technical Supplement).

Lines 389-390: is there a software assisting you in built matrix, if so please specify.

We constructed the document–feature matrix with the Quanteda dfm() function. In the revised Technical Supplement, see the first sentence in Section 9 and Listing 9.1 on p. 70. The dfm() call is located in the listing on Lines 5–7. We provide a bibliographical reference for the Quanteda package (and with that for the dfm() function) in the supplement. See Reference 7 in the revised Technical Supplement. The actual keyword processing was done shortly thereafter, on Line 1 of Listing 9.2 (p. 59 in the original supplement, p. 71 in the revised supplement) with the textstat_keyness() function, which is part of the quanteda.textstats package (see Reference 8 in the revised supplement).

Line 390: what (2-) is used for? You mean at the two columns?

The reference in Lines 390–391 of the original manuscript (= Lines 424–425 in the revised change-tracked manuscript) to “columns 2–84,053” was describing the columns in the matrix containing occurrences for all of the 84,052 word tokens included in the matrix. The character following the “2” means ‘from … to.’ In other words, from Column 2 to Column 84,053. In our description, we were counting Column 1 as the column with the row names to simplify the explanation. However, this turned out to be confusing, not helpful, we think. After reviewing the matrix in light of your question, we revised the explanation to read “columns 1–84,037.” This should be more intuitive sense for readers. The total number of columns in the matrix changed slightly during the revision—from 84,052 to 84,037—because of the revised procedure for dealing with the irregular wordings (described above).

Line 393: you mentioned “we then assess using an inferential statistical measure”, what is that? Again, have you completed through a software? Always draw the complete picture for your readers.

The inferential statistical measure we used in the keyword analysis is the log-likelihood ratio G^2 with the Williams correction. It is the most common measure used for assessing the keyness of word types in keyword analyses. We explain the measure and why we are using it in the Keyword Identification subsection of the Results section. See Lines 482–489 (original version of the manuscript) / Lines 548–555 (change-tracked revised version of the manuscript). You will

find our implementation of the measure in the Technical Supplement: See Line 1 in Listing 9.2 (p. 71) in the revised version and Reference 8 (in the supplement).

The remark at Line 393 (Line 428 in the change-tracked revised manuscript) is part of the "Analytical procedures and measurement instrument" section. The section describes broadly the procedures and measurement instruments without going into the statistical measures we use later on in the analyses. However, we provide details regarding statistical measures in the "Results" section as well as, for background information, in the Technical Supplement.

Lines 400-401: what is the Principal Component Analysis (PCA)? Who invented it and why it would be reliable in such a case?

Principal component analysis (PCA) is a dimension-reduction technique used to identify patterns or themes in data sets by identifying components that explain the greatest amount of variance in the data. PCA is used in a variety of fields. In quantitative text analysis, it is an established technique for revealing patterns of meaning in documents once the documents have been translated into a document–feature matrix. Compared to non-deterministic methods of identifying meanings in documents such as latent Dirichlet allocation (LDA), PCA has the advantage of being a deterministic procedure, allowing for precise replication of PCA-based findings.

PCA goes back a long way, for example to work done by Karl Pearson in 1901 (https://en.wikipedia.org/wiki/Principal_component_analysis). For our study, we cited two recent methods publications that explain and illustrate the use of PCA in quantitative text analysis. (2, 3)

In our original manuscript, we provided background on PCA and references to key methods literature. After considering your comment, we added two additional sentences providing a better explanation of what PCA is about and how it is employed in quantitative text analysis. See the new remark on Lines 436–441 in the revised change-tracked version of the manuscript.

Line 402: "was not needed" why? remind the reader of your aim.

We revised the beginning of this sentence to connect back to our expanded description of the PCA method (see previous response) and added more explanation. See Lines 440–441 in the revised change-tracked manuscript. With the expanded explanation of what PCA is, the reasoning as to why we did not need the reference corpus for the PCA should now be clear.

Line 414: at the end of this sentence, it seems would need to continue speaking about second or third followed procedure. Therefore, whether you delete (first) from the sentence, so the reader wouldn't expect the continuity of ideas or add next processe/s to the same paragraph.

Thank you for pointing this out! We concur that our ordering was hard to follow. We changed the order of the steps in our revised manuscript:

- We provide the background on the PCA (Lines 436–453 in the change-tracked revised manuscript).
- Then we describe the three steps involved in transferring our text data into a binary document–feature matrix (Lines 454–509 in the change-tracked revised manuscript).
- Finally, we provide the details about our parallel analysis (Lines 510–513 in the change-tracked revised manuscript), which must be done after preparing the document–feature matrix for the PCA but prior to running the PCA.

Line 424: what are the bases for your cutoff point? You seem to provide an answer at line 430 so try to relocate the sentence.

We based the determination of our exclusion cutoff (inclusion threshold) on a recent method recommendation (3). We revised and expanded our remark about the inclusion threshold in the main manuscript (see Lines 462–471 in the revised changed-tracked manuscript), added a reference to the Technical Supplement regarding additional details, and expanded our step-by-step documentation of how we determined our inclusion threshold in Section 10.1 (pp. 89–102) of the revised Technical Supplement.

Lines 432-436, try to break the sentence for better comprehension.

Thank you for pointing this out! We revised the wording and broke up the sentence in these lines to make it easier to follow. See Lines 478–485 in the changed-tracked revised manuscript.

Line 437: "The document-feature matrix consisted of 187,406 values of 0", you mean at 187,406 text instances you have got 0 word type? It doesn't make sense as you have found more with lower text instances, please defend/explain your finding.

We revised the explanation of the filtered document–feature matrix and of Table 2 to make it easier to follow. See Lines 487–505 in the change-tracked revised manuscript. The values are slightly different than in the original manuscript because of the changed procedure for cleaning the original corpus text (described above).

Line 438-439: try to differentiate between text instances and values found with paratheses for instance, 5.813 (1 value) or any other style.

This problem was caused, we think, by our poor wording here. We revised this according to our previous response. See the revised explanation on Lines 487–505 in the change-tracked revised manuscript.

Line 441: write the numbers/equation in a better way.

We revised the wording of the table note also to address this concern. See Lines 499–505 in the change-tracked revised manuscript.

Line 464: how you entered the target words in the dictionary, manually?

We used an existing dictionary, the Lexicoder Sentiment Dictionary (LSD), and did not enter any additional words into the dictionary. We explain our use of an existing look-up dictionary in the paragraph starting on Line 449 (original manuscript) / Line 515 (revised manuscript). Also, in the Technical Supplement, we show readers how to call the dictionary and note its sources (see Listing 12.2 and the remark before that listing on p. 144 in the revised supplement). We then extracted, separately, the positive and negative sentiments from the existing LSD dictionary object (not created by us). First, we extracted the positive sentiments (words) from the LSD and stored these in a separate object in R (see p. 144–146 and Listing 12.4 in the revised supplement); we then implemented a lookup procedure with the positive sentiments and our text corpus (see Listing 12.5 on p. 146 of the revised supplement). Then we did the same with the negative sentiments (see Listings 12.7 and 12.8 on pp. 147–148 in the revised supplement).

Should your question be about the language data we are examining with the LSD: We explain the process of reloading the non-lemmatized version of our *gift** and *only* corpus at the beginning of Section 12 in the Technical Supplement (see p. 144 and Listing 12.1 in the revised supplement).

Lines 566-568: unclear sentence, rewrite please.

We simplified the sentence. See Line 640–642 in the changed-tracked revised manuscript.

Lines 610-611: rewrite the sentence to reduce the use of 'for' in it.

We rewrote the sentence to include 'for' less often. See Lines 684–687 in the change-tracked revised manuscript.

Lines 614-617: you have already mentioned the components above so, no need for the repetition in these lines.

We deleted the detailed list and added a brief reference back to Table 4. See Lines 688–692 in the change-tracked revised manuscript.

Lines 697-698: "it is reasonable in the context of exploratory research", according to what you have built this conclusion?

The significant finding is worth noting as an exploratory (rather than confirmatory) finding. We provide more background on this perspective in the discussion and limitation sections. We rewrote the sentence, deleting the word 'reasonable' to avoid uncertainty about what qualifies as 'reasonable.' See Lines 776–779 in the revised change-tracked manuscript.

Line 784: "to live on in" ?

The term ‘to live on’ is a phrasal verb meaning to continue to exist (https://www.merriam-webster.com/dictionary/live%20on). The preposition ‘in’ is part of an adverbial phrase explaining where this process is happening, namely, in US fictional texts. We would prefer to retain ‘to live on in …’ as it captures the circumstance somewhat better than if we write ‘continues to exist.’ However, if the reviewer or editor finds the turn of phrase still awkward despite our explanation, we are happy to rewrite it.

Line 843: replace ‘within’ with ‘in’ to avoid repetition.

We replaced the second ‘within’ with ‘in’ as requested. See Line 929 in the change-tracked revised manuscript.

Line 856: “as such, such as” replace one.

We replaced “such as” with “including.” See Line 942 in the changed-tracked revised manuscript.

Lines 869-873: a prolonged sentence, try to break it up

We broke the sentence up into two shorter sentences. See Lines 955–960 in the change-tracked revised manuscript.

Literature Cited

1. Tinsley HE, Weiss DJ. Interrater reliability and agreement. In: Tinsley HEA, Brown SD, editors. Handbook of applied multivariate statistics and mathematical modeling. San Diego: Academic Press; 2000. p. 95–124.
2. Boyd RL. Psychological text analysis in the digital humanities. In: Hai-Jew S, editor. Data Analytics in Digital Humanities. Cham: Springer International Publishing; 2017. p. 161–89.
3. Markowitz DM. The meaning extraction method: An approach to evaluate content patterns from large-scale language data. Front. Commun. 2021; 6.
